# A qualitative assessment of using ChatGPT as large language model for scientific workflow development

**DOI:** 10.1093/gigascience/giae030

**Published:** 2024-06-19

**Authors:** Mario Sänger, Ninon De Mecquenem, Katarzyna Ewa Lewińska, Vasilis Bountris, Fabian Lehmann, Ulf Leser, Thomas Kosch

**Affiliations:** Department of Computer Science, Humboldt-Universität zu Berlin, 10099 Berlin, Germany; Department of Computer Science, Humboldt-Universität zu Berlin, 10099 Berlin, Germany; Department of Geography, Humboldt-Universität zu Berlin, 10099 Berlin, Germany; Department of Forest and Wildlife Ecology, University of Wisconsin–Madison, Madison, WI 53706, USA; Department of Computer Science, Humboldt-Universität zu Berlin, 10099 Berlin, Germany; Department of Computer Science, Humboldt-Universität zu Berlin, 10099 Berlin, Germany; Department of Computer Science, Humboldt-Universität zu Berlin, 10099 Berlin, Germany; Department of Computer Science, Humboldt-Universität zu Berlin, 10099 Berlin, Germany

**Keywords:** large language models, scientific workflows, user support, ChatGPT

## Abstract

**Background:**

Scientific workflow systems are increasingly popular for expressing and executing complex data analysis pipelines over large datasets, as they offer reproducibility, dependability, and scalability of analyses by automatic parallelization on large compute clusters. However, implementing workflows is difficult due to the involvement of many black-box tools and the deep infrastructure stack necessary for their execution. Simultaneously, user-supporting tools are rare, and the number of available examples is much lower than in classical programming languages.

**Results:**

To address these challenges, we investigate the efficiency of large language models (LLMs), specifically ChatGPT, to support users when dealing with scientific workflows. We performed 3 user studies in 2 scientific domains to evaluate ChatGPT for comprehending, adapting, and extending workflows. Our results indicate that LLMs efficiently interpret workflows but achieve lower performance for exchanging components or purposeful workflow extensions. We characterize their limitations in these challenging scenarios and suggest future research directions.

**Conclusions:**

Our results show a high accuracy for comprehending and explaining scientific workflows while achieving a reduced performance for modifying and extending workflow descriptions. These findings clearly illustrate the need for further research in this area.

**Key Points**

- We explore large language models (LLMs) to support users who develop scientific workflows.
- We are the first to conduct user studies involving domain experts.
- We conduct 3 studies to assess LLMs in scientific workflow comprehension, adaptation, and extension.
- Our results indicate that LLMs efficiently interpret workflows.
- Our results show room for improvement regarding component adaptation and workflow extension.

## Introduction

Large-scale data analysis pipelines (also known as scientific workflows) are crucial in driving research advances for natural sciences [1]. They are pivotal in accelerating large and complex data analysis on distributed infrastructures and offer essential features, such as reproducibility and dependability [2]. In bioinformatics, for instance, scientific workflows are analyzing the terabyte-large datasets produced by modern DNA or RNA sequencing machines in a wide variety of experiments [3], thereby aiding in building a comprehensive understanding of biological processes and human diseases. Bioinformatics workflows typically include many individual computational steps, such as data preprocessing, extensive quality control, aggregation of raw sequencing data into consensus sequences, machine learning–based tasks for classification and clustering, statistical assessments, and result visualization. Each step is carried out by a specific program, typically not written by the workflow developer but exchanged within a worldwide community of researchers [4]. Execution of a workflow on a distributed infrastructure, in principle, is taken care of by a workflow engine; however, the idiosyncrasies of the different infrastructures (e.g., file system, number and features of compute nodes, applied resource manager, and scheduler) often require workflow users to tune their scripts individually for every new system [5].

However, typical developers of workflows are researchers from heterogeneous scientific fields who possess expertise in their respective domains but often lack in-depth knowledge in software development or distributed computing. They often encounter difficulties understanding the complex implementations of exchanged codes and the deep infrastructure stack necessary for their distributed execution. This situation challenges efficient workflow implementation and slows down or hinders data exploration and scientific innovation processes [6]. Consequently, low human productivity is a significant bottleneck in the creation, adaption, and interpretation of scientific workflows [7].

Parallel to this, there is a well-established field in human–computer interaction focusing on assisting end-user programmers and software development, as highlighted by previous work [8–11], to reduce the perceived cognitive workload and improve the overall programmer performance [12]. Research in this field includes programming-by-demonstration [13, 14], visual programming [15, 16], and natural language instructions [17]. Recent work in this area particularly investigated prospects of general-purpose large language models (LLMs), such as ChatGPT [18], LLaMA [19], and Bloom[20], for supporting end-user programming [21–23] and software development in general [24, 25]. For instance, Bimbatti et al. [21] explore using ChatGPT to enhance natural language understanding within an end-user development environment, assisting nonexpert users in developing programs for collaborative robots. Moreover, White et al. [24] introduce prompt design techniques for automating typical software engineering tasks, including ensuring code independence from third-party libraries and generating an application programming interface specification from a list of requirements. Surameery and Shakor [25] evaluate LLMs for supporting code debugging. However, results from such studies, which focus on standard programming languages, cannot easily be transferred to workflow systems. Workflow scripts mostly call external tools with agnostic names and have little recognizable control structures or protected keywords. Publicly available examples are scarce; for instance, the community repository of the popular workflow system Nextflow [26] currently offers only 55 released workflows [27]. Furthermore, workflows can only be understood when the distributed system underlying their execution is considered, creating dependencies much different from usual programs. Moreover, studies investigating how LLMs support users in data science—the field in which workflows are applied extensively—do not address the unique characteristics of scientific workflows either and are limited to theoretical considerations [28, 29]. Practical studies, especially those involving real users, are badly missing.

In this work, we address these shortcomings by describing 3 user studies in 2 different scientific fields (biomedicine and Earth observation) that evaluate the suitability of ChatGPT for comprehending, modifying, and extending scientific workflows. Specifically, we evaluate the correctness of ChatGPT regarding explainability, exchange of software components, and extension when providing real-world scientific workflow scripts. Our results show a high accuracy for comprehending and explaining scientific workflows but reduced performance for modifying and extending workflow scripts. The domain experts positively assessed the explainability in qualitative inquiries, emphasizing the time-saving capabilities of using LLMs while engineering existing workflows. Overall, our work indicates that general-purpose LLMs have the potential to improve human performance when analyzing complex scientific workflows.

## Background

Previous research investigated how related domains, such as programming, can be augmented using interactive technologies [11, 30]. In contrast to programming, where applications use a single programming language and are often executed on a single system, scientific workflows combine multiple software artifacts on distributed stacks for advanced data processing. We ground the reader by providing a literature review about scientific workflows and introducing large language models, including their utility to facilitate the creation of software artifacts.

### Scientific workflows

**Figure 1:**
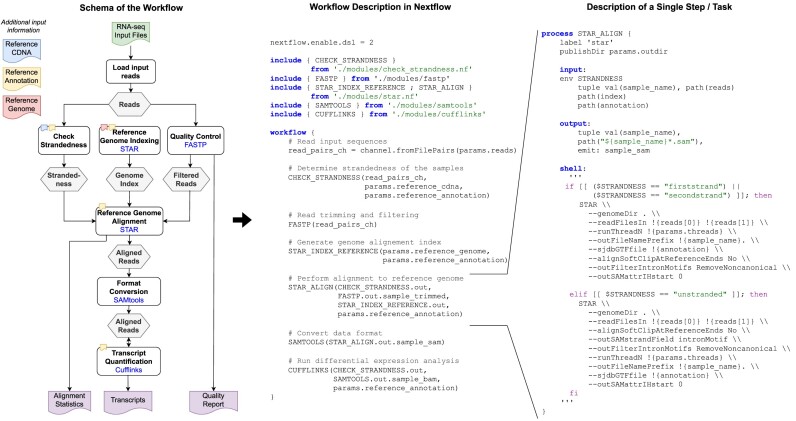
Example bioinformatics workflow for differential gene expression analysis created by a domain expert recruited in our study. The figure highlights the conceptual schema of the workflow (A), its implementation in Nextflow (B), and the implementation of a single step (C), that is, reference genome alignment using the STAR tool. The workflow comprises 6 computational steps in total. For each step, the used tool is given in blue below the task name.

Scientific workflows are widely used by diverse research communities, such as biomedicine [31], astronomy [32], climatology [33], and Earth observation [34], to manage the dataflow and distributed execution of complex analyses, simulations, and experiments. A scientific workflow comprises a series of interconnected computational steps, often with diverse patterns of dependencies, that define how to process and analyze data to reach a particular research objective. Scientific workflows can be regarded as directed acyclic graphs in which the nodes represent computational tasks or operations and edges model dependencies or dataflow between these tasks. An edge from one node to another signifies that the output of the first task is used as input for the second [35]. For example, Fig. 1A illustrates the computational steps, the tools, and the dataflow of a bioinformatics workflow for performing differential gene expression analysis. During workflow execution, a single computation step often involves multiple processes, which are typically executed in a distributed fashion on different machines and batches of the input data, resulting in a much more complex execution graph. Consequently, scientific workflows help facilitate the reproducibility and traceability of data analyses by explicitly outlining the steps and parameters involved [36]. Furthermore, they allow for automation, scaling, and optimization of computational processes, which is especially critical in disciplines dealing with large datasets [37]. The increasing importance of scientific workflows for scientific progress has led to a growing interest in developing more user-friendly tools and methods through the research community. Scientific workflow management systems, like Apache Airflow [38], Galaxy [39], Nextflow [40], Pegasus [41], and Snakemake [42], are specifically developed to support users in designing and executing scientific workflows in various aspects. Key features of such management systems typically include workflow design and composition, (distributed) workflow execution and scheduling, provenance tracking, recovery and failure handling, and resource management [36]. Fig. 1B highlights the implementation of the example workflow as well as a single computational step (see Fig. 1C), that is, reference genome alignment using the STAR toolkit in Nextflow.

Scientific workflows are often reused and adopted for complex data analysis [6]. Most of the time, users of scientific workflows are unaware of the workflow’s internal functionality and technical details. Instead, users of scientific workflows represent domain experts, such as mathematicians, physicians, or bioinformatics, who are experts in their respective domains but not necessarily in programming and interpreting scientific workflows. Existing scientific workflows are implemented and maintained by persons other than the domain user. This reduces the direct interaction with scientific workflows to a minimum, where domain experts only hand in the input data and evaluate the output data. Consequently, domain experts using a workflow often do not have the knowledge to modify, extend, or interpret the details of scientific workflows.

### Large language models

Language models, such as BERT [43], GPT-3 [44], Bloom [20], and PaLM-2 [45], build the foundation of many recent advancements in natural language processing and understanding. These models have billions of parameters and are generally pretrained on vast sets of texts from the web and other repositories, enabling them to encode syntactic and semantic relationships in human language. As a consequence, generative language models, such as ChatGPT, LLaMA [19], and LAMDA [46], can produce machine-generated high-quality text that is indistinguishable from human writing. These generative capabilities have empowered these models to assist in diverse (creative) writing tasks and have been utilized to facilitate a wide range of interactive language-based applications within the human-computer interaction community [47–52]. For instance, WordCraft [47] investigates the utilization of LLMs to aid fiction writers in tasks ranging from transforming a text to resemble a “Dickensian” style to providing suggestions to combat writer’s block. Their findings indicate that writers found such text-generating models beneficial even when the generated text is not perfect. Petridis et al. [48] introduce AngleKindling, an interactive tool that employs LLMs to support journalists exploring different angles for reporting on a press release. Their study with 12 professional journalists shows that participants found the system considerably more helpful and less mentally demanding than competitor brainstorming tools. Other applications include prototyping support [49], generation of titles and synopses from keywords [52], conversational interactions with mobile user interfaces [50], and conceptional blending [51].

Next to their text writing capabilities, language models are further known to retain commonsense knowledge within their training data, effectively transforming them into accessible knowledge stores that can be seamlessly queried using natural language prompts. For instance, experiments with BERT [43] highlight that the model performance is competitive with traditional information extraction and open-domain question answering. Furthermore, recent studies show the potential of using ChatGPT for knowledge base construction, inspired by the fact that these language models have been pretrained on vast internet-scale corpora that encompass diverse knowledge domains [53]. However, it is worth noting that LLMs are known to frequently generate hallucinations, which are outputs that, while statistically plausible and seemingly believable, are factually incorrect [54, 55].

### Using LLMs to support programming

The ability to generate new text and to reconstruct existing information makes LLMs highly appropriate to support users in software development, as programming often requires not only the creation of novel code segments tailored to current requirements and tasks but also depends on the application of established algorithms, software libraries, and best practices. Accordingly, a large number of papers investigate LLMs specially trained for code generation [56–59] as well as different approaches leveraging these models to provide interactive programmer support [23, 60–62]. For instance, Jiang et al.  [61] discuss GenLine, a natural language code synthesis tool based on a generative LLM and problem-specific prompts for creating or changing program code. The findings from a user study indicate that the approach can provide valuable support to developers. However, they also encounter several challenges, such as participants finding it difficult to form an accurate mental model of the kinds of requests that the model can reliably translate. Similarly, Vaithilingam et al. [62] conducted a user study with 24 participants evaluating their usage and experiences using the GitHub Copilot [63] code generation model while programming. The authors find that the synthesized code often provided a helpful starting point and saved online searching efforts. However, participants encountered issues with understanding, editing, and debugging code snippets from Copilot, resulting in not necessarily improved task completion times and success rates. These findings align with the results of similar studies [64]. However, a controlled experiment in [65] records a positive effect of code generators when used in introductory programming courses for minors.

The use of generative LLMs and code generators has been scarcely explored in scientific data analysis and not yet for scientific workflows. Liu et al. [23] examine the Codex code generator [56] in the context of data analysis in spreadsheets for nonexpert end-user programmers. Moreover, several studies investigate the utilization for data visualization [66, 67]. For example, the study by Maddigan et al. [66] evaluates the efficiency of ChatGPT, Codex, and GPT-3 in producing scripts to create visualizations based on natural language queries. The studies that have the most overlap with our work regarding the intention to support the design of data analysis pipelines are given by Hassan et al. [67] and Zahra et al. [68]. In the case of the former, ChatGPT is used to build a conversational, natural language–based interface between users and the scikit-learn machine learning framework [69] supporting users in several phases of a machine learning project ranging from initial task formulation to comprehensive result interpretation. For the latter, Laminar, a framework for serverless computing, is proposed, which offers possibilities for code searching, summarization, and completion. However, the framework is solely focused on Python implementations.

## Methods

This section describes the study methodology. We begin by outlining about the general study approach. Then, we explain the research process for each experiment. Based on related work and the objectives of our research, we state the following research questions:

**RQ1:** How performant is ChatGPT for comprehending and explaining scientific workflows?
**RQ2:** How suitable is ChatGPT in suggesting and applying modifications for scientific workflows?
**RQ3:** How efficient is ChatGPT in extending scientific workflows?

### General study design

To answer our research questions, we investigate the capabilities of ChatGPT, a widely used LLM, to comprehend existing workflow descriptions (cf. Study I), to exchange tools used within a workflow (cf. Study II), and to extend a partially given workflow (cf. Study III) using 3 distinct user studies. We select these use cases as understanding the dataflow and the analysis performed is essential for successfully applying scientific workflows. Moreover, exchanging tools and extending a partially given workflow are common use cases in adapting and reusing existing workflows in the work context of domain scientists [6]. For each study, we specially design conversational prompts simulating the interaction between a user working with workflows and ChatGPT. For our studies, we leverage version GPT-3.5 of ChatGPT [70]. We decided to use GPT-3.5 since it is openly available to the public and allows other researchers to reproduce our investigations without additional incurring costs. Moreover, we provide the chat records in the Supplementary Material of this article via the *GigaScience* database (GigaDB) [71]. Additionally, we develop distinct questionnaires for evaluating the output of ChatGPT by the domain experts for each study. While conducting a study, we present a brief overview of the study’s overall goal and the developed questionnaire to the experts. Subsequently, the experts complete the questionnaire independently without the experimenters’ support. This procedure is intended as participants were not pressured by a time limit and could freely allocate their time for the study. Furthermore, we intend to avoid a Hawthorne effect, where participants can provide biased responses due to the presence of observers [72].

### Participants

Throughout all experiments, we recruited 1 expert from bioinformatics and 3 experts working on Earth observation workflows. Using scientific workflows is common in these 2 areas. In bioinformatics, scientific workflows are an important tool for enabling the automation and documentation of complex data analysis processes, ensuring reproducibility and transparency in research [40]. In Earth observation, scientific workflows streamline the complex process of acquiring, processing, and analyzing vast amounts of satellite and sensor data, enhancing the efficiency and accuracy of environmental studies [79]. Hence, scientific workflows have become commonplace in these 2 areas. The professions include postdocs and PhD students working at universities. All participants hold a master’s degree in their profession and several years of experience in their domain. All experts are between 25 and 40 years old (2 female, 2 male).

### Scientific workflows

In our study, we consider a total of 5 different workflows. We summarize the used workflows and their details in Table 1. The workflows are taken from the work context of the recruited experts, given their high degree of familiarity and expertise with them. In bioinformatics, we use 2 workflows that deal with the analysis of genomic data. First, the *crisprseq* workflow, sourced from the *nf-core* repository [27], a hub for best-practice workflows, focuses on analyzing and evaluating gene editing experiments utilizing the CRISPR-Cas9 mechanism for genome engineering. Second, the *RS-STAR* workflow, which was implemented by the recruited domain expert, performs differential gene expression analysis using RNA sequencing data. The *FORCE2NXF-Rangeland* and *FORCE* are 2 implementations of an Earth observation workflow that is concerned with analyzing long-term vegetation dynamics in the Mediterranean using the FORCE toolkit [80], which provides processing routines for satellite image archives. The former is implemented using the Nextflow scientific workflow management system [40, 81] and the latter by leveraging Apache Airflow [38, 82]. The third Earth observation workflow, called *Grasslands*, builds on previous work [83, 84] aiming at understanding differences in long-term changes in-ground cover fractions specific to European grasslands depending on the definition of endmembers (i.e., unique spectral signatures of a specific material or ground cover) approximating these fractions.

**Table 1:** Overview of the used workflows. We examine workflows from 2 scientific domains (i.e., bioinformatics and Earth observation) and 2 workflow systems (Nextflow and Apache Airflow). For each workflow, we report the number of (high-level) steps and used tools and in which study the workflow is used.

Domain	Workflow	Description	#Steps	#Tools	Study
Bioinformatics	WF1:crisprseq [73] (*Nextflow*)	A bioinformatics data pipeline for analyzing and evaluating gene editing experiments utilizing the CRISPR-Cas9 mechanism for genome engineering.	6	9	I
		Repository: [74] (created July 2022)			
	WF2:RS-Star (*Nextflow*)	The general aim of this workflow is to perform differential gene expression analysis using RNA sequencing data.	5	5	I, II, III
		Repository: [75] (created November 2021)			
Earth observation	WF3:FORCE2NXF-Rangeland [34] (*Nextflow*)	This workflow analyzes long-term vegetation dynamics in the Mediterranean using the FORCE toolkit.	9	8	I
		Repository: [76] (created November 2020)			
	WF4:Grasslands (*Nextflow*)	This workflow aims at understanding differences in long-term changes (1984–2022) in ground cover fractions specific to European grasslands.	6	3	I, III
		Repository: [77] (created August 2023)			
	WF5:FORCE (*Apache Airflow*)	This workflow focuses on analyzing long-term vegetation dynamics in the Mediterranean using the FORCE toolkit.	8	8	I
		Repository: [78] (created February 2021)			

### LLM prompting

The choice and design of prompts entered into a LLM has a decisive influence on the output quality [85] and, in our case, on the suitability of ChatGPT for workflow development and implementation. Our prompts are organized first to provide the context, often including the workflow script, followed by the specific question or instruction under investigation. Suppose the workflow is divided into sub-workflows, possibly distributed over several files. In that case, we first specify the main workflow and then all sub-workflows and task definitions in the order they occur in the main workflow. In our research’s initial stages, we experimented with various alternative prompts for each user study, incrementally modifying and enhancing them in response to the outcomes we received. For example, for Study I (i.e., workflow comprehension), we discovered that ChatGPT tends to describe properties of the workflow language or technical aspects instead of workflow characteristics. Such phenomena could be resolved by adding explicit instructions (e.g., “do not explain nextflow concepts”). We refer to the Prompt Design Challenges section for a detailed discussion of prompt design challenges. We stopped this adjustment process after a few iterations as soon as no more of such artificial artifacts were generated. We have refrained from more extensive prompt engineering since workflow designers are domain experts from diverse fields who cannot be assumed to be specialists in developing and tuning prompts. However, we acknowledge that the choice of wording in our prompts influences the results [85]. We discuss the limitations of our work concerning the chosen study design and prompt strategy selection in more detail in the Limitations and Future Work section.

## Study I: Workflow Comprehension

In our first study, we investigate the capabilities of ChatGPT in capturing the actual purpose of a workflow. In other words, we are prompting ChatGPT to explain the purpose of a workflow. In this study, we assess ChatGPT’s quality in comprehending and explaining a workflow’s purpose in a user study involving workflow experts. Understanding the dataflow and the analysis performed constitutes an important aspect of the daily work with scientific workflows. On the one hand, workflows are often precisely adapted to individual research questions, which makes it challenging even for other experts from the same domain to understand them. On the other hand, in many research institutions, an increasing number of legacy workflows whose original authors and contributors are no longer available for maintaining and refining the codes require taking over by new team members. Understanding a workflow is usually a prerequisite for adopting and applying a workflow correctly.

Thus, Study I pursues 3 goals: how well does ChatGPT perform on (i) identifying the domain and the overall objective of the analysis; (ii) reporting the individual computation steps, used tools, their needed input data, produced output data; and (iii) explaining research questions for which these analyses are helpful given the workflow description. The first 2 parts of the study have a reconstructive character, whereas the third is more explorative, requiring ChatGPT to reason beyond the given workflow description. We build a set of 5 different prompts to evaluate ChatGPT’s capabilities concerning the 3 dimensions. When providing the workflow definition in the prompt, delete all comments within the definition to prevent information leakage. Table 2 depicts the developed prompts. For each workflow, all prompts are executed in 1 conversation, enabling ChatGPT to use the input and output of previous prompts as context information. We ask the domain scientists to evaluate answers given by ChatGPT using a feedback questionnaire. The complete questionnaire contains 9 items in total and can be found in full length in the article’s Supplementary Material (A). The questionnaire focuses on the correctness of the prompts regarding the aim of the workflow, the explanation, and the forecast of addressed research questions. For 4 of the 9 items, users rate the generated explanations on a 5-point Likert scale (i.e., 1 = strongly disagree; 5 = strongly agree.). In addition, 3 items comprise quantitative evaluations of how many computational steps are correctly detected, how many utilized software tools and programs are accurately identified, and how many valid follow-up research questions. The remaining question items concern the quality of the explanations of the workflow sequence, the description of the tools used, and the results produced. We add a comment field for each item to report issues and errors in the generated explanations if the domain expert does not apply the content.

**Table 2:** Overview of the used prompts to investigate ChatGPT’s capabilities in capturing the content of a workflow description (Study I). *[workflow-language]* and *[workflow-text]* represent placeholders for the workflow management system (i.e., Nextflow or Apache Airflow) and the workflow description text. All prompts are executed within 1 conversation.

ID	Category	Prompt
P1_1	Overall aim	The following text contains a scientific workflow written in *[workflow-language]: [workflow-text]*.
		Explain from which research area this workflow originates and describe the general aim of this workflow. Don’t explain *[workflow-language]* concepts.
P1_2	Workflow explanation	Explain all individual tasks that are implemented in this workflow. For each task, explain which software programs or tools are used in this workflow to perform the task. Don’t explain *[workflow-language]* concepts.
P1_3	Workflow explanation	Explain the type of input data and the format of the input data needed for this workflow. Don’t explain the workflow itself.
P1_4	Workflow explanation	Explain the overall result of this workflow. For each individual task of the workflow, report the type of data that is produced by this task.
P1_5	Research questions	Explain up to 3 research questions for which this workflow is helpful.

### Results

The results of the expert surveys are presented in the following according to the 3 subcategories of the questionnaire: research area and the overall aim of the workflow, explanation of workflow details, and subsequent research questions.

#### Overall aim of the workflow

**Figure 2:**
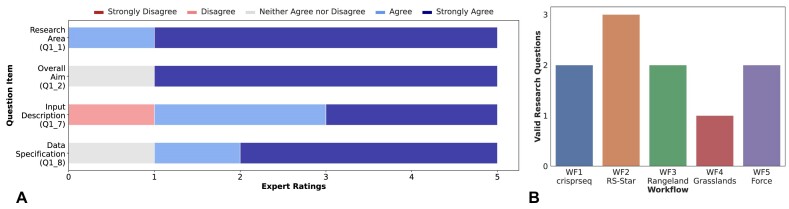
Results from Study I. (A) Rating distribution of the domain experts for ChatGPT’s capability in identifying the research area and overall aim as well as the input and data description of a workflow. The question item identifier (see Supplementary Material (A)) is given in parentheses for each row. (B) Number of valid research questions generated by ChatGPT for the different workflows as assessed by the domain experts (question item *Q1_9*). We prompted ChatGPT to output up to 3 research questions per workflow.

The first 2 rows of Fig. 2A highlight the rating distribution of the domain experts concerning ChatGPT’s explanations for the research area and the overall aim of the workflows. The experts agreed with the explanations generated, indicating a basic understanding of ChatGPT regarding the different workflows (μ = 4.7, σ = 0.68). The lowest agreement, with a score of 4 concerning the research area and 3 for the overall aim, was recorded in the evaluation of the *WF4-Grasslands* workflow. In this case, the expert could not agree with the explanations mainly due to the wrong interpretation of an abbreviation within the workflow description, that is, *FNF* was misinterpreted as *“fraction of non-forest”* instead of *fold and fill*. This misunderstanding resulted in the workflow being explained as examining forest regions rather than grasslands.

#### Workflow explanation

**Figure 3:**
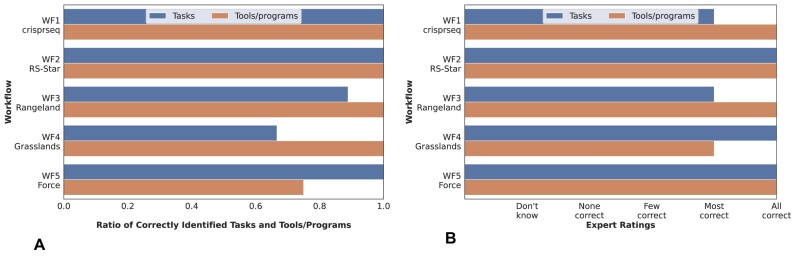
Result statistics highlighting the number of correctly identified tasks and tools of a workflow (A) and their explanation (B). We report separate results per investigated workflow. The results are based on the ratings of the domain experts. Results in (A) correspond to the items *Q1_3* and *Q1_5* and in (B) to *Q1_4* and *Q1_6* of the questionnaire given in the article’s Supplementary Material (A).

In general, the participants regard the quality of the explanations given by ChatGPT as high (see Fig. 3A, B). All computational steps are accurately identified in 3 of the 5 workflow descriptions. Moreover, for 4 of these 5 workflows, every tool employed is correctly detected, but in *WF5-Force*, 2 of 8 were missing. The detailed descriptions of the tasks and tools provided by ChatGPT were also judged to be coherent by the experts. Overall, the worst performance is achieved with the output for workflow *WF4-Grasslands*, for which only 4 of 6 tasks are correctly extracted and only most of the tools are correctly described. These errors are mainly due to follow-up errors that result from the incorrect recognition of the workflow purpose.

The results of the questions items (see Q1_7 and Q1_8 in Supplementary Material (A)), which assess the produced information about the format and type of input and output data of ChatGPT, can be seen in the 2 lower rows in Fig. 2A. Similar to the previous findings, the information generated for 4 of the 5 workflows are evaluated positively (μ = 4.2, σ = 1.0). Again, only the produced information for the workflow *WF4* was assessed as neutral or negative, that is, input description score of 2 (disagree) and data specification of 3 (neither agree nor disagree).

#### Research questions

The last query was concerned with explaining up to 3 subsequent research questions to a given workflow. Figure 2B shows the result of this question item. ChatGPT achieves only moderate performance, generating just for 1 workflow (i.e., *WF2-RS-Star*) 3 valid research questions. In total, out of the 15 generated research questions, 10 were correct. These figures suggest that ChatGPT offers only a reduced performance for more explorative tasks.

## Study II: Workflow Modification

The second study investigates how much ChatGPT can aid domain experts in modifying and tailoring a workflow in our second study. Researchers usually do not start from scratch when developing workflows but typically adapt or reuse parts of existing workflows from the community [6]. This strategy applies in particular to biomedicine, in which workflows are more widespread and have a longer tradition compared to other domains [86]. For example, genomic workflows will often be applied to a broad spectrum of data originating from different sources, each with its distinctive features and characteristics, making it necessary to adjust the workflow definition for more efficient data processing. Moreover, technological advancements, such as in genome sequencing technology [87], lead to new tools specially developed to leverage the capabilities of the new technologies. The continuous integration of new and alternative scientific tools into existing workflows is essential to conduct state-of-the-art research [88].

In our study, we are particularly investigating the exchange of used tools in the bioinformatics workflow *WF2-RS-Star* whose computational scheme is given in Fig. 1A. We select 2 parts of the workflow to be modified:

1. read trimming and filtering (also called read quality control), originally performed by FASTP [89] and
2. reference genome indexing and alignment, carried out by STAR [90].

For assessing the workflow modification capabilities of ChatGPT, we build 4 prompts (see Table 3). The first prompt requests a list of alternative tools for a given workflow step from ChatGPT. The second and third prompts request the recommendation of 2 alternative tools, including an explanation of the suggestion, a comparison of the selected tools with the tool originally used in the workflow script, and their strengths and weaknesses. With the last prompt, the actual rewriting of the workflow to include the selected tool is requested. We test the inclusion of 2 alternative tools per computational task—that is, the prompts *P2_3* and *P2_4* (see Table 3) are carried out once for each tool from the recommendation. Analogous to Study I, we use a questionnaire for evaluating ChatGPT’s output by the biomedical domain expert containing 13 items in total. For most items (9 out of 13), the generated texts and explanations concerning methodical differences or pros and cons of the tools should be rated on a 5-point Likert scale. The remaining questions require numerical ratings (2 items), yes/no answers (1), and free text fields (1). Again, we add a comment field for each item to report issues and errors in the generated explanations if the domain expert does not fully apply the content. The complete questionnaire is given in the article’s Supplementary Material (B). When conducting the study, we also provided the generated workflow scripts to the domain experts and asked them to execute them on their systems. Furthermore, we requested the experts to inspect and correct any nonfunctional scripts. For the latter, we set a time limit of 20 minutes per tool substitution.

**Table 3:** Overview of the used prompts to investigate ChatGPT’s capabilities in swapping used tools in bioinformatic workflows (Study II). Information in square brackets specifies placeholders for concrete information regarding the workflow or the tool to be replaced.

ID	Category	Prompt
P2_1	Tool exploration	The following text contains a *[domain]* workflow written in *[workflow-language]:[main-workflow]*.
		The following snippets contain the source code for the step of the workflow which uses *[tool]* to perform *[step]: [step-source-code]*. Please provide a list of 10 alternative tools to perform *[tool]*.
P2_2	Tool exploration	The following text contains a *[domain]* workflow written in *[workflow-language]:[main-workflow]*.
		The following snippets contain the source code for the step of the workflow which uses *[tool]* to perform *[step]: [step-source-code]*. Alternative tools for *[step]* are: *[list-of-tools]*.
		Which of the tools would you recommend as most suitable alternative for *[step]* in the given workflow? Please name the two alternatives and give an explanation why these tools are especially advisable for the given workflow.
P2_3	Tool exploration	*[original-tool]* and *[alternative-tool]* are two tools for *[step]* in *[domain]* workflows. First, explain the differences between the tools and the underlying approaches. Second, name strengths and weaknesses of both tools.
P2_4	Workflow modification	The following text contains a *[domain]* workflow written in *[workflow-language]:[main-workflow]*.
		The following snippets contain the source code for the step of the workflow which uses the *[tool]* to perform *[step]: [step-source-code]*.
		Please rewrite the code of the workflow and the process to use *[alternative-tool]* instead of *[original-tool]*. The number of parameters of the individual process descriptions may have to be adjusted. Please explain features/options of *[original-tool]* which are not supported in *[new-tool]*.

### Results

The results are presented in the following according to the 2 subcategories of the prompts, including the exploration of alternative tools and workflow modification. We summarize the results of the 2 use cases (i.e., read quality control, reference genome alignment) and each of the 2 alternative tools when reporting the results.

#### Tool exploration

**Figure 4:**
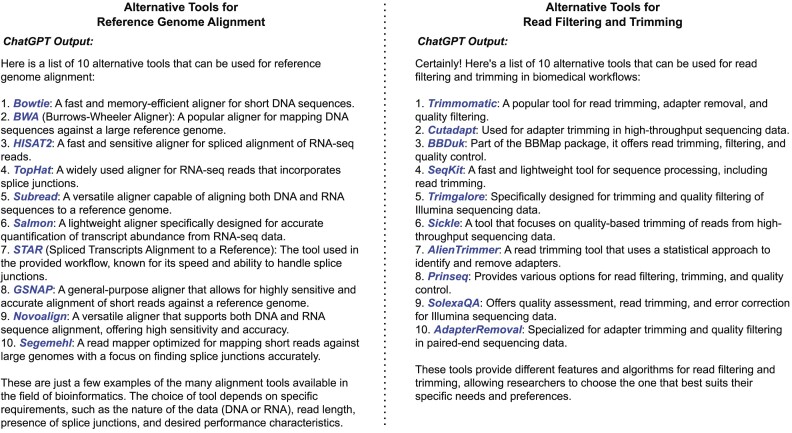
Representation of the output of ChatGPT when requested to provide a list of alternative tools for reference genome indexing and alignment (left) and read quality control (right) using prompt P2_1. All tools are assessed to be valid by the biomedical domain expert.

**Figure 5:**
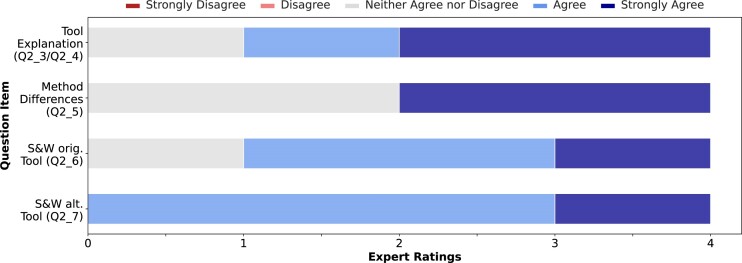
Overview of the rating distribution of the biomedical domain expert for ChatGPT’s capability for explaining alternative tools, methodical differences, and strengths and weaknesses (S&W) of the tools. The question item identifier (see Supplementary Material (B)) is given in parentheses for each row.

When exploring possible alternative tools, ChatGPT showed a good performance, providing a fully valid list of 10 alternative tools using prompt *P2_1* (see Table 3) for both scenarios. The generated output list for both tasks can be seen in Fig. 4. However, the domain expert criticized the alternative tools for reference genome alignment specified Bowtie [91] and not its successor Bowtie2 [92]. From these, ChatGPT recommended HISAT2 [93] and Bowtie as alternatives for genome alignment and Trimmomatic [94] and Cutadapt [95] for read quality control. According to the domain expert, all tools are principally valid substitutions, but Bowtie is a rather inappropriate suggestion since it is not specialized for RNA sequencing data. The results of explaining the tools’ methodological differences, and strengths and weaknesses can be seen in Fig. 5. Essentially, the generated textual explanations were assessed positively, except when describing the methodological differences between the tools. In this case, ChatGPT could offer a convincing explanation in only 50% of the cases. In the other cases, the texts were too general, and differences were not named clearly. In summary, similar to the results from Study I, ChatGPT solves these rather reconstructive tasks well, reaching an average score of 4.1 (min = 3, max = 5, σ = 0.81) when taking the results from items Q2_3 to Q2_7 (see questionnaire in Supplementary Material (B)) into account. These results indicate that ChatGPT could effectively used to explore tools in a given field.

#### Workflow modification

We requested ChatGPT to rewrite the workflow script for each of the 2 recommended alternative tools in both use cases, resulting in 4 modified workflows in total. Table 4 summarizes the results achieved. First of all, none of the generated workflow scripts was entirely correct. Only in 1 of the 4 cases, the generated script was (at least) syntactically valid (i.e., if replacing FASTP with Trimmomatic for read quality control, and the domain expert could execute it without further adaptations). However, the script was not a semantically correct modification of the original workflow script since one particular quality control routine (i.e., PolyG trimming) was not reflected in the adopted script. This issue also occurred for Cutadapt, the other alternative tool for quality control. For both tools, ChatGPT reported in the explanation text that the tools do not support this feature; however, in reality, they do. This failure could be interpreted as a kind of LLM hallucination [54]. The second task, reference genome indexing and alignment, revealed different issues than the first. Here, the main problem was the correct linking of the 2 subparts of the task, first the index generation and then the computation of the actual alignment. For the former, each tool specifies and uses its distinct data format and defines how to store the index (e.g., saving it in 1 or multiple files). However, the storing strategy also affects how the output of the indexing task has to be passed on to the input of the alignment computation. In the scripts generated by ChatGPT, the actual step descriptions to invoke indexing and alignment by the tools were (generally) valid. However, the linking of these two needed to be corrected. For example, Bowtie saves its index in multiple files sharing a common file name prefix, which has to be specified as a parameter during alignment. However, in the modified script, the list of all files of a specially created directory was passed to the alignment process. For Bowtie, this problem could be easily fixed by the domain expert, but for HISAT2, it was not that trivial and hence could not be solved in the given time budget of 20 minutes.

**Table 4:** Overview of the results of the workflow modification use case in which the tools performing a specific task are replaced by alternative ones. For each task, we provide the original tool (in parentheses) and the investigated alternative ones suggested by ChatGPT. For each combination, we highlight (✓ = yes, × = no) whether the generated workflow script could be executed (Exec.), whether it is semantically valid (Val.), and whether it could be fixed within 20 minutes (Fix). For the latter, (✓) indicates cases where the script could be fixed to be executable but not entirely semantically correct. Moreover, we provide excerpts from the domain expert’s comments.

Task	Alt. tool	Exec.	Val.	Fix	Problems/comments
Read quality control *(FASTP [89])*	Trimmomatic [94]	✓	×	(✓)	• Missing implementation to perform special quality control feature (i.e., polyG trimming)
					• Invalid description of workflow differences:
					- ChatGPT stated that polyG trimming is not available in Trimmomatic, but it actually is; however, extra implementation for data adaptation is needed
					- Differences of the generated output files not described accurately
	Cutadapt [95]	×	×	(✓)	• Wrong program call: syntactically incorrect specification of 2 parameters
					• Missing provision of valid adapter sequences
					• Invalid description of differences of the workflow script:
					- ChatGPT stated that polyG trimming is not available in Cutadapt, but it actually is
					- Differences of the generated output files not described accurately
Genome indexing and alignment *(STAR [90])*	HISAT2 [93]	×	×	×	• Invalid definition and linkage of input and output between genome index generation and alignment
					• Syntactically incorrect call of the alignment process
					• Does not take parameter *strandedness* of the input data into account
					• Output files are not generated correctly
	Bowtie [91]	×	×	✓	• Invalid definition and linkage of input and output between genome index generation and alignment
					• Wrong output definition of genome indexing task

To sum up, the study’s results indicate that modifying workflow scripts poses considerable challenges for ChatGPT as it requires a detailed understanding of the tool’s idiosyncrasy, the exact computations they perform, and the data formats they use.

## Study III: Workflow Extension

In the third study, we investigate the capabilities of ChatGPT in extending a scientific workflow given a partial script. As discussed in the motivation for Study II (see Study II: Workflow Modification), users often reuse parts of existing workflows from the research community and adapt them to the research question at hand by enhancing the pipeline with additional analyses and computational steps [6]. Moreover, data analysis projects are often exploratory processes, and computation pipelines are incrementally adapted and extended based on executions and findings from previous versions of the workflow (e.g., to include additional data correctness checks, add more differentiated result evaluations, and provide advanced result visualizations) [96]. In our study, we simulate this incremental exploration process by taking an existing workflow and removing *n* steps at the end of it. We then request ChatGPT to (i) enumerate the necessary steps to accomplish the original research goal and (ii) regenerate the next step using the tool of the original pipeline or by giving a verbal description of the task. For this study, we select 1 workflow from each research domain for investigation: *WF2-RS-Star* for biomedicine and *WF4-Grasslands* for Earth observation. The 2 workflows were chosen because they offer different implementation characteristics; that is, *WF2-RS-Star* leverages almost exclusively external tools, whereas *Grasslands* relies more strongly on specially implemented R and Python scripts. Moreover, Study I (see Study I: Workflow Comprehension) already showed notable result differences of ChatGPT for both workflows. By choosing these 2 specific workflows, we aim to encompass a possibly broad spectrum of performance variations. We test ChatGPT’s workflow extension capabilities in 3 scenarios: for *WF2-RS-Star*, we remove the last step, *transcript quantification*, as well as the last 2 steps, *transcript quantification* and *format conversion*, forming 2 extension scenarios. In the case of *WF4-Grasslands*, we remove all steps at the tail of the workflow, including *autoregressive trend analysis* (see schema in Supplementary Material (D)).

Table 5  illustrates the prompts developed for this purpose. This study uses slightly different prompts (see P3_2a and P3_2b) reflecting the different workflow types (i.e., tool vs. script based). For the latter, we include additional instructions to (i) specify the programming language of the script and (ii) ask the domain expert for a verbal description of the computational steps to be implemented. See Supplementary Material (E) for the verbal description provided by the Earth observation expert. The questionnaire for evaluating the generated outputs consisting of 7 items can be found in the article’s Supplementary Material (C).

### Results

The results are presented in the following according to the 2 subcategories of the prompts: workflow exploration and extension.

#### Workflow exploration

For describing further computational steps necessary to accomplish a specific research goal given a partial workflow, ChatGPT showed mixed results. The LLM provides a correct list of suitable steps in 2 of the 3 scenarios. Also, the tools and methods for implementing the steps suggested by ChatGPT were valid. However, both domain experts criticize that the specifications for the necessary steps and the proposed tools tend to be rather generic and generalized. For instance, for extending the *WF4-Grasslands* workflow, the Earth observation expert commented:

*Overall, the proposed workflow is very generic and does not provide a clear roadmap for the analyses. It also proposes to use very simplistic and often imperfect approaches*.

Overall, the results confirm the findings from the 2 previous studies that ChatGPT shows weaknesses in more exploratory tasks.

#### Workflow extension

Using the prompts P3_2a and P3_2b (see Table 5), we request ChatGPT to reconstruct the last removed computational step in each extension scenario. Table 6 summarizes the results achieved. Like the results from Study II, ChatGPT shows considerable weaknesses in the automatic extension of workflows. None of the generated workflow scripts was executable without the intervention of the domain expert. A clear difference is revealed when comparing the 2 domains, biomedicine and Earth observation. In the former case, the generated workflow scripts are (at least) of such a quality that the domain expert could successfully correct them within 20 minutes. In the generated scripts, mainly syntactical errors occurred (e.g., incorrect usage of variable identifiers, incomplete input definitions, or missing specification of parameters), which could be easily corrected. However, the calls to the respective programs to perform the 2 tasks were correct.

**Table 5:** Overview of the used prompts to investigate ChatGPT’s capabilities in extending a given partial workflow (Study III). We distinguish 2 types of prompts: workflow exploration and workflow extension. For the latter, we developed 2 variants specially designed for tool-based (P3_2a) and script-based (P3_2b) workflows.

ID	Category	Prompt
P3_1	Workflow exploration	The following text contains a *[domain]* workflow written in Nextflow: *[workflow-description]*.
		The workflow should be used to *[overall-goal]*. Which steps are missing in order to perform *[overall-goal]*? Please specify only the absolutely necessary steps. For each step name up to three *[domain]* tools that can be used to perform the task.
P3_2a	Workflow extension	The following text contains a *[domain]* workflow written in Nextflow: *[workflow-description]*.
		Please extend to the given workflow to include one further step which *[step-description]* using *[tool]*. Please specify the new process description in a file at *[file-name]*. Please use version 2 of the Nextflow workflow language. The new process should take the output of *[predecessor-step]* as input.
P3_2b	Workflow extension	The following text contains a *[domain]* workflow written in Nextflow: *[workflow-description]*.
		Please extend to the given workflow to include one further task which performs *[step]* using an *[programming-language]* script. For this, please generate an *[programming-language]* script, stored in *[script-file-name]*, which performs the following computations: *[verbal-task-description]*.
		Next to the *[programming-language]* script generate the Nextflow process description in a file named *[process-file-name]* and the updated workflow. Please use version 2 of the Nextflow workflow language. The new process should take the output of *[predecessor-step]* as input.

**Table 6:** Overview of the results of the workflow extension use case in which we provide ChatGPT a partial workflow and request the LLM to extend it by 1 further computational step. For each investigated use case, we highlight (✓ = yes, × = no) whether the generated workflow script could be executed (Exec.), whether it is semantically valid (Val.), and whether it could be fixed within 20 minutes (Fix). For the latter, (✓) indicates cases where the script could be fixed to be executable but not entirely semantically correct. Moreover, we provide excerpts from the domain expert’s comments.

Workflow	Task/Tool	Exec.	Val.	Fix	Problems/comments
*WF2-RS-Star*	Transcript quantification/*Cufflinks* [99]	×	×	✓	• Syntax errors: process definition for CUFFLINKS declares 1 input channel but 2 were specified
					• Input tuple does not match input set cardinality declared by process definition
					• Wrong variable name: sorted_bam (wrong) instead of sample_bam
					
	Format conversion/*SAMtools*	×	×	✓	• Syntactical errors: usage of wrong variable name (sample_sam)
					• Incorrect syntax for connecting the new task to the previous one SAMTOOLS(STAR_ALIGN.sample_sam) (wrong) vs. SAMTOOLS(STAR_ALIGN.out.sample_sam) (correct)
					
	[100]				
*WF4- Grasslands*	AR analysis/*R script*	×	×	×	• Input to the R script is a path to a directory, not a TIFF file
					• Incorrect use of remotePARTS library: there is no function called *autoTrend* in this package
					• Calculation needs to be parallelized (as specified in the request)
					• Computation should be implemented for 4 types of inputs: GV, NPV, SOIL, and SHADE
					• Desired outputs from the AR model need to be retrieved and written out (missing)
					• Script declares a Conda environment (Python), not R environment

In contrast, the generated extension for *WF4-Grasslands* was of considerably lower quality. In this case, several syntactic and semantic errors occurred (e.g., the script uses a non- library function, no parallelization code is included, and not all requested computations are performed). In this state, the domain expert could not resolve the large number of problems within 20 minutes. However, when interpreting these results, one must remember that the task in this scenario is also significantly more difficult. Instead of a short task description and specification of a tool to be used, ChatGPT has to design and generate the source code for a complex data analysis procedure containing multiple substeps.

## Discussion

We conducted 3 studies to investigate the capabilities of using ChatGPT for comprehending, modifying, and extending scientific workflows. We discuss our methodology and the results in the following.

### Comprehending scientific workflows

Study I was designed to answer **RQ1** by evaluating ChatGPT’s performance in comprehending existing workflows. The domain experts assessed that ChatGPT is good at this task while showing slight differences between the investigated research domains. In particular, the explanations for workflow *WF4-Grasslands* revealed considerable performance drops. Unlike the other workflows investigated, this one uses multiple proprietary R and Python scripts instead of leveraging external tools for assembling data-processing pipelines. The lack of standardized tools makes workflow comprehension more challenging since ChatGPT has to interpret complex processing logic and has fewer possibilities to leverage static information, like the description of the general purpose of an established bioinformatics tool, seen through its training while generating the response. In addition, code quality and its readability may strongly influence the results for workflows containing proprietary scripts. For instance, one major problem while explaining *WF4-Grasslands* in Study I was the misinterpretation of the abbreviation “*fnf*” as “*fraction of non-forest*” instead of “*fold and fill*.” Such customized and ambiguous terms challenge LLMs and reduce their applicability.

In an ablation experiment, we replaced all abbreviations in WF4 with their complete form and regenerated the explanations of prompt P1_1, requesting the overall purpose of the workflow. Having access to the full forms in the script, ChatGPT’s output was much better, even if the exact goal of the workflow was still only met on a rather abstract level:

*aimed at extracting detailed information about land cover dynamics, vegetation phenology, and environmental changes in a specific region using satellite data*.

We also did a reverse experiment using *WF2-RS-Star* by replacing all full-form task names with abbreviations in the workflow script (but keeping the name of the tools called fixed). In this case, the results did not change considerably, highlighting the stronger robustness of ChatGPT against such changes.

### Modifying scientific workflows

We answer **RQ2** in Study II by evaluating the modification performance of ChatGPT. To this end, we requested the LLM to substitute the leveraged tools for 2 computational tasks, read quality control and reference genome alignment, in the biomedical workflow *WF2-RS-Star*. The study results suggest that ChatGPT can effectively explore and explain alternative tools in the field, possibly shortening the time the experts spend searching for suitable replacements on the web. In contrast, the results also indicate that ChatGPT rather poorly supports the generation of workflow scripts for using these alternative tools. In only 1 scenario (i.e., substituting *FASTP* with *Trimmomatic*), the produced script could be run without syntactical errors, and in one other scenario (i.e., replacing STAR with Bowtie), the script could be fixed within 20 minutes to be syntactically and semantically valid. In the used version of ChatGPT and the selected setup, an increase in efficiency cannot be recorded or anticipated, highlighting the need for further research efforts. However, when interpreting the results, it is essential to remember that ChatGPT is a general-purpose LLM rather focusing on human language. A potential option for improvement could be testing generative models more strongly adapted to programming code, such as GitHub Copilot or Code Llama [19]. Moreover, alternative prompting strategies, which adapt the workflow iteratively, could help avoid errors (e.g., when linking the source code of existing tasks with the new task descriptions). We refer to the discussion of other prompting solutions in the Limitations and Future Work section. We emphasize again that workflows and the development of tools in bioinformatics, particularly in the field of genomic analyses, are based on a more extended history and have established more robust and more widely used software than other scientific fields. Accordingly, it is reasonable to infer that the outcomes, such as for suggesting alternative tools, reflect an upper boundary of quality, suggesting that encountering difficulties is more likely in less explored application areas due to insufficient data availability.

### Extending scientific workflows

Finally, we investigate **RQ3** by conducting Study III. To this end, we requested ChatGPT to extend an existing (partially given) workflow to achieve specific goals. The study results confirm the findings from the 2 previous studies and emphasize ChatGPT’s difficulties in solving more complex and exploratory problems. In this case, explaining the necessary steps to answer the given research questions and the generation of the workflow script for the next step offered (partly) severe issues. Similar to the results of Study I, the picture is mixed regarding the different research domains, Earth observation and bioinformatics. For the latter, the generated scripts form a relatively good basis for the implementation, having only (minor) syntactical issues that the expert could quickly fix. In contrast, in the case of Earth observation, the script quality was considerably worse, hindering a fast correction by the expert. These results imply that efficient user support is possible for pipelines mainly leveraging external tools. However, further research is necessary to investigate user-support strategies for workflows applying specially implemented analysis scripts. Specifically, human-in-the-loop approaches that involve human experts more closely in the code generation process could also be helpful here (see Limitations and Future Work section).

### Scientific workflows in LLM training data

LLMs are trained on large amounts of textual data from the web, including programming code and workflow scripts [43]. Therefore, it is crucial to consider whether and to what extent an LLM was already able to access the workflow scripts of our study during its pretraining. According to public information [97], ChatGPT was trained on data gathered until September 2021, meaning that initial versions of 2 of the 5 tested workflows (i.e., *WF3-FORCE2NXF-Rangeland* and *WF5-Force*) could have been part of the LLM’s training routine. However, the specific training dataset used for ChatGPT is not accessible to the public, preventing a conclusive assessment. To attain a more precise estimate of the potential number of workflow scripts within the training data in general, we initiated searches for scientific workflow repositories on GitHub. We leverage the repository search engine of the website [98] and use the names of 4 widely used workflow management systems (i.e., Apache Airflow, Nextflow, Snakemake, and Taverna) as a query term. We filter all repositories with creation data less than 1 September 2021 from the query results. Of course, the results must be interpreted carefully since not every repository containing the name has to deal with scientific workflows, even if the names are very peculiar. Detailed statistics from our search results can be found in this article’s Supplementary Material (F). As of September 2021, there were between 352 and 1,900 repositories containing one of the workflow system names in their description. Moreover, the results highlight the increasing popularity of workflows since, for all systems except for Taverna, the number of repositories has almost doubled over the past 2 years. We also checked the number of Nextflow pipelines available in nf-core. As of September 2021, 35 pipelines were published, and 19 were under development [27]. Today, nf-core hosts 55 published pipelines and 33 in development. In summary, we can hypothesize from these results that ChatGPT can likely rely only on a relatively small base of workflow scripts during its training compared to classical programming code (e.g., GitHub currently hosts over 3.9 million Java and over 2.2 million Python repositories, which we determined by using GitHub repository search and the search queries “*language:Java*” and “*language:Python*,” making user support for workflow design and implementation particularly challenging).

### Prompt design challenges

While creating prompts for the studies, we identified several challenges and issues that arose while interacting with ChatGPT.

#### Representation of workflows

For the representation of the workflow scripts, there is no straightforward option on how to include them in a prompt. The workflow descriptions are often spread over several files containing sub-workflows and task descriptions. In our approach, we first specify the main workflow and then all sub-workflows and task descriptions in order of occurrence. However, there might be other, more efficient prompt solutions (with respect to the generative language model). Furthermore, the workflow scripts might exceed the maximum allowed input length of the language model (e.g., ChatGPT variants allow only for 4,000 to 16,000 words/tokens [97] in the input sequence). In particular, workflows heavily relying on specially implemented scripts having hundreds of code lines will face this issue.

#### Loss of focus

Some of the prompts are very long due to the specification of the entire workflow script, which challenges ChatGPT to maintain focus. Adding additional instructions to the prompt helped to avoid or reduce this phenomenon; for example, for the explanation use case (Study I), we added to the prompt *“Don’t explain nextflow concepts”* (see P1_1 and P1_2 in Table 2) and *“Don’t explain the workflow itself”* (P1_3) to prevent ChatGPT generating outputs describing features of the workflow management system or the complete workflow when requesting input data specification.

#### Technological details

In some cases, adaptation to technological details of the specified workflows was necessary. For example, the Nextflow system offers 2 language versions for describing processing pipelines. The Nextflow workflows in our study all used the new version of the language. However, when extending workflows in Study III, we had to specify the desired version (see P3_2 in Table 5) to get the correct output. This observation is surprising since the partially given workflow is already in the respective version. Interestingly, this was only necessary for the workflow extension but not for their modification (P2_4 in Table 3) in which the phenomena did not occur.

In summary, the efficient and effective formation of prompts offers a wide range of possible solutions. In our study, we identified initial clues and difficulties, but further research is needed to detect further potential for improving the interaction between domain experts and ChatGPT and generative LLMs in general.

### Limitations and future work

In the following, we highlight the limitations of this work that merit further research.

#### Study design

In each of our 3 studies, we created and provided the prompts for testing ChatGPT’s capabilities concerning the different use cases, and the domain experts only evaluated the outputs of ChatGPT, leading to a rather indirect interaction between the domain scientist and the LLM. An alternative design for the study would be to have the experts interact directly with ChatGPT by developing and refining the prompts independently. In addition to assessing the capabilities of ChatGPT, this would have the advantage of gaining initial insights into interaction forms and patterns of the different experts with ChatGPT. Moreover, this would allow for improved customization of the prompts to the particular research domain and the idiosyncratic properties and characteristics of each workflow. Extended optimization of the prompting strategy by the domain scientist could lead to better results but reduce potential time savings in solving the actual task. Our study design was motivated by the fact that the experts had strongly limited time budgets for the study. For example, even for evaluating ChatGPT’s outputs in Study I, the experts already needed up to 3 hours to accurately check the generated explanations. A study design that envisages direct interaction involves high efforts in terms of introduction and explanation to ChatGPT and prompting strategies for the domain scientists, thus limiting the scope of research questions that can be investigated. In addition, the selected study design has the advantage of using the same prompts for the different domains, which contributes to better comparability of the results and eliminates the influence of differences for individual prompt differences.

In our study, we focused solely on ChatGPT as generative language model. However, there are many other general-purpose models available (e.g., PaLM-2 [45], Gemini [101], or Llama-2 [19]) as well as models more specially designed for programming tasks (e.g., GitHub Copilot, Code Llama [102], or OpenAI Codex [103]) publicly available and worth investigating. Furthermore, recent research showed that placebo effects can undermine the validity of study results when user expectations are altered through the presence of an artificial intelligence [104–106] (i.e., LLM) or a novel superior technology [107, 108] that improves user capabilities. Our studies only highlight the results of ChatGPT in the version used (GPT-3.5) but do not claim generalizability for other LLMs. In future work and in the case of using LLMs, placebo conditions must be included to avoid findings that are not a result of increased user expectations toward the capabilities of ChatGPT.

#### Proprietary and closed-source LLMs

While ChatGPT and other proprietary LLMs offer remarkable natural language understanding and generation capabilities, they have inherent limitations that can hinder their utility in scientific workflow development. One significant limitation is their lack of transparency in their underlying algorithms and training data, restricting results’ direct reproducibility and interpretability. Users typically interact with these models through an application programming interface or web application provided by the company or organization that developed it rather than having direct access to the underlying code or data, which often makes it impossible to trace any changes to the model and, thus, to the results achieved. Moreover, the closed nature of these models restricts researchers’ ability to customize or fine-tune them for specific tasks, limiting their adaptability to diverse research domains. An alternative is using freely available, open-source LLMs, like BLOOM [20] or LLaMA [19], where users have more control over model changes. Nonetheless, the data basis and training procedure are often not fully transparent, even with these models. Moreover, it is essential to note that running such models demands substantial computational resources and the corresponding technical expertise.

#### Prompting strategy

Next to other models, the prompts used in our studies also constitute a limiting factor. We cannot exclude the possibility that other prompts, using a different structure or wordings, may achieve better results for the investigated use cases. Moreover, in our approach, we used only the workflow description’s source code, without comments, to form the prompt. There are several strategies for enriching the prompt with additional context information, which can lead to improved results. For instance, including available documentation artifacts of the workflow, for example, descriptive texts in the source code repository or the publication introducing (parts of) the workflow, may ease the processing of the text. Similarly, utilizing descriptions or manuals for the tools employed could also contribute to better results.

In addition, the prompts for individual aspects of the workflows could be revised to obtain more differentiated results. For instance, the prompts for exploring alternative tools (see P2_1 and P2_2 in Table 3) could be extended to request further semi-structured information, such as the programming language used, software license, release date, and development history, guiding the tool selection process. Information on the development history, for example, could help domain experts differentiate between well-established, trusted tools and new developments. This would additionally enhance result interpretation since there might be limited information available for recent advancements, potentially leading to their (yet) insufficient representation in the LLM.

Furthermore, our approach is based on a linear prompt execution without feedback or revision opportunities. Iterative prompting strategies, such as chain-of-thought [109] or graph-of-thoughts prompting [110], self-debugging [111] or self-adapting approaches [112], can potentially enhance results. These strategies offer dynamic interaction approaches with LLMs, enabling continuous improvement and adaptation of responses through successive refinements. This facilitates, for example, the correction of errors encountered during workflow modification and extension, such as syntax errors in tool invocation, data format mismatches, or invalid task linking, by utilizing the LLM output as input for the subsequent iteration. In addition to other technical possibilities of the LLM conversation, prompt customization and adaptation by human experts offer strong potential for improvement [113, 114]. Using such human-in-the-loop approaches allows for explicitly addressing the observed problems, especially in workflow modification and expansion, in a semi-automatic process.

#### Stochastic generation

In addition to alternative prompting strategies, it should be emphasized that the generated texts are subject to stochastic sampling processes, which can lead to deviations even when reusing the exact same prompts multiple times. In most LLMs, the influence of this phenomenon can be managed using the *temperature* hyperparameter, which regulates how the model samples the next word by adjusting the probability distribution. Higher temperature values soften the distribution, leading to more diverse and creative outputs, allowing the model to explore a broader range of possibilities. On the other hand, a lower temperature tends to produce more conservative and predictable outputs, as the model is more likely to choose tokens with higher probabilities. Thus, the parameter serves as a knob to adjust the balance between exploration (generating diverse outputs) and exploitation (producing more likely outputs). An extensive investigation of the parameter’s influence represents an interesting future research question.

#### Limited number of domain experts

In the context of our studies, only 4 domain experts evaluated the outputs of ChatGPT. In some cases, generated explanations were assessed by one person only (e.g., Study II). This low number of experts limits the validity and generalizability of the results and offers the risk of subjective bias. However, recruitment for such studies is difficult because the number of potential participants is small and they often have strongly limited time budgets, making study design challenging. Please note that for experts in the field, even “just” familiarizing themselves with an unfamiliar workflow is a challenging and time-consuming endeavor.

#### Investigated domains and selected workflows

Our study explores real-world workflows from the 2 domains, bioinformatics and Earth observation. Of course, these only represent part of the full range of workflows in the natural sciences. It constitutes an exciting follow-up research question: how suitable ChatGPT and other generative LLMs are in other research contexts, such as climate research [33] and astronomy [32], and whether it is possible to identify categories or groups of domains that are particularly well (or poorly) supported. Furthermore, we examined only 2 workflow systems, Nextflow and Apache Airflow, leaving other alternatives, such as Snakemake, Taverna, and Pegasus, for future work. Investigating other workflow systems is especially interesting because they specify different languages with different complexity levels and support features for designing analysis pipelines. For example, the language of Apache Airflow, which prioritizes flexibility and extensibility through its integration with the general-purpose programming language Python, expands the potential output scope compared to more deterministic languages such as the Common Workflow Language [115, 116]. Consequently, this increased variability likely poses considerable challenges for LLMs. For this, a thorough analysis of how the idiosyncrasy of the leveraged workflow language influences the outcomes produced by LLMs would be beneficial in offering guidance to practitioners.

#### Explored use cases

This work focused on comprehending, modifying, and extending workflows with ChatGPT. These use cases represent only a partial scope of user support opportunities, and it is worth considering and evaluating other use cases. For instance, migrating workflows implemented in legacy workflow management systems to more recent ones (e.g., transforming Taverna [86] scripts to Snakemake or Nextflow) or adapting them to different infrastructure stacks poses an interesting research question. Moreover, user support in workflow debugging, error identification, or optimization, as done in classical programming [62], would be a valuable contribution to research scientists.

## Conclusion

The significance of large-scale data analysis workflows in advancing research in the natural sciences is growing steadily. Developers of such workflows, primarily researchers from diverse scientific fields, are challenged with the increasing complexity and scale of their analyses, which involve (next to their domain knowledge) working with different frameworks, tools, programming languages, and infrastructure stacks. Although a few tools for creating and maintaining workflows are available, improving user efficiency remains an open research area. In this work, we contribute to this situation by evaluating the suitability of ChatGPT for comprehending, modifying, and extending scientific workflows. In 3 user studies with 4 researchers from different scientific domains, we evaluated the correctness of ChatGPT regarding explainability, exchange of software components, and extension when providing real-world scientific workflow descriptions. Our results show a high accuracy for comprehending and explaining scientific workflows while achieving a reduced performance for modifying and extending workflow descriptions. These findings clearly illustrate the need for further research in this area.

## Additional Files

**Supplementary Material**. Complete questionnaires for user studies I–III, schema of workflow *WF4-Grasslands*, verbal task description for user study III, and statistics of the GitHub search results.

**Supplementary Fig. S1**. Overview of the Earth observation workflow *WF4-Grasslands* developed by one of the domain experts. The workflow aims at understanding differences in long-term changes (1984–2022) in ground cover fractions specific to European grasslands depending on the definition of endmembers (i.e., unique spectral signatures of a specific material or ground cover) approximating these fractions. The figure highlights the conceptual schema and dataflow of the workflow.

**Supplementary Table S1**. Feedback form for the first user study that investigates the capabilities of ChatGPT to capture the content of a workflow description. For each item, we added a comment field to report issues and errors in the generated explanations if the domain expert does not fully apply the content.

**Supplementary Table S2**. Feedback form for the second user study that investigates the capabilities of ChatGPT in exchanging the used tools in a scientific workflow. For each item, we added a comment field to report issues and errors in the generated explanations if the domain expert doesn’t fully apply with the content.

**Supplementary Table S3**. Feedback form for the third user study that investigates the capabilities of ChatGPT to extend a given (partial) workflow script. For each item, we added a comment field to report issues and errors in the generated explanations if the domain expert doesn’t fully apply with the content.

**Supplementary Table S4**. Statistics of the search results for 4 different scientific workflow systems using the GitHub search engine. For each system, we use the system name as search term and restrict the result repositories to be created before the date give by the column (group).

## Abbreviations

LLM: large language model.

## Supplementary Material

supplementary_material

GIGA_D_24_00009_Original_Submission

GIGA_D_24_00009_Revision_1

GIGA_D_24_00009_Revision_2

Response_to_Reviewer_Comments_Original_Submission

Response_to_Reviewer_Comments_Revision_1

Reviewer_1_Report_Original_Submission Tazro Ohta -- 2/8/2024

Reviewer_1_Report_Revision_1 Tazro Ohta -- 3/27/2024

Reviewer_2_Report_Original_Submission Konstantinos Krampis, PhD -- 2/12/2024

## Data Availability

The source code of all investigated workflows is available via [74–78]. We provide the complete LLM chat outputs as supplementary material to this article via the *GigaScience* database, GigaDB [71].
