# Supplementary material for: A qualitative assessment of using ChatGPT as large language model for scientific workflow development
Source: Gigascience. 2024 Jun 19;13:giae030. doi: 10.1093/gigascience/giae030 (PMC11186067; doi:10.1093/gigascience/giae030)
Supplement: supplementary_material [file supplementary_material.pdf]

Supplementary Material A: Questionnaire Study I

Table A.1. Feedback form for the first user study which investigates the capabilities of ChatGPT to capture the content of a workflow description. For each item, we added a comment field to report issues and errors in the generated explanations if the domain expert does not fully apply the content.

Prompt	ID	Question	Answer options
P1_1	Q1_1	The generated explanation matches the research area of the workflow	5-point Likert scale
	Q1_2	The generated explanation matches the overall aim of the workflow	5-point Likert scale
P1_2	Q1_3	How many of the tasks of the workflow (see task overview below) are contained in the description?	Numerical
	Q1_4	The explanation describes the tasks of the workflow correctly	(1) Don't know<br>(2) None of the tasks is correct<br>(3) Few tasks are correct<br>(4) Most tasks are correct<br>(5) All tasks are correct
	Q1_5	How many of the software programs / tools used in the workflow are mentioned in the explanation?	Numerical
	Q1_6	The explanation of the software programs / tools used is correct	(1) Don't know<br>(2) None of the software programs / tools is correct<br>(3) Few software programs / tools are correct<br>(4) Most software programs / tools are correct<br>(5) All software programs / tools are correct
P1_3	Q1_7	The explanation matches the input data specification of the workflow	5-point Likert scale
P1_4	Q1_8	The generated explanation matches the overall result of the workflow	5-point Likert scale
P1_5	Q1_9	How many of the generated research questions are valid?	Numerical

Supplementary Material B: Questionnaire Study II

Table B.1. Feedback form for the second user study which investigates the capabilities of ChatGPT in exchanging the used tools in a scientific workflow. For each item we added a comment field to report issues and errors in the generated explanations if the domain expert doesn't fully apply with the content.

Prompt	ID	Question	Answer options
P2_1	Q2_1	How many of the 10 alternative tools are valid?	Numerical
P2_2	Q2_2	The selected tools are reasonable alternatives for the task?	5-point Likert scale
	Q2_3	The generated explanation for the first tool highlights the suitability of the tool for the task well	5-point Likert scale
	Q2_4	The generated explanation for second tool highlights the suitability of the tool for the task well	5-point Likert scale
P2_3	Q2_5	The generated explanation helps to understand the methodical differences	5-point Likert scale
	Q2_6	The generated explanation highlights strengthens and weaknesses of *[original-tool]* correctly	5-point Likert scale
	Q2_7	The generated explanation highlights strengthens and weaknesses of *[alternative-tool]* correctly	5-point Likert scale
P2_4	Q2_8	The adaption of the workflow is correct	5-point Likert scale
	Q2_9	The adaption of the task *[task-name]* is correct	5-point Likert scale
	Q2_10	The explanation of the not-supported features by *[alternative-tool]* is correct	5-point Likert scale
	Q2_11	Can the workflow be executed without errors?	Yes/No
	Q2_12	How long did it take to correct the workflow?	Numerical
	Q2_13	What had to be adapted to make the workflow executable?	Free-text

Supplementary Material C: Questionnaire Study III

Table C.1. Feedback form for the third user study which investigates the capabilities of ChatGPT to extend a given (partial) workflow script. For each item we added a comment field to report issues and errors in the generated explanations if the domain expert doesn't fully apply with the content.

Prompt	ID	Question	Answer options
P3_1	Q3_1	Is the list of necessary steps complete?	Yes/No
	Q3_2	How many of the proposed steps are correct? Please give your answer in the form of "x out of y steps".	Free text
	Q3_3	How many of the proposed tools are correct? Please give your answer in the form of "x out of y tools".	Free text
P3_2a/b	Q3_4	The extension of the workflow is correct	5-point Likert scale
	Q3_5	Can the workflow be executed without errors?	Yes/No
	Q3_6	How long did it take to correct the workflow? Enter "not finished" when you set yourself a time limit that has run up.	Free text
	Q3_7	What had to be adapted to make the workflow executable?	Free text

Supplementary Material D: Schema WF4-Grasslands

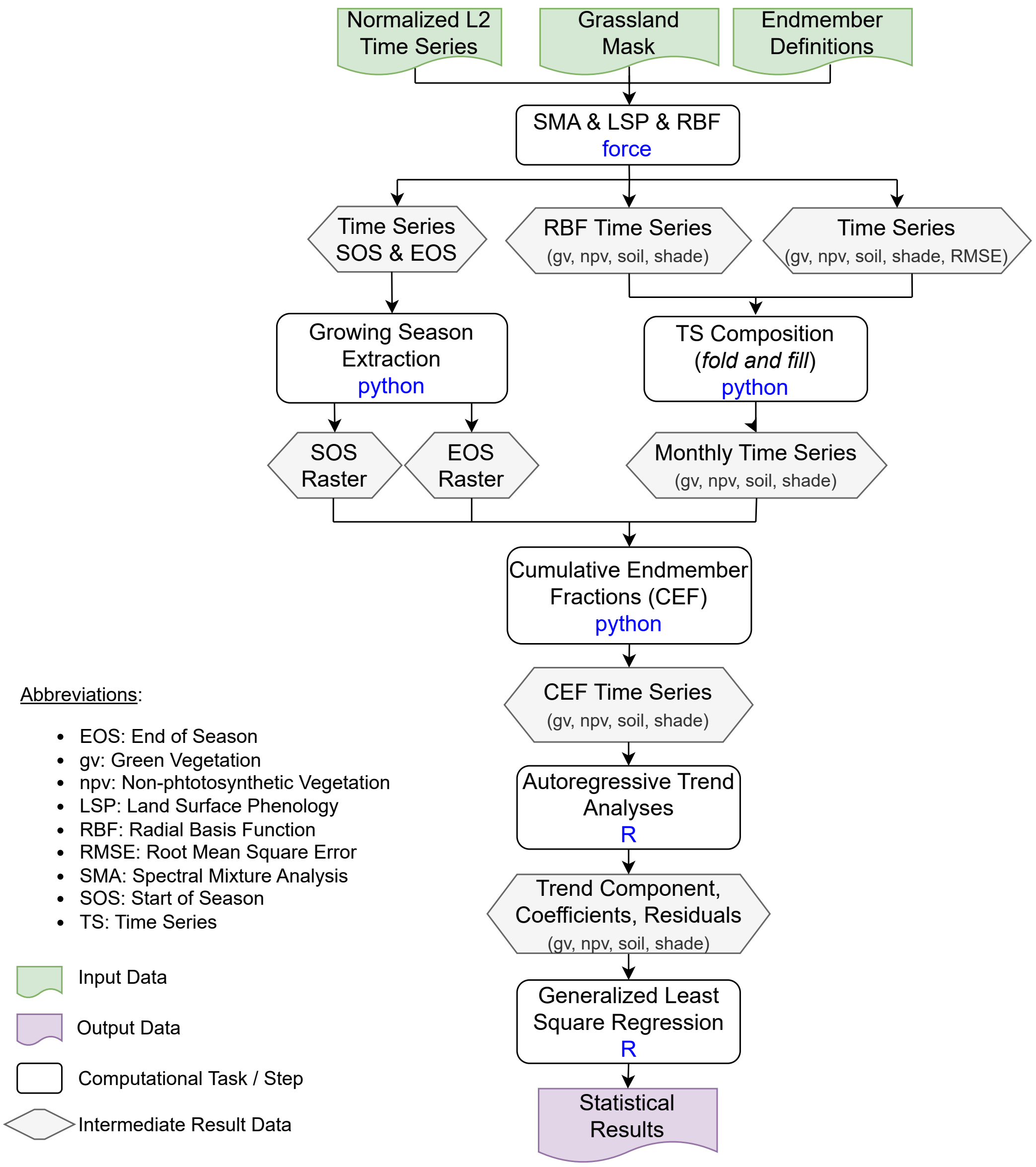

Figure D.1. Overview of the earth observation workflow *WF4-Grasslands* developed by one of the domain experts. The workflow aims at understanding differences in long-term changes (1984-2022) in ground cover fractions specific to European grasslands depending on the definition of endmembers (i.e., unique spectral signatures of a specific material or ground cover) approximating these fractions. The figure highlights the conceptual schema and data flow of the workflow.

Supplementary Material E: Verbal Task Description

In Study III, we use the following task description, provided by the earth observation expert, for extending *WF4-Grasslands* with an autoregressive trend analysis:

- step 1: it reads the data from a multi-band TIFF file into a raster brick as defined with the raster package. The input file needs to be selected based on a location specified by a string parameter passed into the script
- step 2: it converts all 0s in the data brick to 'NA'
- step 3: it fits per-pixel time trend with Auto Regressive trend function from the remotePARTS R package. Each raster in a data brick represents one time step in a time series over which the trend is fitted. The result of the trend fitting should be a multi-band raster in a TIFF format that comprises bands with information on trend's slope, intercept, p-value, temporal auto-correlation, and residuals for each time step.
- The steps 1-3 should be applied repetitively and independently to four raster datasets available for each location, namely: GV, NPV, SOIL, and SHADE.
- The script should be implemented using parallel cluster processing functionality in the raster package and provide control over the number of nodes, and RAM available to the process.

Supplementary Material F: GitHub Search Results

Table F.1. Statistics of the search results for four different scientific workflow systems using the GitHub search engine. For each system we use the system name as search term and restrict the result repositories to be created before the date give by the column (group).

Search Term	< 2021-09-01		< 2023-09-01	
	# Repositories	# Pull requests	# Repositories	# Commits
apache airflow	1,200	27,000	2,600	85,000
nextflow	1,700	5,000	3,200	14,000
snakemake	1,900	5,000	3,400	18,000
taverna	352	244	472	645
