# Supplementary material for: A qualitative assessment of using ChatGPT as large language model for scientific workflow development
Source: Gigascience. 2024 Jun 19;13:giae030. doi: 10.1093/gigascience/giae030 (PMC11186067; doi:10.1093/gigascience/giae030)
Supplement: GIGA_D_24_00009_Revision_2 [file giga_d_24_00009_revision_2.pdf]

GigaScience

A Qualitative Assessment of Using ChatGPT as Large Language Model for Scientific Workflow Development

--Manuscript Draft--

**Manuscript Number:**	GIGA-D-24-00009R2
**Full Title:**	A Qualitative Assessment of Using ChatGPT as Large Language Model for Scientific Workflow Development
**Article Type:**	Research
**Funding Information:**	Deutsche Forschungsgemeinschaft (414984028) — Not applicable
**Abstract:**	Background: Scientific workflow systems are increasingly popular for expressing and executing complex data analysis pipelines over large datasets, as they offer reproducibility, dependability, and scalability of analyses by automatic parallelization on large compute clusters. However, implementing workflows is difficult due to the involvement of many black-box tools and the deep infrastructure stack necessary for their execution. Simultaneously, user-supporting tools are rare, and the number of available examples is much lower than in classical programming languages.<br>Results: To address these challenges, we investigate the efficiency of Large Language Models (LLMs), specifically ChatGPT, to support users when dealing with scientific workflows. We performed three user studies in two scientific domains to evaluate ChatGPT for comprehending, adapting, and extending workflows. Our results indicate that LLMs efficiently interpret workflows but achieve lower performance for exchanging components or purposeful workflow extensions. We characterize their limitations in these challenging scenarios and suggest future research directions.<br>Conclusions: Our results show a high accuracy for comprehending and explaining scientific workflows while achieving a reduced performance for modifying and extending workflow descriptions. These findings clearly illustrate the need for further research in this area.
**Corresponding Author:**	Mario Sänger<br>Humboldt-Universität zu Berlin: Humboldt-Universitat zu Berlin<br>Berlin, GERMANY
**Corresponding Author Secondary Information:**	
**Corresponding Author's Institution:**	Humboldt-Universität zu Berlin: Humboldt-Universitat zu Berlin
**Corresponding Author's Secondary Institution:**	
**First Author:**	Mario Sänger
**First Author Secondary Information:**	
**Order of Authors:**	Mario Sänger
	Ninon De Mecquenem
	Katarzyna Ewa Lewińska
	Vasilis Bountris
	Fabian Lehmann
	Ulf Leser
	Thomas Kosch
**Order of Authors Secondary Information:**	
**Response to Reviewers:**	Dear editor,<br><br>thank you again for giving us the opportunity to publish our research in your journal. We revised the manuscript according to your comments.

Powered by Editorial Manager® and ProduXion Manager® from Aries Systems Corporation

	If you need further information or support, do not hesitate to contact us.<br><br>Best,<br>Mario & Thomas
**Additional Information:**	
**Question**	**Response**
Are you submitting this manuscript to a special series or article collection?	No
**Experimental design and statistics**<br><br>Full details of the experimental design and statistical methods used should be given in the Methods section, as detailed in our Minimum Standards Reporting Checklist. Information essential to interpreting the data presented should be made available in the figure legends.<br><br>Have you included all the information requested in your manuscript?	Yes
**Resources**<br><br>A description of all resources used, including antibodies, cell lines, animals and software tools, with enough information to allow them to be uniquely identified, should be included in the Methods section. Authors are strongly encouraged to cite Research Resource Identifiers (RRIDs) for antibodies, model organisms and tools, where possible.<br><br>Have you included the information requested as detailed in our Minimum Standards Reporting Checklist?	Yes
**Availability of data and materials**<br><br>All datasets and code on which the conclusions of the paper rely must be either included in your submission or deposited in publicly available repositories (where available and ethically appropriate), referencing such data using	Yes

a unique identifier in the references and in the “Availability of Data and Materials” section of your manuscript.<br><br>Have you have met the above requirement as detailed in our Minimum Standards Reporting Checklist?	

Click here to access/download;LaTeX - Main Document (TeX file);manuscript.tex

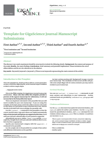

GigaScience, 2024, 1–17

doi: xx.xxxx/xxxx
Manuscript in Preparation
Paper

PAPER

A Qualitative Assessment of Using ChatGPT as Large Language Model for Scientific Workflow Development

Mario Sänger [0000-0002-2950-2587][1,*], Ninon De Mecquenem [0000-0003-3052-6129][1], Katarzyna Ewa Lewińska [0000-0001-6560-1235][2,3], Vasilis Bountris [0000-0002-8682-7302][1], Fabian Lehmann [0000-0003-0520-0792][1], Ulf Leser [0000-0003-2166-9582][1] and Thomas Kosch [0000-0001-6300-9035][1,*]

[1]Department of Computer Science, Humboldt-Universität zu Berlin, 10099 Berlin, Germany and [2]Department of Geography, Humboldt-Universität zu Berlin, 10099 Berlin, Germany and [3]Department of Forest and Wildlife Ecology, University of Wisconsin-Madison, WI 53706, USA

[*]Corresponding authors:{saengema,thomas.kosch}@informatik.hu-berlin.de

Abstract

Background: Scientific workflow systems are increasingly popular for expressing and executing complex data analysis pipelines over large datasets, as they offer reproducibility, dependability, and scalability of analyses by automatic parallelization on large compute clusters. However, implementing workflows is difficult due to the involvement of many black-box tools and the deep infrastructure stack necessary for their execution. Simultaneously, user-supporting tools are rare, and the number of available examples is much lower than in classical programming languages.
Results: To address these challenges, we investigate the efficiency of Large Language Models (LLMs), specifically ChatGPT, to support users when dealing with scientific workflows. We performed three user studies in two scientific domains to evaluate ChatGPT for comprehending, adapting, and extending workflows. Our results indicate that LLMs efficiently interpret workflows but achieve lower performance for exchanging components or purposeful workflow extensions. We characterize their limitations in these challenging scenarios and suggest future research directions.
Conclusions: Our results show a high accuracy for comprehending and explaining scientific workflows while achieving a reduced performance for modifying and extending workflow descriptions. These findings clearly illustrate the need for further research in this area.

Key words: Large Language Models; Scientific Workflows; User Support; ChatGPT

Introduction

Large-scale data analysis pipelines (also known as scientific workflows) are crucial in driving research advances for natural sciences [1]. They are pivotal in accelerating large and complex data analysis on distributed infrastructures and offer essential features, such as reproducibility and dependability [2]. In bioinformatics, for instance, scientific workflows are analyzing the terabyte-large data sets produced by modern DNA or RNA sequencing machines in a wide variety of experiments [3], thereby aiding in building a comprehensive understanding of biological processes and human diseases. Bioinformatics workflows typically include many individual computational steps, such as data pre-processing, extensive quality control, aggregation of raw sequencing data into consensus sequences, machine-learning-based tasks for classification and clustering, statistical assessments, and result visualization. Each

Compiled on: May 11, 2024.
Draft manuscript prepared by the author.

Key Points

- We explore Large Language Models (LLMs) to support users who develop scientific workflows
- We are the first to conduct user studies involving domain experts
- We conduct three studies to assess LLMs in scientific workflow comprehension, adaptation, and extension
- Our results indicate that LLMs efficiently interpret workflows
- Our results show room for improvement regarding component adaptation and workflow extension

step is carried out by a specific program, typically not written by the workflow developer but exchanged within a worldwide community of researchers [4]. Execution of a workflow on a distributed infrastructure, in principle, is taken care of by a workflow engine; however, the idiosyncrasies of the different infrastructures (e.g., file system, number and features of compute nodes, applied resource manager, and scheduler) often require workflow users to tune their scripts individually for every new system [5].

However, typical developers of workflows are researchers from heterogeneous scientific fields who possess expertise in their respective domains but often lack in-depth knowledge in software development or distributed computing. They often encounter difficulties understanding the complex implementations of exchanged codes and the deep infrastructure stack necessary for their distributed execution. This situation challenges efficient workflow implementation, slows down or hinders data exploration and scientific innovation processes [6]. Consequently, low human productivity is a significant bottleneck in the creation, adaption, and interpretation of scientific workflows [7].

Parallel to this, there is a well-established field in human-computer interaction focusing on assisting end-user programmers and software development, as highlighted by previous work [8, 9, 10, 11], to reduce the perceived cognitive workload and improve the overall programmer performance [12]. Research in this field includes programming-by-demonstration [13, 14], visual programming [15, 16], and natural language instructions [17]. Recent work in this area particularly investigated prospects of general-purpose Large Language Models (LLMs), such as ChatGPT [18], LLaMA [19] and Bloom[20], for supporting end-user-programming [21, 22, 23] and software development in general [24, 25]. For instance, Bimbatti et al. [21] explore using ChatGPT to enhance natural language understanding within an end-user development environment, assisting non-expert users in developing programs for collaborative robots. Moreover, White et al. [24] introduce prompt design techniques for automating typical software engineering tasks, including ensuring code independence from third-party libraries and generating an API specification from a list of requirements. Surameery et al. [25] evaluate LLMs for supporting code debugging. However, results from such studies, which focus on standard programming languages, cannot easily be transferred to workflow systems. Workflow scripts mostly call external tools with agnostic names and have little recognizable control structures or protected keywords. Publicly available examples are scarce; for instance, the community repository of the popular workflow system Nextflow [26] currently offers only 55 released workflows [27]. Furthermore, workflows can only be understood when the distributed system underlying their execution is considered, creating dependencies much different than usual programs. Moreover, studies investigating how LLMs support users in data science - the field in which workflows are applied extensively - do not address the unique characteristics of scientific workflows either and are limited to theoretical considerations [28, 29]. Practical studies, especially those involving real users, are badly missing.

In this work, we address these shortcomings by describing three user-studies in two different scientific fields (biomedicine and Earth observation) that evaluate the suitability of ChatGPT for comprehending, modifying, and extending scientific workflows. Specifically, we evaluate the correctness of ChatGPT regarding explainability, exchange of software components, and extension when providing real-world scientific workflow scripts. Our results show a high accuracy for comprehending and explaining scientific workflows but reduced performance for modifying and extending workflow scripts. The domain experts positively assessed the explainability in qualitative inquiries, emphasizing the time-saving capabilities of using LLMs while engineering existing workflows. Overall, our work indicates that general-purpose LLMs have the potential to improve human performance when analyzing complex scientific workflows.

Background

Previous research investigated how related domains, such as programming, can be augmented using interactive technologies [11, 30]. In contrast to programming, where applications use a single programming language and are often executed on a single system, scientific workflows combine multiple software artifacts on distributed stacks for advanced data processing. We ground the reader by providing a literature review about scientific workflows and introducing large language models, including their utility to facilitate the creation of software artifacts.

Scientific Workflows

Scientific workflows are widely used by diverse research communities, such as biomedicine [31], astronomy [32], climatology [33], and Earth observation [34] to manage the dataflow and distributed execution of complex analyses, simulations, and experiments. A scientific workflow comprises a series of interconnected computational steps, often with diverse patterns of dependencies, that define how to process and analyze data to reach a particular research objective. Scientific workflows can be regarded as directed acyclic graphs in which the nodes represent computational tasks or operations and edges model dependencies or dataflow between these tasks. An edge from one node to another signifies that the output of the first task is used as input for the second [35]. For example, Figure 1a illustrates the computational steps, the tools, and the data flow of a bioinformatics workflow for performing differential gene expression analysis. During workflow execution, a single computation step often involves multiple processes, which are typically executed in a distributed fashion on different machines and batches of the input data, resulting in a much more complex execution graph. Consequently, scientific workflows help facilitate the reproducibility and traceability of data analyses by explicitly outlining the steps and parameters involved [36]. Furthermore, they allow for automation, scaling, and optimization of computational processes, which is especially critical in disciplines dealing with large datasets [37]. The increasing importance of scientific workflows for scientific progress has led to a growing interest in developing more user-friendly tools and methods through the research community. Scientific workflow management systems, like Apache Airflow [38], Galaxy [39], Nextflow [40], Pegasus [41], and Snake-

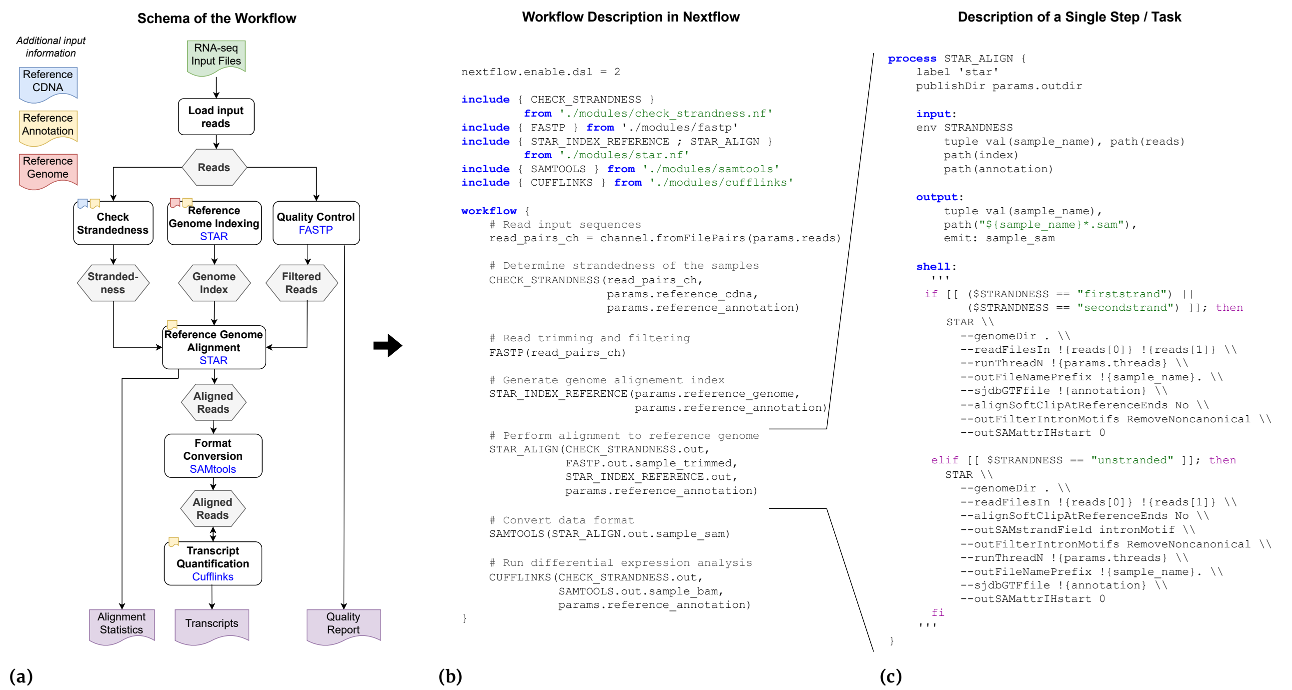

Figure 1. Example bioinformatics workflow for differential gene expression analysis created by a domain expert recruited in our study. The figure highlights the conceptual schema of the workflow (a), its implementation in Nextflow (b), and the implementation of one single step (c), i.e., reference genome alignment using the STAR tool. The workflow comprises six computational steps in total. For each step the used tool is given in blue below the task name.

make [42], are specifically developed to support users in designing and executing scientific workflows in various aspects. Key features of such management systems typically include workflow design and composition, (distributed) workflow execution and scheduling, provenance tracking, recovery and failure handling, and resource management [36]. Figure 1b highlights the implementation of the example workflow as well as a single computational step (see Figure 1c), i.e., reference genome alignment using the STAR toolkit in Nextflow.

Scientific workflows are often reused and adopted for complex data analysis [6]. Most of the time, users of scientific workflows are unaware of the workflow's internal functionality and technical details. Instead, users of scientific workflows represent domain experts, such as mathematicians, physicians, or bioinformatics, who are experts in their respective domains but not necessarily in programming and interpreting scientific workflows. Existing scientific workflows were implemented and maintained by persons other than the domain user. This reduces the direct interaction with scientific workflows to a minimum, where domain experts only hand in the input data and evaluate the output data. Consequently, domain experts using a workflow often do not have the knowledge to modify, extend, or interpret the details of scientific workflows.

Large Language Models

Language Models, such as BERT [43], GPT-3 [44], Bloom [20] and PaLM-2 [45], build the foundation of many recent advancements in natural language processing and understanding. These models have billions of parameters and are generally pre-trained on vast sets of texts from the web and other repositories, enabling them to encode syntactic and semantic relationships in human language. As a consequence, generative language models, such as ChatGPT, LLaMA [19], and LAMDA [46], can produce machine-generated high-quality text that is indistinguishable from human writing. These generative capabilities have empowered these models to assist in diverse (creative) writing tasks and have been utilized to facilitate a wide range of interactive language-based applications within the HCI community [47, 48, 49, 50, 51, 52]. For instance, WordCraft [47] investigate the utilization of LLMs to aid fiction writers in tasks ranging from transforming a text to resemble a "Dickensian" style to providing suggestions to combat writer's block. Their findings indicate that writers found such text-generating models beneficial even when the generated text is not perfect. Petridis et al. [48] introduce AngleKindling, an interactive tool that employs LLMs to support journalists exploring different angles for reporting on a press release. Their study with twelve professional journalists shows that participants found the system considerably more helpful and less mentally demanding than competitor brainstorming tools. Other applications include prototyping support [49], generation of titles and synopses from keywords [52], conversational interactions with mobile user interfaces [50], and conceptional blending [51].

Next to their text writing capabilities, language models are further known to retain commonsense knowledge within their training data, effectively transforming them into accessible knowledge stores that can be seamlessly queried using natural language prompts. For instance, experiments with BERT [43] highlight that the model performance is competitive with traditional information extraction and open-domain question answering. Furthermore, recent studies show the potential of using ChatGPT for knowledge base construction, inspired by the fact that these language models have been pre-trained on vast internet-scale corpora that encompass diverse knowledge domains [53]. However, it is worth noting that LLMs are known to frequently generate hallucinations, which are outputs that, while statistically plausible and seemingly believable, are factually incorrect [54, 55].

Using LLMs to Support Programming

The ability to generate new text and to reconstruct existing information makes LLMs highly appropriate to support users in software development, as programming often requires not only the creation of novel code segments tailored to current requirements and tasks but also depends on the application of established algorithms,

software libraries, and best practices. Accordingly, a large number of papers investigate LLMs specially trained for code generation [56, 57, 58, 59] as well as different approaches leveraging these models to provide interactive programmer support [60, 61, 62, 23]. For instance, Jiang et al. [61] discuss GenLine, a natural language code synthesis tool based on a generative LLM and problem-specific prompts for creating or changing program code. The findings from a user study indicate that the approach can provide valuable support to developers. However, they also encounter several challenges, such as participants finding it difficult to form an accurate mental model of the kinds of requests that the model can reliably translate. Similarly, Vaithilingam et al. [62] conducted a user study with 24 participants evaluating their usage and experiences using the GitHub Copilot [63] code generation model while programming. The authors find that the synthesized code often provided a helpful starting point and saved online searching efforts. However, participants encountered issues with understanding, editing, and debugging code snippets from Copilot, resulting in not necessarily improved task completion times and success rates. These findings align with the results of similar studies [64]. However, a controlled experiment in [65] records a positive effect of code generators when used in introductory programming courses for minors.

The use of generative LLMs and code generators has been scarcely explored in scientific data analysis and not yet for scientific workflows. Liu et al. [23] examine the Codex code generator [56] in the context of data analysis in spreadsheets for non-expert end-user programmers. Moreover, several studies investigate the utilization for data visualization [66, 67]. For example, the study by Maddigan et al. [66] evaluates the efficiency of ChatGPT, Codex, and GPT-3 in producing scripts to create visualizations based on natural language queries. The studies that have the most overlap with our work regarding the intention to support the design of data analysis pipelines are given by Ubani et al. [67] and Zahra et al. [68]. In the case of the former, ChatGPT is used to build a conversational, natural language-based interface between users and the scikit-learn machine learning framework [69] supporting users in several phases of a machine learning project ranging from initial task formulation to comprehensive result interpretation. For the latter, Laminar, a framework for serverless computing, is proposed, which offers possibilities for code searching, summarization, and completion. However, the framework is solely focused on Python implementations.

Methods

This section describes the study methodology. We begin by outlining about the general study approach. Then, we explain the research process for each experiment. Based on related work and the objectives of our research, we state the following research questions:

RQ1: How performant is ChatGPT for comprehending and explaining scientific workflows?
RQ2: How suitable is ChatGPT in suggesting and applying modifications for scientific workflows?
RQ3: How efficient is ChatGPT in extending scientific workflows?

General Study Design

To answer our research questions, we investigate the capabilities of ChatGPT, a widely-used LLM, to comprehend existing workflow descriptions (cf. Study I), to exchange tools used within a workflow (cf. Study II), and to extend a partially given workflow (cf. Study III) using three distinct user studies. We select these use cases as understanding the data flow and the analysis performed is essential for successfully applying scientific workflows. Moreover, exchanging tools and extending a partially given workflow are common use cases in adapting and reusing existing workflows in the work context of domain scientists [6]. For each study, we specially design conversational prompts simulating the interaction between a user working with workflows and ChatGPT. For our studies, we leverage version GPT-3.5 of ChatGPT [70]. We decided to use GPT-3.5 since it is openly available to the public and allows other researchers to reproduce our investigations without additional incurring costs. Moreover, we provide the chat records in the Supplementary Material of this article via the GigaScience database (GigaDB) [71]. Additionally, we develop distinct questionnaires for evaluating the output of ChatGPT by the domain experts for each study. While conducting a study, we present a brief overview of the study's overall goal and the developed questionnaire to the experts. Subsequently, the experts complete the questionnaire independently without the experimenters' support. This procedure is intended as participants were not pressured by a time limit and could freely allocate their time for the study. Furthermore, we intend to avoid a Hawthorne effect, where participants can provide biased responses due to the presence of observers [72].

Participants

Throughout all experiments, we recruited one expert from bioinformatics and three experts working on Earth observation workflows. Using scientific workflows is common in these two areas. In bioinformatics, scientific workflows are an important tool for enabling the automation and documentation of complex data analysis processes, ensuring reproducibility and transparency in research [40]. In Earth observation, scientific workflows streamline the complex process of acquiring, processing, and analyzing vast amounts of satellite and sensor data, enhancing the efficiency and accuracy of environmental studies [79]. Hence, scientific workflows have become commonplace in these two areas. The professions include postdocs and PhD students working at universities. All participants hold a master's degree in their profession and several years of experience in their domain. All experts are between 25 and 40 years old (two female, two male).

Scientific Workflows

In our study, we consider a total of five different workflows. We summarize the used workflows and their details in Table 1. The workflows are taken from the work context of the recruited experts, given their high degree of familiarity and expertise with them. In bioinformatics, we use two workflows that deal with the analysis of genomic data. First, the *crisprseq* workflow, sourced from the *nf-core* repository [27], a hub for best-practice workflows, focuses on analyzing and evaluating gene editing experiments utilizing CRISPR-Cas9 mechanism for genome engineering. Second, the *RS-STAR* workflow, which was implemented by the recruited domain expert and performs differential gene expression analysis using RNA-seq data. The *FORCE2NXF-Rangeland* and *FORCE* are two implementations of an Earth observation workflow that is concerned with analyzing long-term vegetation dynamics in the Mediterranean using the FORCE toolkit [80], which provides processing routines for satellite image archives. The former is implemented using the Nextflow scientific workflow management system [40, 81] and the latter by leveraging Apache Airflow [38, 82]. The third Earth observation workflow called *Grasslands*. builds on previous work [83, 84] aiming at understanding differences in long-term changes in-ground cover fractions specific to European grasslands depending on the definition of endmembers (i.e., unique spectral signatures of a specific material or ground cover) approximating these fractions.

Table 1. Overview of the used workflows. We examine workflows from two scientific domains, i.e., bioinformatics and Earth observation, and two workflow systems (Nextflow and Apache Airflow). For each workflow, we report the number of (high-level) steps and used tools and in which study the workflow is used.

Domain	Workflow	Description	#Steps	#Tools	Study
Bio-informatics	WF1: crisprseq [73] (*Nextflow*)	A bioinformatics data pipeline for analyzing and evaluating gene editing experiments utilizing CRISPR-Cas9 mechanism for genome engineering. Repository: [74] (created 07/2022)	6	9	I
	WF2: RS-Star (*Nextflow*)	The general aim of this workflow is to perform differential gene expression analysis using RNA-seq data. Repository: [75] (created 11/2021)	5	5	I,II,III
Earth Observation	WF3: FORCE2NXF-Rangeland [34] (*Nextflow*)	This workflow analyzes long-term vegetation dynamics in the Mediterranean using the FORCE-toolkit. Repository: [76] (created 11/2020)	9	8	I
	WF4: Grasslands (*Nextflow*)	This workflow aims at understanding differences in long-term changes (1984-2022) in ground cover fractions specific to European grasslands. Repository: [77] (created 08/2023)	6	3	I,III
	WF5: FORCE (*Apache Airflow*)	This workflow focuses on analyzing long-term vegetation dynamics in the Mediterranean using the FORCE-toolkit. Repository: [78] (created 02/2021)	8	8	I

LLM Prompting

The choice and design of prompts entered into a LLM has a decisive influence on the output quality [85] and, in our case, on the suitability of ChatGPT for workflow development and implementation. Our prompts are organized first to provide the context, often including the workflow script, followed by the specific question or instruction under investigation. Suppose the workflow is divided into sub-workflows, possibly distributed over several files. In that case, we first specify the main workflow and then all sub-workflows and task definitions in the order they occur in the main workflow. In our research's initial stages, we experimented with various alternative prompts for each user study, incrementally modifying and enhancing them in response to the outcomes we received. For example, for Study I (i.e., workflow comprehension), we discovered that ChatGPT tends to describe properties of the workflow language or technical aspects instead of workflow characteristics. Such phenomena could be resolved by adding explicit instructions, e.g., "*do not explain nextflow concepts*". We refer to Section Prompt Design Challenges for a detailed discussion of prompt design challenges. We stopped this adjustment process after a few iterations as soon as no more of such artificial artifacts were generated. We have refrained from more extensive prompt engineering since workflow designers are domain experts from diverse fields who cannot be assumed to be specialists in developing and tuning prompts. However, we acknowledge that the choice of wording in our prompts influences the results [85]. We discuss the limitations of our work concerning the chosen study design and prompt strategy selection in more detail in Section Limitations and Future Work.

Study I: Workflow Comprehension

In our first study, we investigate the capabilities of ChatGPT in capturing the actual purpose of a workflow. In other words, we are prompting ChatGPT to explain the purpose of a workflow. In this study, we assess ChatGPT's quality in comprehending and explaining a workflow's purpose in a user study involving workflow experts. Understanding the data flow and the analysis performed constitutes an important aspect of the daily work with scientific workflows. On the one hand, workflows are often precisely adapted to individual research questions, which makes it challenging even for other experts from the same domain to understand them. On the other hand, in many research institutions, an increasing number of legacy workflows whose original authors and contributors are no longer available for maintaining and refining the codes require taking over by new team members. Understanding a workflow is usually a prerequisite for adopting and applying a workflow correctly.

Thus, Study I pursues three goals: how well does ChatGPT perform on (a) identifying the domain and the overall objective of the analysis, (b) reporting the individual computation steps, used tools, their needed input data, produced output data, and (c) explaining research questions for which these analyses are helpful given the workflow description. The first two parts of the study have a reconstructive character, whereas the third is more explorative, requiring ChatGPT to reason beyond the given workflow description. We build a set of five different prompts to evaluate ChatGPT's capabilities concerning the three dimensions. When providing the workflow definition in the prompt, delete all comments within the definition to prevent information leakage. Table 2 depicts the developed prompts. For each workflow, all prompts are executed in one conversation, enabling ChatGPT to use the in- and output of previous prompts as context information. We ask the domain scientists to evaluate answers given by ChatGPT using a feedback questionnaire. The complete questionnaire contains nine items in total and can be found in full-length in the article's Supplementary Material (A). The questionnaire focuses on the correctness of the prompts regarding the aim of the workflow, the explanation, and the forecast of addressed research questions. For four of the nine items, users rate the generated explanations on a 5-point Likert scale (i.e., 1: Strongly Disagree; 5 Strongly Agree.). In addition, three items comprise quantitative evaluations of how many computational steps are correctly detected, how many utilized software tools and programs are accurately identified, and how many valid follow-up research questions. The remaining question items concern the quality of the explanations of the workflow sequence, the description of the tools used, and the results produced. We add a comment field for each item to report issues and errors in the generated explanations if the domain expert does not apply the content.

Results

The results of the expert surveys are presented in the following according to the three subcategories of the questionnaire, i.e., research area and the overall aim of the workflow, explanation of workflow details, and subsequent research questions.

Table 2. Overview of the used prompts to investigate ChatGPT's capabilities in capturing the content of a workflow description (Study I). *[workflow-language]* and *[workflow-text]* represent placeholders for the workflow management system, i.e., Nextflow or Apache Airflow, and the workflow description text. All prompts are executed within one conversation.

ID	Category	Prompt
P1_1	Overall aim	The following text contains a scientific workflow written in *[workflow-language]*: *[workflow-text]* Explain from which research area this workflow originates and describe the general aim of this workflow. Don't explain *[workflow-language]* concepts.
P1_2	Workflow explanation	Explain all individual tasks that are implemented in this workflow. For each task explain which software programs or tools are used in this workflow to perform the task. Don't explain *[workflow-language]* concepts.
P1_3	Workflow explanation	Explain the type of input data and the format of the input data needed for this workflow. Don't explain the workflow itself.
P1_4	Workflow explanation	Explain the overall result of this workflow. For each individual task of the workflow report the type of data that is produced by this task.
P1_5	Research questions	Explain up to 3 research questions for which this workflow is helpful.

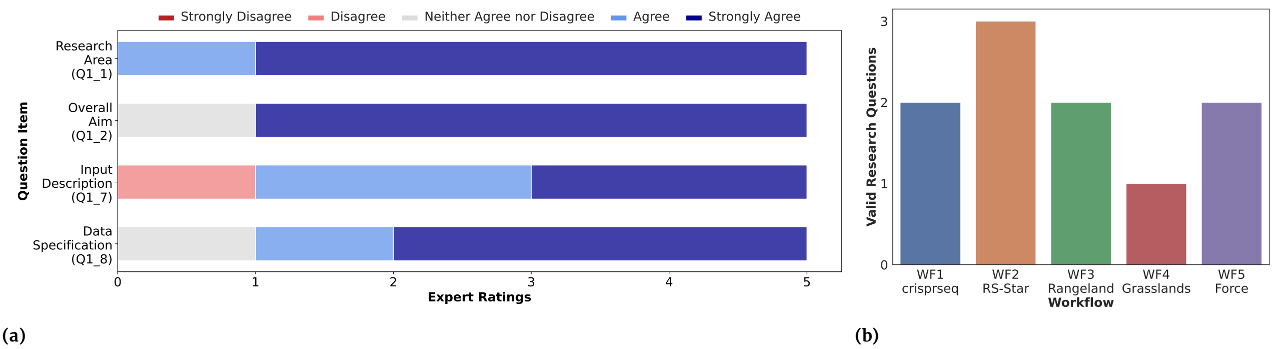

Figure 2. Results from Study I: (a) rating distribution of the domain experts for ChatGPT's capability in identifying the research area and overall aim as well as the input and data description of a workflow. The question item identifier (see Supplementary Material (A)) is given in parenthesis for each row. (b) Number of valid research questions generated by ChatGPT for the different workflows as assessed by the domain experts (question item *Q1_9*). We prompted ChatGPT to output up to three research questions per workflow.

Overall Aim of the Workflow

The first two rows of Figure 2a highlight the rating distribution of the domain experts concerning ChatGPT's explanations for the research area and the overall aim of the workflows. The experts agreed with the explanations generated, indicating a basic understanding of ChatGPT regarding the different workflows (μ = 4.7, σ = 0.68). The lowest agreement, with a score of 4 concerning the research area and 3 for the overall aim, was recorded in the evaluation of the *WF4-Grasslands* workflow. In this case, the expert could not agree with the explanations mainly due to the wrong interpretation of an abbreviation within the workflow description, i.e., *FNF* was misinterpreted as *"fraction of non-forest"*, instead of *fold and fill*. This misunderstanding resulted in the workflow being explained as examining forest regions rather than grasslands.

Workflow Explanation

In general, the participants regard the quality of the explanations given by ChatGPT as high (see Figures 3a and 3b). All computational steps are accurately identified in three of the five workflow descriptions. Moreover, for four of these five workflows, every tool employed is correctly detected, but in *WF5-Force*, two out of eight were missing. The detailed descriptions of the tasks and tools provided by ChatGPT were also judged to be coherent by the experts. Overall, the worst performance is achieved with the output for workflow *WF4-Grasslands* for which only four out of six tasks are correctly extracted and only most of the tools are correctly described. These errors are mainly due to follow-up errors that result from the incorrect recognition of the workflow purpose.

The results of the questions items (see Q1_7 and Q1_8 in Supplementary Material (A)), which assess the produced information about the format and type of input and output data of ChatGPT, can be seen in the two lower rows in Figure 2a. Similar to the previous findings, the information generated for four of the five workflows are evaluated positively (μ = 4.2, σ = 1.0). Again, only the produced information for the workflow *WF4* was assessed as neutral or negative, i.e., input description score of 2 (disagree) and data specification of 3 (neither agree nor disagree).

Research Questions

The last query was concerned with explaining up to three subsequent research questions to a given workflow. Figure 2b shows the result of this question item. ChatGPT achieves only moderate performance, generating just for one workflow, i.e., *WF2-RS-Star*, three valid research questions. In total, out of the 15 generated research questions 10 were correct. These figures suggest that ChatGPT offers only a reduced performance for more explorative tasks.

Study II: Workflow Modification

The second study investigates how much ChatGPT can aid domain experts in modifying and tailoring a workflow in our second study. Researchers usually do not start from scratch when developing workflows but typically adapt or reuse parts of existing workflows from the community [6]. This strategy applies in particular to biomedicine, in which workflows are more widespread and have

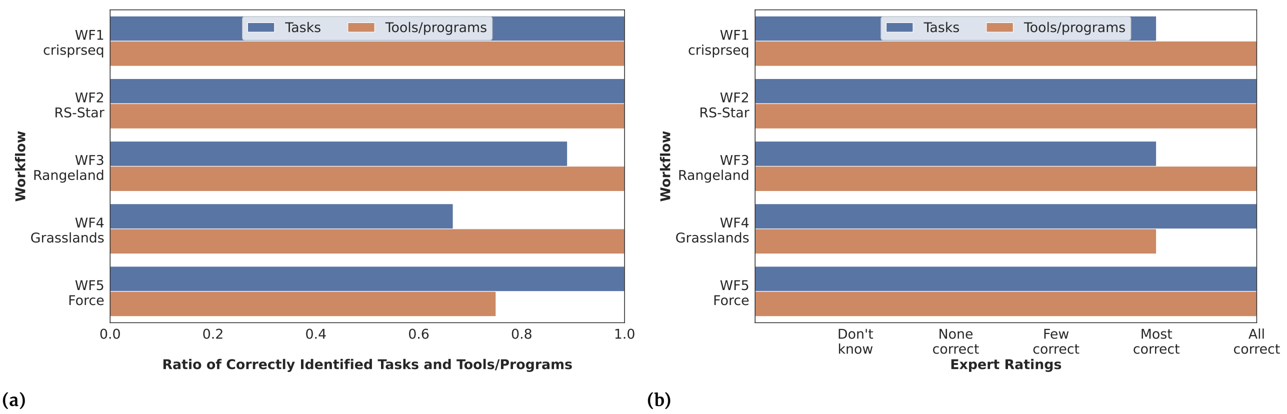

Figure 3. Result statistics highlighting the number of correctly identified tasks and tools of a workflow (a) and their explanation (b). We report separate results per investigated workflow. The results are based on the ratings of the domain experts. Results in (a) correspond to the items *Q1_3* and *Q1_5* and in (b) to *Q1_4* and *Q1_6* of the questionnaire given in the article's Supplementary Material (A).

a longer tradition compared to other domains [86]. For example, genomic workflows will often be applied to a broad spectrum of data originating from different sources, each with its distinctive features and characteristics, making it necessary to adjust the workflow definition for more efficient data processing. Moreover, technological advancements, such as in genome sequencing technology [87], lead to new tools specially developed to leverage the capabilities of the new technologies. The continuous integration of new and alternative scientific tools into existing workflows is essential to conduct state-of-the-art research [88].

In our study, we are particularly investigating the exchange of used tools in the bioinformatics workflow *WF2-RS-Star* whose computational scheme is given in Figure 1a. We select two parts of the workflow to be modified:

i. read trimming and filtering (also called read quality control), originally performed by FASTP [89] and
ii. reference genome indexing and alignment, carried out by STAR [90].

For assessing the workflow modification capabilities of ChatGPT, we build four prompts (see Table 3). The first prompt requests a list of alternative tools for a given workflow step from ChatGPT. The second and third prompts request the recommendation of two alternative tools, including an explanation of the suggestion, a comparison of the selected tools with the tool originally used in the workflow script, and their strengths and weaknesses. With the last prompt, the actual rewriting of the workflow to include the selected tool is requested. We test the inclusion of two alternative tools per computational task, i.e., the prompts *P2_3* and *P2_4* (see Table 3) are carried out once for each tool from the recommendation. Analogous to Study I, we use a questionnaire for evaluating ChatGPT's output by the biomedical domain expert containing 13 items in total. For most items (9 out of 13), the generated texts and explanations concerning methodical differences or pros and cons of the tools should be rated on a 5-point Likert scale. The remaining questions require numerical ratings (2 items), yes/no answers (1), and free text fields (1). Again, we add a comment field for each item to report issues and errors in the generated explanations if the domain expert does not fully apply the content. The complete questionnaire is given in the article's Supplementary Material (B). When conducting the study, we also provide the generated workflow scripts to the domain experts and asked them to execute them on their systems. Furthermore, we request the experts to inspect and correct any non-functional scripts. For the latter, we set a time limit of 20 minutes per tool substitution.

Results

The results are presented in the following according to the two subcategories of the prompts, including the exploration of alternative tools and workflow modification. We summarize the results of the two use cases (i.e., read quality control, reference genome alignment) and each of the two alternative tools when reporting the results.

Tool Exploration

When exploring possible alternative tools, ChatGPT showed a good performance, providing a fully valid list of ten alternative tools using prompt *P2_1* (see Table 3) for both scenarios. The generated output list for both tasks can be seen in Figure 4. However, the domain expert criticized the alternative tools for reference genome alignment specified Bowtie [91] and not its successor Bowtie2 [92]. From these, ChatGPT recommended HISAT2 [93] and Bowtie as alternatives for genome alignment and Trimmomatic [94] and Cutadapt [95] for read quality control. According to the domain expert, all tools are principally valid substitutions, but Bowtie is a rather inappropriate suggestion since it is not specialized for RNAseq data. The results of explaining the tools' methodical differences and strengths and weaknesses can be seen in Figure 5. Essentially, the generated textual explanations were assessed positively, except when describing the methodological differences between the tools. In this case, ChatGPT could offer a convincing explanation in only 50% of the cases. In the other cases, the texts were too general, and differences were not named clearly. In summary, similar to the results from Study I, ChatGPT solves these rather reconstructive tasks well, reaching an average score of 4.1 (min=3, max=5, σ=0.81) when taking the results from items Q2_3 to Q2_7 (see questionnaire in Supplementary Material (B)) into account. These results indicate that ChatGPT could effectively used to explore tools in a given field.

Workflow Modification

We requested ChatGPT to re-write the workflow script for each of the two recommended alternative tools in both use cases, resulting in four modified workflows in total. Table 5 summarizes the results achieved. First of all, none of the generated workflow scripts was entirely correct. Only in one of the four cases, the generated script was (at least) syntactically valid, i.e., if replacing FASTP with Trimmomatic for read quality control, and the domain expert could execute it without further adaptations. However, the script was not a semantically correct modification of the original workflow script since one particular quality control routine (i.e., PolyG trimming) was not reflected in the adopted script. This issue also occurred for Cutadapt, the other alternative tool for quality control. For both

Alternative Tools for Reference Genome Alignment

ChatGPT Output:

Here is a list of 10 alternative tools that can be used for reference genome alignment:

1. ***Bowtie***: A fast and memory-efficient aligner for short DNA sequences.
2. ***BWA*** (Burrows-Wheeler Aligner): A popular aligner for mapping DNA sequences against a large reference genome.
3. ***HISAT2***: A fast and sensitive aligner for spliced alignment of RNA-seq reads.
4. ***TopHat***: A widely used aligner for RNA-seq reads that incorporates splice junctions.
5. ***Subread***: A versatile aligner capable of aligning both DNA and RNA sequences to a reference genome.
6. ***Salmon***: A lightweight aligner specifically designed for accurate quantification of transcript abundance from RNA-seq data.
7. ***STAR*** (Spliced Transcripts Alignment to a Reference): The tool used in the provided workflow, known for its speed and ability to handle splice junctions.
8. ***GSNAP***: A general-purpose aligner that allows for highly sensitive and accurate alignment of short reads against a reference genome.
9. ***Novoalign***: A versatile aligner that supports both DNA and RNA sequence alignment, offering high sensitivity and accuracy.
10. ***Segemehl***: A read mapper optimized for mapping short reads against large genomes with a focus on finding splice junctions accurately.

These are just a few examples of the many alignment tools available in the field of bioinformatics. The choice of tool depends on specific requirements, such as the nature of the data (DNA or RNA), read length, presence of splice junctions, and desired performance characteristics.

Alternative Tools for Read Filtering and Trimming

ChatGPT Output:

Certainly! Here's a list of 10 alternative tools that can be used for read filtering and trimming in biomedical workflows:

1. ***Trimmomatic***: A popular tool for read trimming, adapter removal, and quality filtering.
2. ***Cutadapt***: Used for adapter trimming in high-throughput sequencing data.
3. ***BBDuk***: Part of the BBMap package, it offers read trimming, filtering, and quality control.
4. ***SeqKit***: A fast and lightweight tool for sequence processing, including read trimming.
5. ***Trimgalore***: Specifically designed for trimming and quality filtering of Illumina sequencing data.
6. ***Sickle***: A tool that focuses on quality-based trimming of reads from high-throughput sequencing data.
7. ***AlienTrimmer***: A read trimming tool that uses a statistical approach to identify and remove adapters.
8. ***Prinseq***: Provides various options for read filtering, trimming, and quality control.
9. ***SolexaQA***: Offers quality assessment, read trimming, and error correction for Illumina sequencing data.
10. ***AdapterRemoval***: Specialized for adapter trimming and quality filtering in paired-end sequencing data.

These tools provide different features and algorithms for read filtering and trimming, allowing researchers to choose the one that best suits their specific needs and preferences.

Figure 4. Representation of the output of ChatGPT when requested to provide a list of alternative tools for reference genome indexing and alignment (left) and read quality control (right) using prompt P2_1. All tools are assessed to be valid by the biomedical domain expert.

tools, ChatGPT reported in the explanation text that the tools do not support this feature; however, in reality, they do. This failure could be interpreted as a kind of LLM hallucination [54]. The second task, reference genome indexing and alignment, revealed different issues than the first. Here, the main problem was the correct linking of the two sub-parts of the task, first the index generation and then the computation of the actual alignment. For the former, each tool specifies and uses its distinct data format and defines how to store the index (e.g., saving it in one or multiple files). However, the storing strategy also affects how the output of the indexing task has to be passed on to the input of the alignment computation. In the scripts generated by ChatGPT, the actual step descriptions to invoke indexing and alignment by the tools were (generally) valid. However, the linking of these two needed to be corrected. For example, Bowtie saves its index in multiple files sharing a common file name prefix, which has to be specified as a parameter during alignment. However, in the modified script, the list of all files of a specially created directory was passed to the alignment process. For Bowtie, this problem could be easily fixed by the domain expert, but for HISAT2, it was not that trivial and hence could not be solved in the given time budget of 20 minutes.

To sum up, the study's results indicate that modifying workflow scripts poses considerable challenges for ChatGPT as it requires a detailed understanding of the tool's idiosyncrasy, the exact computations they perform, and the data formats they use.

Study III: Workflow Extension

In the third study, we investigate the capabilities of ChatGPT in extending a scientific workflow given a partial script. As discussed in the motivation for Study II (see Section Study II: Workflow Modification), users often reuse parts of existing workflows from the research community and adapt them to the research question at hand by enhancing the pipeline with additional analyses and computational steps [6]. Moreover, data analysis projects are often exploratory processes, and computation pipelines are incrementally adapted and extended based on executions and findings from previous versions of the workflow, e.g., to include additional data correctness checks, add more differentiated result evaluations and provide advanced result visualizations [96]. In our study, we simulate this incremental exploration process by taking an existing workflow and removing *n* steps at the end of it. We then request ChatGPT to a) enumerate the necessary steps to accomplish the original research goal and b) regenerate the next step using the tool of the original pipeline or by giving a verbal description of the task. For this study, we select one workflow from each research domain for investigation: *WF2-RS-Star* for biomedicine and *WF4-Grasslands* for earth observation. The two workflows were chosen because they offer different implementation characteristics, i.e., *WF2-RS-Star* leverages almost exclusively external tools, whereas *Grasslands* relies more strongly on specially implemented R and Python scripts. Moreover, Study I (see Section Study I: Workflow Comprehension) already showed notable result differences of ChatGPT for both workflows. By choosing these two specific workflows, we aim to encompass a possibly broad spectrum of performance variations. We test ChatGPT's workflow extension capabilities in three scenarios: For WF2-RS-Star, we remove the last step *transcript quantification* as well as the last two steps *transcript quantification* and *format conversion*, forming two extension scenarios. In the case of *WF4-Grasslands*, we remove all steps at the tail of the workflow, including *autoregressive trend analysis* (see schema in Supplementary Material (D)).

Table 4 illustrates the prompts developed for this purpose. This study uses slightly different prompts (see P3_2a and P3_2b) reflecting the different workflow types, i.e., tool- vs. script-based. For the latter, we include additional instructions to a) specify the programming language of the script and b) ask the domain expert for a verbal description of the computational steps to be implemented. See Supplementary Material (E) for the verbal description provided by the earth observation expert. The questionnaire for evaluating the generated outputs consisting of seven items can be found in the article's Supplementary Material (C).

Results

The results are presented in the following according to the two subcategories of the prompts, i.e., workflow exploration and extension.

Table 3. Overview of the used prompts to investigate ChatGPT's capabilities in swapping used tools in bioinformatic workflows (Study II). Information in square brackets specifies placeholders for concrete information regarding the workflow or the tool to be replaced.

ID	Category	Prompt
P2_1	Tool exploration	The following text contains a *[domain]* workflow written in *[workflow-language]*: *[main-workflow]* The following snippets contain the source code for the step of the workflow which uses *[tool]* to perform *[step]*: *[step-source-code]*. Please provide a list of 10 alternative tools to perform *[tool]*.
P2_2	Tool exploration	The following text contains a *[domain]* workflow written in *[workflow-language]*: *[main-workflow]* The following snippets contain the source code for the step of the workflow which uses *[tool]* to perform *[step]*: *[step-source-code]*. Alternative tools for *[step]* are: *[list-of-tools]* Which of the tools would you recommend as most suitable alternative for *[step]* in the given workflow. Please name the two alternatives and give an explanation why these tools are especially advisable for the given workflow.
P2_3	Tool exploration	*[original-tool]* and *[alternative-tool]* are two tools for *[step]* in *[domain]* workflows. First, explain the differences between the tools and the underlying approaches. Second, name strenghts and weaknesses of both tools.
P2_4	Workflow modification	The following text contains a *[domain]* workflow written in *[workflow-language]*: *[main-workflow]* The following snippets contain the source code for the step of the workflow which uses the *[tool]* to perform *[step]*: *[step-source-code]* Please re-write the code of the workflow and the proccess to use *[alternative-tool]* instead of *[original-tool]*. The number of parameters of the individual process descriptions may have to be adjusted. Please explain features / options of *[original-tool]* which are not supported in *[new-tool]*.

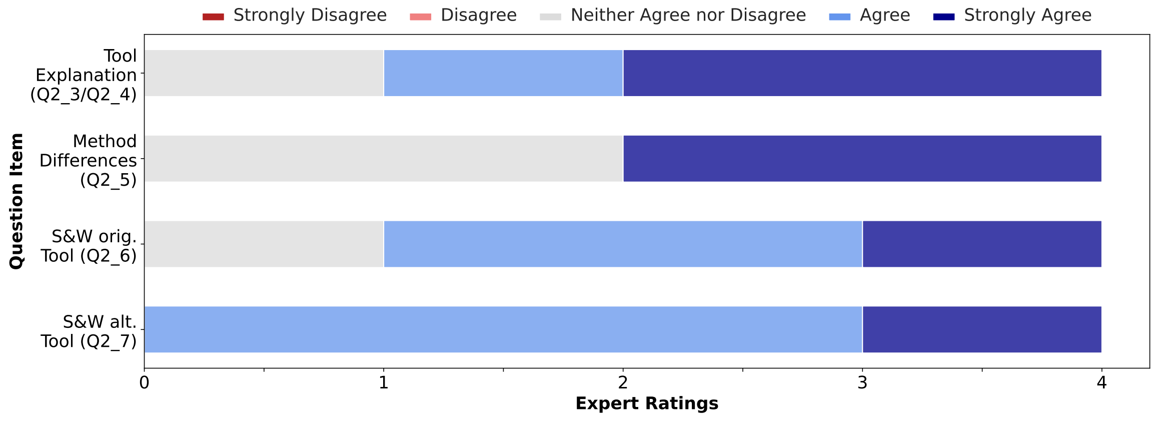

Figure 5. Overview of the rating distribution of the biomedical domain expert for ChatGPT's capability for explaining alternative tools, methodical differences, and strengths and weaknesses (S&W) of the tools. The question item identifier (see Supplementary Material (B)) is given in parenthesis for each row.

Workflow exploration

For describing further computational steps necessary to accomplish a specific research goal given a partial workflow, ChatGPT showed mixed results. The LLM provides a correct list of suitable steps in two of the three scenarios. Also, the tools and methods for implementing the steps suggested by ChatGPT were valid. However, both domain experts criticize that the specifications for the necessary steps and the proposed tools tend to be rather generic and generalized. For instance, for extending the *WF4-Grasslands* workflow the earth observation expert commented:

> *Overall, the proposed workflow is very generic and does not provide a clear roadmap for the analyses. It also proposes to use very simplistic and often imperfect approaches.*

Overall, the results confirm the findings from the two previous studies that ChatGPT shows weaknesses in more exploratory tasks.

Workflow extension

Using the prompts P3_2a and P3_2b (see Table 4), we request ChatGPT to re-construct the last removed computational step in each extension scenario. Table 6 summarizes the results achieved. Like the results from Study II, ChatGPT shows considerable weaknesses in the automatic extension of workflows. None of the generated workflow scripts was executable without the intervention of the domain expert. A clear difference is revealed when comparing the two domains, biomedicine and earth observation. In the former case, the generated workflow scripts are (at least) of such a quality that the domain expert could successfully correct them within 20 minutes. In the generated scripts, mainly syntactical errors occurred (e.g., incorrect usage of variable identifiers, incomplete input definitions, or missing specification of parameters), which could be easily corrected. However, the calls to the respective programs to perform the two tasks were correct.

In contrast, the generated extension for *WF4-Grasslands* was of considerably lower quality. In this case, several syntactic and semantic errors occurred, e.g., the script uses a non-existing library function, no parallelization code is included, and not all requested computations are performed. In this state, the domain expert could not resolve the large number of problems within 20 minutes. However, when interpreting these results, one must remember that the task in this scenario is also significantly more difficult. Instead of a short task description and specification of a tool to be used, ChatGPT has to design and generate the source code for a complex

data analysis procedure containing multiple sub-steps.

Discussion

We conducted three studies to investigate the capabilities of using ChatGPT for comprehending, modifying, and extending scientific workflows. We discuss our methodology and the results in the following.

Comprehending Scientific Workflows

Study I was designed to answer **RQ1** by evaluating ChatGPT's performance in comprehending existing workflows. The domain experts assessed that ChatGPT is good at this task while showing slight differences between the investigated research domains. In particular, the explanations for workflow *WF4-Grasslands* revealed considerable performance drops. Unlike the other workflows investigated, this one uses multiple proprietary R and Python scripts instead of leveraging external tools for assembling data processing pipelines. The lack of standardized tools makes workflow comprehension more challenging since ChatGPT has to interpret complex processing logic and has fewer possibilities to leverage static information, like the description of the general purpose of an established bioinformatics tool, seen through its training while generating the response. In addition, code quality and its readability may strongly influence the results for workflows containing proprietary scripts. For instance, one major problem while explaining *WF4-Grass-lands* in Study I was the misinterpretation of the abbreviation *"fnf"* as *"fraction of non-forest"*, instead of *"fold and fill"*. Such customized and ambiguous terms challenge LLMs and reduce their applicability.

In an ablation experiment, we replaced all abbreviations in WF4 with their complete form and re-generated the explanations of prompt P1_1, requesting the overall purpose of the workflow. Having access to the full forms in the script, ChatGPT's output was much better, even if the exact goal of the workflow was still only met on a rather abstract level:

> *[...] aimed at extracting detailed information about land cover dynamics, vegetation phenology, and environmental changes in a specific region using satellite data.*

We also did a reverse experiment using *WF2-RS-Star* by replacing all full-form task names with abbreviations in the workflow script (but keeping the name of the tools called fixed). In this case, the results did not change considerably, highlighting the stronger robustness of ChatGPT against such changes.

Modifying Scientific Workflows

We answer **RQ2** in Study II by evaluating the modification performance of ChatGPT. To this end, we requested the LLM to substitute the leveraged tools for two computational tasks, read quality control and reference genome alignment, in the biomedical workflow *WF2-RS-Star*. The study results suggest that ChatGPT can effectively explore and explain alternative tools in the field, possibly shortening the time the experts spend searching for suitable replacements on the web. In contrast, the results also indicate that ChatGPT rather poorly supports the generation of workflow scripts for using these alternative tools. In only one scenario, i.e., substituting *FASTP* with *Trimmomatic*, the produced script could be run without syntactical errors, and in one other scenario, i.e., replacing STAR with Bowtie, the script could be fixed within 20 minutes to be syntactically and semantically valid. In the used version of ChatGPT and the selected setup, an increase in efficiency cannot be recorded or anticipated, highlighting the need for further research efforts. However, when interpreting the results, it is essential to remember that ChatGPT is a general-purpose LLM rather focusing on human language. A potential option for improvement could be testing generative models more strongly adapted to programming code, such as GitHub Copilot or Code Llama [19]. Moreover, alternative prompting strategies, which adapt the workflow iteratively, could help avoid errors, e.g., when linking the source code of existing tasks with the new task descriptions. We refer to the discussion of other prompting solutions in Section Limitations and Future Work. We emphasize again that workflows and the development of tools in bioinformatics, particularly in the field of genomic analyses, are based on a more extended history and have established more robust and more widely-used software than other scientific fields. Accordingly, it is reasonable to infer that the outcomes, such as for suggesting alternative tools, reflect an upper boundary of quality, suggesting that encountering difficulties is more likely in less explored application areas due to insufficient data availability.

Extending Scientific Workflows

Finally, we investigate **RQ3** by conducting Study III. To this end, we requested ChatGPT to extend an existing (partially given) workflow to achieve specific goals. The study results confirm the findings from the two previous studies and emphasize ChatGPT's difficulties in solving more complex and exploratory problems. In this case, explaining the necessary steps to answer the given research questions and the generation of the workflow script for the next step offered (partly) severe issues. Similar to the results of Study I, the picture is mixed regarding the different research domains, earth observation and bioinformatics. For the latter, the generated scripts form a relatively good basis for the implementation, having only (minor) syntactical issues that the expert could quickly fix. In contrast, in the case of earth observation, the script quality was considerably worse, hindering a fast correction by the expert. These results imply that efficient user support is possible for pipelines mainly leveraging external tools. However, further research is necessary to investigate user-support strategies for workflows applying specially implemented analysis scripts. Specifically, human-in-the-loop approaches that involve human experts more closely in the code generation process could also be helpful here (see Section Limitations and Future Work).

Scientific Workflows in LLM Training Data

LLMs are trained on large amounts of textual data from the web, including programming code and workflow scripts [43]. Therefore, it is crucial to consider whether and to what extent an LLM was already able to access the workflow scripts of our study during its pretraining. According to public information [97], ChatGPT was trained on data gathered until September 2021, meaning that initial versions of two of the five tested workflows (i.e., *WF3-FORCE2NXF-Rangeland* and *WF5-Force*) could have been part of the LLM's training routine. However, the specific training dataset used for ChatGPT is not accessible to the public, preventing a conclusive assessment. To attain a more precise estimate of the potential number of workflow scripts within the training data in general, we initiated searches for scientific workflow repositories on GitHub. We leverage the repository search engine of the website [98] and use the names of four widely-used workflow management systems, i.e., Apache Airflow, Nextflow, Snakemake, and Taverna as a query term. We filter all repositories with creation data less than 2021-09-01 from the query results. Of course, the results must be interpreted carefully since not every repository containing the name has to deal with scientific workflows, even if the names are very peculiar. Detailed statistics from our search results can be found in this article's Supplementary Material (F). As of 09/2021, there were between 352 and 1,900 repositories containing one of the workflow system names in their description. Moreover, the results highlight the in-

Table 4. Overview of the used prompts to investigate ChatGPT's capabilities in extending a given partial workflow (Study III). We distinguish two types of prompts: workflow exploration and workflow extension. For the latter, we developed two variants specially designed for tool- (P3_2a) and script-based workflows (P3_2b).

ID	Category	Prompt
P3_1	Workflow Exploration	The following text contains a *[domain]* workflow written in Nextflow:<br>*[workflow-description]*<br>The workflow should be used to *[overall-goal]*. Which steps are missing in order to perform *[overall-goal]*? Please specify only the absolutely necessary steps. For each step name up to three *[domain]* tools that can be used to perform the task.
P3_2a	Workflow Extension	The following text contains a *[domain]* workflow written in Nextflow:<br>*[workflow-description]*<br>Please extend to the given workflow to include one further step which *[step-description]* using *[tool]*. Please specify the new process description in a file at *[file-name]*. Please use version 2 of the Nextflow workflow language. The new process should take the output of *[predecessor-step]* as input.
P3_2b	Workflow Extension	The following text contains a *[domain]* workflow written in Nextflow:<br>*[workflow-description]*<br>Please extend to the given workflow to include one further task which performs *[step]* using an *[programming-language]* script. For this, please generate an *[programming-language]* script, stored in *[script-file-name]*, which performs the following computations:<br>*[verbal-task-description]*<br>Next to the *[programming-language]* script generate the Nextflow process description in a file named *[process-file-name]* and the updated workflow. Please use version 2 of the Nextflow workflow language. The new process should take the output of *[predecessor-step]* as input.

creasing popularity of workflows since, for all systems except for Taverna, the number of repositories has almost doubled over the last two years. We also checked the number of Nextflow pipelines available in nf-core. As of September 2021, 35 pipe-lines were published, and 19 were under development [27]. Today, nf-core hosts 55 published pipelines and 33 in development. In summary, we can hypothesize from these results that ChatGPT can likely rely only on a relatively small base of workflow scripts during its training compared to classical programming code (e.g., GitHub currently hosts over 3.9 million Java and over 2.2 million Python repositories, which we determined by using GitHub repository search and the search queries "*language:Java*" and "*language:Python*", making user support for workflow design and implementation particularly challenging.

Prompt Design Challenges

While creating prompts for the studies, we identified several challenges and issues that arose while interacting with ChatGPT.

Representation of Workflows

For the representation of the workflow scripts, there is no straightforward option on how to include them in a prompt. The workflow descriptions are often spread over several files containing sub-workflows and task descriptions. In our approach, we first specify the main workflow and then all sub-workflows and task descriptions in order of occurrence. However, there might be other, more efficient prompt solutions (with respect to the generative language model). Furthermore, the workflow scripts might exceed the maximum allowed input length of the language model, e.g., ChatGPT variants allow only for 4K to 16K words / tokens [97] in the input sequence. In particular, workflows heavily relying on specially implemented scripts having hundreds of code lines will face this issue.

Loss of Focus

Some of the prompts are very long due to the specification of the entire workflow script, which challenges ChatGPT to maintain focus. Adding additional instructions to the prompt helped to avoid or reduce this phenomenon, e.g. for the explanation use case (Study I) we added to the prompt *"Don't explain nextflow concepts"* (see P1_1 and P1_2 in Table 2) and *"Don't explain the workflow itself"* (P1_3) to prevent ChatGPT to generate outputs describing features of the workflow management system or the complete workflow when requesting input data specification.

Technological Details

In some cases, adaptation to technological details of the specified workflows were necessary. For example, the Nextflow system offers two language versions for describing processing pipelines. The Nextflow workflows in our study all used the new version of the language. However, when extending workflows in Study III, we had to specify the desired version (see P3_2 in Table 4) to get the correct output. This observation is surprising since the partially given workflow is already in the respective version. Interestingly, this was only necessary for the workflow extension but not for their modification (P2_4 in Table 3) in which the phenomena did not occur.

In summary, the efficient and effective formation of prompts offers a wide range of possible solutions. In our study, we identified initial clues and difficulties, but further research is needed to detect further potential for improving the interaction between domain experts and ChatGPT and generative LLMs in general.

Limitations and Future Work

In the following, we highlight the limitations of this work that merit further research.

Study Design

In each of our three studies, we created and provided the prompts for testing ChatGPT's capabilities concerning the different use cases and the domain experts only evaluated the outputs of ChatGPT, leading to a rather indirect interaction between the domain scientist and the LLM. An alternative design for the study would be to have the experts interact directly with ChatGPT by developing and refining the prompts independently. In addition to assessing the capabilities of ChatGPT, this would have the advantage of gaining initial insights into interaction forms and patterns of the different experts with ChatGPT. Moreover, this would allow for improved customization of the prompts to the particular research domain and

Table 5. Overview of the results of the workflow modification use case in which the tools performing a specific task are replaced by alternative ones. For each task, we provide the original tool (in parenthesis) and the investigated alternative ones suggested by ChatGPT. For each combination, we highlight (✓=yes, ×=no) whether the generated workflow script could be executed (Exec.), whether it is semantically valid (Val.), and whether it could be fixed within 20 minutes (Fix). For the latter, (✓) indicates cases where the script could be fixed to be executable but not entirely semantically correct. Moreover, we provide excerpts from the domain expert's comments.

Task	Alt. Tool	Exec.	Val.	Fix	Problems / Comments
Read quality control (*FASTP [89]*)	Trimmomatic [94]	✓	×	(✓)	• Missing implementation to perform special quality control feature (i.e. PolyG trimming)<br>• Invalid description of workflow differences:<br>- ChatGPT stated that polyG trimming isn't available in Trimmomatic, but it actually is, however extra implementation for data adaptation is needed<br>- Differences of the generated output files not described accurately
	Cutadapt [95]	×	×	(✓)	• Wrong program call: syntactically incorrect specification of two parameters<br>• Missing provision of valid adapter sequences<br>• Invalid description of differences of the workflow script:<br>- ChatGPT stated that polyG trimming isn't available in Cutadapt, but it actually is<br>- Differences of the generated output files not described accurately
Genome indexing & alignment (*STAR [90]*)	HISAT2 [93]	×	×	×	• Invalid definition and linkage of in- and output between genome index generation and alignment<br>• Syntactically incorrect call of the alignment process<br>• Does not take parameter *strandedness* of the input data into account<br>• Output files aren't generated correctly
	Bowtie [91]	×	×	✓	• Invalid definition and linkage of in- and output between genome index generation and alignment<br>• Wrong output definition of genome indexing task

the idiosyncratic properties and characteristics of each workflow. Extended optimization of the prompting strategy by the domain scientist could lead to better results but reduce potential time savings in solving the actual task. Our study design was motivated by the fact that the experts had strongly limited time budgets for the study. For example, even for evaluating ChatGPT's outputs in Study I, the experts already needed up to three hours to accurately check the generated explanations. A study design that envisages direct interaction involves high efforts in terms of introduction and explanation to ChatGPT and prompting strategies for the domain scientists, thus limiting the scope of research questions that can be investigated. In addition, the selected study design has the advantage of using the same prompts for the different domains, which contributes to better comparability of the results and eliminates the influence of differences for individual prompt differences.

In our study, we focused solely on ChatGPT as generative language model. However, there are many other general-purpose models available (e.g., PaLM-2 [45], Gemini [101] or Llama-2 [19]) as well as models more specially designed for programming tasks (e.g., GitHub Copilot, Code Llama [102] or OpenAI Codex [103] publicly available and worth investigating. Furthermore, recent research showed that placebo effects can undermine the validity of study results when user expectations are altered through the presence of an AI [104, 105, 106] (i.e., LLM) or a novel superior technology [107, 108] that improves user capabilities. Our studies only highlight the results of ChatGPT in the version used (GPT-3.5) but do not claim generalizability for other LLMs. In future work and in the case of using LLMs, placebo conditions must be included to avoid findings that are not a result of increased user expectations towards the capabilities of ChatGPT.

Proprietary and Closed-Source LLMs

While ChatGPT and other proprietary LLMs offer remarkable natural language understanding and generation capabilities, they have inherent limitations that can hinder their utility in scientific workflow development. One significant limitation is their lack of transparency in their underlying algorithms and training data, restricting results' direct reproducibility and interpretability. Users typically interact with these models through an application programming interface (API) or web application provided by the company or organization that developed it rather than having direct access to the underlying code or data, which often makes it impossible to trace any changes to the model and, thus, to the results achieved. Moreover, the closed nature of these models restricts researchers' ability to customize or fine-tune them for specific tasks, limiting their adaptability to diverse research domains. An alternative is using freely available, open-source LLMs, like BLOOM [20] or LLaMA [19], where users have more control over model changes. Nonetheless, the data basis and training procedure are often not fully transparent, even with these models. Moreover, it is essential to note that running such models demands substantial computational resources and the corresponding technical expertise.

Prompting Strategy

Next to other models, the prompts used in our studies also constitute a limiting factor. We cannot exclude the possibility that other prompts, using a different structure or wordings, may achieve better results for the investigated use cases. Moreover, in our approach, we used only the workflow description's source code, without comments, to form the prompt. There are several strategies for enriching the prompt with additional context information, which can lead to improved results. For instance, including available documentation artifacts of the workflow, e.g., descriptive texts in the source code repository or the publication introducing (parts of) the workflow, may ease the processing of the text. Similarly, utilizing descriptions or manuals for the tools employed could also contribute to better results.

In addition, the prompts for individual aspects of the workflows could be revised to obtain more differentiated results. For instance, the prompts for exploring alternative tools (see P2_1 and P2_2 in Table 3) could be extended to request further semi-structured information, such as the programming language used, software license, release date, and development history, guiding the tool selection process. Information on the development history, for example, could help domain experts differentiate between well-established, trusted tools and new developments. This would additionally enhance result interpretation since there might be limited information available for recent advancements, potentially leading to their (yet) insufficient representation in the LLM.

Furthermore, our approach is based on a linear prompt ex-

Table 6. Overview of the results of the workflow extension use case in which we provide ChatGPT a partial workflow and request the LLM to extend it by one further computational step. For each investigated use case, we highlight (✓=yes, ×=no) whether the generated workflow script could be executed (Exec.), whether it is semantically valid (Val.), and whether it could be fixed within 20 minutes (Fix). For the latter, (✓) indicates cases where the script could be fixed to be executable but not entirely semantically correct. Moreover, we provide excerpts from the domain expert's comments.

Workflow	Task/Tool	Exec.	Val.	Fix	Problems / Comments
WF2 RS-Star	Transcript quantification/ *Cufflinks* [99]	×	×	✓	• Syntax errors: process definition for CUFFLINKS declares one input channel but two were specified<br>• Input tuple does not match input set cardinality declared by process definition<br>• Wrong variable name: sorted_bam (wrong) instead of sample_bam
	Format conversion/ *SAMtools* [100]	×	×	✓	• Syntactical errors: Usage of wrong variable name (sample_sam)<br>• Incorrect syntax for connecting the new task to the previous one SAMTOOLS(STAR_ALIGN.sample_sam) (wrong) vs. SAMTOOLS(STAR_ALIGN.out.sample_sam) (correct)
WF4 Grasslands	AR analysis/ *R script*	×	×	×	• Input to the R script is a path to a directory, not a TIFF file<br>• Incorrect use of remotePARTS library: There is no function called *autoTrend* in this package<br>• Calculation needs to be parallelized (as specified in the request)<br>• Computation should be implemented for four types of inputs, namely: GV, NPV, SOIL, and SHADE.<br>• Desired outputs from the AR model needs to be retrieved and written out (missing)<br>• Script declares a Conda environment (Python), not R environment.

ecution without feedback or revision opportunities. Iterative prompting strategies, such as chain-of-thought [109] or graph-of-thoughts prompting [110], self-debugging [111] or self-adapting approaches [112], can potentially enhance results. These strategies offer dynamic interaction approaches with LLMs, enabling continuous improvement and adaptation of responses through successive refinements. This facilitates, for example, the correction of errors encountered during workflow modification and extension, such as syntax errors in tool invocation, data format mismatches or invalid task linking, by utilizing the LLM output as input for the subsequent iteration. In addition to other technical possibilities of the LLM conversation, prompt customization and adaptation by human experts offer strong potential for improvement [113, 114]. Using such human-in-the-loop approaches allows for explicitly addressing the observed problems, especially in workflow modification and expansion, in a semi-automatic process.

Stochastic Generation

In addition to alternative prompting strategies, it should be emphasized that the generated texts are subject to stochastic sampling processes, which can lead to deviations even when reusing the exact same prompts multiple times. In most LLMs, the influence of this phenomenon can be managed using the *temperature* hyperparameter, which regulates how the model samples the next word by adjusting the probability distribution. Higher temperature values soften the distribution, leading to more diverse and creative outputs, allowing the model to explore a broader range of possibilities. On the other hand, a lower temperature tends to produce more conservative and predictable outputs, as the model is more likely to choose tokens with higher probabilities. Thus the parameter serves as a knob to adjust the balance between exploration (generating diverse outputs) and exploitation (producing more likely outputs). An extensive investigation of the parameter's influence represents an interesting future research question.

Limited Number of Domain Experts

In the context of our studies, only four domain experts evaluated the outputs of ChatGPT. In some cases, generated explanations were assessed by one person only (e.g., Study II). This low number of experts limits the validity and generalizability of the results and offers the risk of subjective bias. However, recruitment for such studies is difficult because the number of potential participants is small and they often have strongly limited time budgets, making study design challenging. Please note that for experts in the field, even "just" familiarizing themselves with an unfamiliar workflow is a challenging and time-consuming endeavor.

Investigated domains and selected workflows

Our study explores real-world workflows from the two domains, bioinformatics and earth observation. Of course, these only represent part of the full range of workflows in the natural sciences. It constitutes an exciting follow-up research question: how suitable ChatGPT and other generative LLMs are in other research contexts, such as climate research [33] and astronomy [32], and whether it is possible to identify categories or groups of domains which are particularly well (or poorly) supported. Furthermore, we only examined two workflow systems, Nextflow and Apache Airflow, leaving other alternatives, such as Snakemake, Taverna, and Pegasus, for future work. Investigating other workflow systems is especially interesting because they specify different languages with different complexity levels and supported features for designing analysis pipelines. For example, the language of Apache Airflow, which prioritizes flexibility and extensibility through its integration with the general-purpose programming language Python, expands the potential output scope compared to more deterministic languages such as the Common Workflow Language (CWL)[115, 116]. Consequently, this increased variability likely poses considerable challenges for LLMs. For this, a thorough analysis of how the idiosyncrasy of the leveraged workflow language influences the outcomes produced by LLMs would be beneficial in offering guidance to practitioners.

Explored Use Cases

This work focused on comprehending, modifying, and extending workflows with ChatGPT. These use cases represent only a partial scope of user support opportunities and are worth considering and evaluating other use cases. For instance, migrating workflows implemented in legacy workflow management systems to more recent ones, e.g., transforming Taverna [86] scripts to Snakemake or Nextflow, or adapting them to different infrastructure stacks poses an interesting research question. Moreover, user support in workflow debugging, error identification, or optimization, as done in classical programming [62], would be a valuable contribution to research scientists.

Conclusion

The significance of large-scale data analysis workflows in advancing research in the natural sciences is growing steadily. Developers of such workflows, primarily researchers from diverse scientific fields, are challenged with the increasing complexity and scale of their analyses, which involve (next to their domain knowledge) working with different frameworks, tools, programming languages, and infrastructure stacks. Although a few tools for creating and maintaining workflows are available, improving user efficiency remains an open research area. In this work, we contribute to this situation by evaluating the suitability of ChatGPT for comprehending, modifying, and extending scientific workflows. In three user studies with four researchers from different scientific domains, we evaluated the correctness of ChatGPT regarding explainability, exchange of software components, and extension when providing real-world scientific workflow descriptions. Our results show a high accuracy for comprehending and explaining scientific workflows while achieving a reduced performance for modifying and extending workflow descriptions. These findings clearly illustrate the need for further research in this area.

Additional Files

Supplementary Material: Complete Questionnaires for user studies I-III, schema of workflow *WF4-Grasslands*, verbal task description for user study III and statistics of the GitHub Search results.

Data Availability

The source code of all investigated workflows are available via [74, 75, 76, 77, 78]. We provide the complete LLM chat outputs as supplementary material to this manuscript via the GigaScience database (GigaDB) [71].

Declarations

List of Abbreviations

LLM: Large Language Model; EUP: End-User Programming

Competing Interests

The authors declare that they have no known competing financial interests or personal relationships that could have appeared to influence the work reported in this paper.

Funding

This work is supported by *German Research Foundation (DFG)*, CRC 1404: "FONDA: Foundations of Workflows for Large-Scale Scientific Data Analysis" (Project-ID 414984028).

Author's Contributions

Mario Sänger: Conceptualization, Methodology, Investigation, Visualization, Writing - Original Draft **Ninon De Mecquenem**: Resources, Data Curation, Writing - Review & Editing **Katarzyna Ewa Lewińska**: Resources, Data Curation, Writing - Review & Editing **Vasilis Bountris**: Resources, Data Curation, Writing - Review & Editing **Fabian Lehmann**: Resources, Data Curation, Writing - Review & Editing **Ulf Leser**: Conceptualization, Writing - Review & Editing, Project administration, Funding acquisition **Thomas Kosch**: Conceptualization, Methodology, Writing - Original Draft , Writing - Review & Editing, Validation, Project administration

References

1. Davidson SB, Freire J. Provenance and Scientific Workflows: Challenges and Opportunities. In: Proceedings of the 2008 ACM SIGMOD International Conference on Management of Data SIGMOD '08, New York, NY, USA: Association for Computing Machinery; 2008. p. 1345–1350.
2. Cohen-Boulakia S, Belhajjame K, Collin O, Chopard J, Froidevaux C, Gaignard A, et al. Scientific workflows for computational reproducibility in the life sciences: Status, challenges and opportunities. Future Generation Computer Systems 2017;75:284–298.
3. Wratten L, Wilm A, Göke J. Reproducible, scalable, and shareable analysis pipelines with bioinformatics workflow managers. Nature methods 2021;18(10).
4. Ison J, Ienasescu H, Chmura P, Rydza E, Ménager H, Kalaš M, et al. The bio.tools registry of software tools and data resources for the life sciences. Genome Biology (Online Edition) 2019;20(1).
5. Ferreira da Silva R, Casanova H, Chard K, Altintas I, Badia RM, Balis B, et al. A Community Roadmap for Scientific Workflows Research and Development. In: Workshop on Workflows in Support of Large-Scale Science (WORKS); 2021. .
6. Cohen-Boulakia S, Leser U. Search, adapt, and reuse: the future of scientific workflows. ACM SIGMOD Record 2011;40(2):6–16.
7. Deelman E, Peterka T, Altintas I, Carothers CD, van Dam KK, Moreland K, et al. The future of scientific workflows. The International Journal of High Performance Computing Applications 2018;32(1):159–175.
8. Myers BA, Rosson MB. Survey on user interface programming. In: Proceedings of the SIGCHI conference on Human factors in computing systems; 1992. p. 195–202.
9. Barricelli BR, Cassano F, Fogli D, Piccinno A. End-user development, end-user programming and end-user software engineering: A systematic mapping study. Journal of Systems and Software 2019;149:101–137.
10. Lau S, Srinivasa Ragavan SS, Milne K, Barik T, Sarkar A. Tweakit: Supporting end-user programmers who transmogrify code. In: Proceedings of the 2021 CHI Conference on Human Factors in Computing Systems; 2021. p. 1–12.
11. Peitek N, Bergum A, Rekrut M, Mucke J, Nadig M, Parnin C, et al. Correlates of Programmer Efficacy and Their Link to Experience: A Combined EEG and Eye-Tracking Study. In: Proceedings of the 30th ACM Joint European Software Engineering Conference and Symposium on the Foundations of Software Engineering ESEC/FSE 2022, New York, NY, USA: Association for Computing Machinery; 2022. p. 120–131.
12. Kosch T, Karolus J, Zagermann J, Reiterer H, Schmidt A, Woźniak PW. A Survey on Measuring Cognitive Workload in Human-Computer Interaction. ACM Computing Surveys 2023 jan;.
13. Li TJJ, Azaria A, Myers BA. SUGILITE: creating multimodal smartphone automation by demonstration. In: Proceedings of the 2017 CHI conference on human factors in computing systems; 2017. p. 6038–6049.
14. Sereshkeh AR, Leung G, Perumal K, Phillips C, Zhang M, Fazly A, et al. VASTA: a vision and language-assisted smartphone task automation system. In: Proceedings of the 25th international conference on intelligent user interfaces; 2020. p. 22–32.
15. Tamilselvam SG, Panwar N, Khare S, Aralikatte R, Sankaran A, Mani S. A visual programming paradigm for abstract deep learning model development. In: Proceedings of the 10th Indian Conference on Human-Computer Interaction; 2019. p.

1–11.
16. Coronado E, Deuff D, Carreno-Medrano P, Tian L, Kulić D, Sumartojo S, et al. Towards a modular and distributed end-user development framework for human-robot interaction. IEEE Access 2021;9:12675–12692.
17. Li TJJ. End user programing of intelligent agents using demonstrations and natural language instructions. In: Proceedings of the 24th International Conference on Intelligent User Interfaces: Companion; 2019. p. 143–144.
18. Ouyang L, Wu J, Jiang X, Almeida D, Wainwright C, Mishkin P, et al. Training language models to follow instructions with human feedback. Advances in Neural Information Processing Systems 2022;35:27730–27744.
19. Touvron H, Lavril T, Izacard G, Martinet X, Lachaux MA, Lacroix T, et al. Llama: Open and efficient foundation language models. arXiv preprint arXiv:230213971 2023;.
20. Scao TL, Fan A, Akiki C, Pavlick E, Ilić S, Hesslow D, et al. Bloom: A 176b-parameter open-access multilingual language model. arXiv preprint arXiv:221105100 2022;.
21. Bimbatti G, Fogli D, Gargioni L. Can ChatGPT Support End-User Development of Robot Programs? Proceedings of the Workshops, Work in Progress Demos and Doctoral Consortium at the IS-EUD 2023 2023;.
22. Sobania D, Briesch M, Hanna C, Petke J. An analysis of the automatic bug fixing performance of chatgpt. arXiv preprint arXiv:230108653 2023;.
23. Liu MX, Sarkar A, Negreanu C, Zorn B, Williams J, Toronto N, et al. "What It Wants Me To Say": Bridging the Abstraction Gap Between End-User Programmers and Code-Generating Large Language Models. In: Proceedings of the 2023 CHI Conference on Human Factors in Computing Systems; 2023. p. 1–31.
24. White J, Hays S, Fu Q, Spencer-Smith J, Schmidt DC. Chatgpt prompt patterns for improving code quality, refactoring, requirements elicitation, and software design. arXiv preprint arXiv:230307839 2023;.
25. Surameery NMS, Shakor MY. Use chat gpt to solve programming bugs. International Journal of Information Technology & Computer Engineering (IJITC) ISSN: 2455-5290 2023;3(01):17–22.
26. Ewels P, Peltzer A, Fillinger S, Patel H, Alneberg J, Wilm A, et al. The nf-core framework for community-curated bioinformatics pipelines. Nature biotechnology 2020;38(3).
27. nf-core; 2024. [Online; accessed April 8th 2024]. https://nf-co.re.
28. Almarie B, Teixeira PE, Pacheco-Barrios K, Rossetti C, Fregni F. The Use of Large Language Models in Science: Opportunities and Challenges. Principles and Practice of Clinical Research 2023;9(1):1–4.
29. Hassani H, Silva ES. The role of ChatGPT in data science: how ai-assisted conversational interfaces are revolutionizing the field. Big data and cognitive computing 2023;7(2):62.
30. Liang C, Karolus J, Kosch T, Schmidt A. On the Suitability of Real-Time Assessment of Programming Proficiency Using Gaze Properties. In: Proceedings of the 7th ACM International Symposium on Pervasive Displays PerDis '18, New York, NY, USA: Association for Computing Machinery; 2018. .
31. Wratten L, Wilm A, Göke J. Reproducible, scalable, and shareable analysis pipelines with bioinformatics workflow managers. Nature methods 2021;18(10):1161–1168.
32. Ahmad F, Ahmad W. An efficient astronomical image processing technique using advance dynamic workflow scheduler in cloud environment. International Journal of Information Technology 2022;14(6):2779–2791.
33. Kunkel JM, Pedro LR. Potential of I/O aware workflows in climate and weather. Supercomputing Frontiers and Innovations 2020;7(2):35–53.
34. Lehmann F, Frantz D, Becker S, Leser U, Hostert P. FORCE on Nextflow: Scalable Analysis of Earth Observation Data on Commodity Clusters. In: CIKM Workshops; 2021. .
35. Yu J, Buyya R. A taxonomy of scientific workflow systems for grid computing. ACM Sigmod Record 2005;34(3):44–49.
36. Liew CS, Atkinson MP, Galea M, Ang TF, Martin P, Hemert JIV. Scientific workflows: moving across paradigms. ACM Computing Surveys (CSUR) 2016;49(4):1–39.
37. Gil Y, Deelman E, Ellisman M, Fahringer T, Fox G, Gannon D, et al. Examining the challenges of scientific workflows. Computer 2007;40(12):24–32.
38. Harenslak BP, de Ruiter J. Data Pipelines with Apache Airflow. Simon and Schuster; 2021.
39. Goecks J, Nekrutenko A, Taylor J, team@ galaxyproject org GT. Galaxy: a comprehensive approach for supporting accessible, reproducible, and transparent computational research in the life sciences. Genome biology 2010;11:1–13.
40. Di Tommaso P, Chatzou M, Floden EW, Barja PP, Palumbo E, Notredame C. Nextflow enables reproducible computational workflows. Nature biotechnology 2017;35(4):316–319.
41. Deelman E, Vahi K, Juve G, Rynge M, Callaghan S, Maechling PJ, et al. Pegasus, a workflow management system for science automation. Future Generation Computer Systems 2015;46:17–35.
42. Köster J, Rahmann S. Snakemake—a scalable bioinformatics workflow engine. Bioinformatics 2012;28(19):2520–2522.
43. Kenton JDMWC, Toutanova LK. BERT: Pre-training of Deep Bidirectional Transformers for Language Understanding. In: Proceedings of NAACL-HLT; 2019. p. 4171–4186.
44. Brown T, Mann B, Ryder N, Subbiah M, Kaplan JD, Dhariwal P, et al. Language models are few-shot learners. Advances in neural information processing systems 2020;33:1877–1901.
45. Anil R, Dai AM, Firat O, Johnson M, Lepikhin D, Passos A, et al. Palm 2 technical report. arXiv preprint arXiv:230510403 2023;.
46. Thoppilan R, De Freitas D, Hall J, Shazeer N, Kulshreshtha A, Cheng HT, et al. Lamda: Language models for dialog applications. arXiv preprint arXiv:220108239 2022;.
47. Yuan A, Coenen A, Reif E, Ippolito D. Wordcraft: story writing with large language models. In: 27th International Conference on Intelligent User Interfaces; 2022. p. 841–852.
48. Petridis S, Diakopoulos N, Crowston K, Hansen M, Henderson K, Jastrzebski S, et al. Anglekindling: Supporting journalistic angle ideation with large language models. In: Proceedings of the 2023 CHI Conference on Human Factors in Computing Systems; 2023. p. 1–16.
49. Jiang E, Olson K, Toh E, Molina A, Donsbach A, Terry M, et al. Promptmaker: Prompt-based prototyping with large language models. In: CHI Conference on Human Factors in Computing Systems Extended Abstracts; 2022. p. 1–8.
50. Wang B, Li G, Li Y. Enabling conversational interaction with mobile ui using large language models. In: Proceedings of the 2023 CHI Conference on Human Factors in Computing Systems; 2023. p. 1–17.
51. Wang S, Petridis S, Kwon T, Ma X, Chilton LB. PopBlends: Strategies for conceptual blending with large language models. In: Proceedings of the 2023 CHI Conference on Human Factors in Computing Systems; 2023. p. 1–19.
52. Osone H, Lu JL, Ochiai Y. BunCho: ai supported story co-creation via unsupervised multitask learning to increase writers' creativity in japanese. In: Extended Abstracts of the 2021 CHI Conference on Human Factors in Computing Systems; 2021. p. 1–10.
53. Wang C, Liu X, Song D. Language models are open knowledge graphs. arXiv preprint arXiv:201011967 2020;.
54. Manakul P, Liusie A, Gales MJ. Selfcheckgpt: Zero-resource black-box hallucination detection for generative large language models. arXiv preprint arXiv:230308896 2023;.
55. Peng B, Galley M, He P, Cheng H, Xie Y, Hu Y, et al. Check your facts and try again: Improving large language models with external knowledge and automated feedback. arXiv preprint

arXiv:230212813 2023;.
56. Chen M, Tworek J, Jun H, Yuan Q, Pinto HPdO, Kaplan J, et al. Evaluating large language models trained on code. arXiv preprint arXiv:210703374 2021;.
57. Clement C, Drain D, Timcheck J, Svyatkovskiy A, Sundaresan N. PyMT5: multi-mode translation of natural language and Python code with transformers. In: Proceedings of the 2020 Conference on Empirical Methods in Natural Language Processing (EMNLP); 2020. p. 9052–9065.
58. Li Y, Choi D, Chung J, Kushman N, Schrittwieser J, Leblond R, et al. Competition-level code generation with alphacode. Science 2022;378(6624):1092–1097.
59. Le H, Wang Y, Gotmare AD, Savarese S, Hoi SCH. Coderl: Mastering code generation through pretrained models and deep reinforcement learning. Advances in Neural Information Processing Systems 2022;35:21314–21328.
60. Jain N, Vaidyanath S, Iyer A, Natarajan N, Parthasarathy S, Rajamani S, et al. Jigsaw: Large language models meet program synthesis. In: Proceedings of the 44th International Conference on Software Engineering; 2022. p. 1219–1231.
61. Jiang E, Toh E, Molina A, Olson K, Kayacik C, Donsbach A, et al. Discovering the syntax and strategies of natural language programming with generative language models. In: Proceedings of the 2022 CHI Conference on Human Factors in Computing Systems; 2022. p. 1–19.
62. Vaithilingam P, Zhang T, Glassman EL. Expectation vs. experience: Evaluating the usability of code generation tools powered by large language models. In: Chi conference on human factors in computing systems extended abstracts; 2022. p. 1–7.
63. Copilot; 2024. [Online; accessed April 8th 2024]. https://copilot.github.com.
64. Dakhel AM, Majdinasab V, Nikanjam A, Khomh F, Desmarais MC, Jiang ZMJ. Github copilot ai pair programmer: Asset or liability? Journal of Systems and Software 2023;203:111734.
65. Kazemitabaar M, Chow J, Ma CKT, Ericson BJ, Weintrop D, Grossman T. Studying the effect of AI Code Generators on Supporting Novice Learners in Introductory Programming. In: Proceedings of the 2023 CHI Conference on Human Factors in Computing Systems; 2023. p. 1–23.
66. Maddigan P, Susnjak T. Chat2vis: Generating data visualisations via natural language using chatgpt, codex and gpt-3 large language models. IEEE Access 2023;.
67. Hassan MM, Knipper A, Santu SKK. ChatGPT as your Personal Data Scientist. arXiv preprint arXiv:230513657 2023;.
68. Zahra Z, Li Z, Filgueira R. Laminar: A New Serverless Stream-based Framework with Semantic Code Search and Code Completion. arXiv preprint arXiv:230900584 2023;.
69. Pedregosa F, Varoquaux G, Gramfort A, Michel V, Thirion B, Grisel O, et al. Scikit-learn: Machine learning in Python. the Journal of machine Learning research 2011;12:2825–2830.
70. ChatGPT; 2024. [Online; accessed April 8th 2024]. https://chat.openai.com.
71. Sänger M, Mecquenem ND, Lewińska KE, Bountris V, Lehmann F, Leser U, et al. Supporting data for "A Qualitative Assessment of Using ChatGPT as Large Language Model for Scientific Workflow Development". GigaScience Database;https://doi.org/10.5524/102522.
72. Sedgwick P, Greenwood N. Understanding the Hawthorne effect. Bmj 2015;351.
73. Sanvicente-García M, García-Valiente A, Jouide S, Jaraba-Wallace J, Bautista E, Escobosa M, et al. CRISPR-Analytics (CRISPR-A): A platform for precise analytics and simulations for gene editing. PLOS Computational Biology 2023;19(5):e1011137.
74. CRISPR-Cas9 Workflow; 2024. [Online; accessed May 10th 2024]. https://archive.softwareheritage.org/swh:1:dir:b2780fbecc6eab9ba8593df8c48ebe33586bbffe;origin=https://github.com/nf-core/crisprseq.
75. RS1 Star Workflow; 2024. [Online; accessed May 10th 2024]. https://archive.softwareheritage.org/swh:1:dir:b6dcc4c708dd465f40afcdc17dd0032820584edb;origin=https://github.com/Nine-s/nextflow_RS1_star.
76. FORCE2NXF-Rangeland Workflow; 2024. [Online; accessed May 10th 2024]. https://archive.softwareheritage.org/swh:1:dir:5005b177c9fc7621569865b83948ab194898bd8a;origin=https://github.com/CRC-FONDA/FORCE2NXF-Rangeland.
77. Trends Workflow; 2024. [Online; accessed May 10th 2024]. https://archive.softwareheritage.org/swh:1:dir:61ebb2546fc58f2f241d5d8239aedefe92f8282c;origin=https://github.com/kelewinska/FONDA_trends-nf.
78. FONDA Airflow Dags; 2024. [Online; accessed May 10th 2024]. https://archive.softwareheritage.org/swh:1:dir:31e78a3e58b30df63d2200fee4f5435a799e9e62;origin=https://github.com/CRC-FONDA/fonda-airflow-dags.
79. Sudmanns M, Tiede D, Lang S, Bergstedt H, Trost G, Augustin H, et al. Big Earth data: disruptive changes in Earth observation data management and analysis? International Journal of Digital Earth 2020;13(7):832–850.
80. Framework for Operational Radiometric Correction for Environmental monitoring; 2024. [Online; accessed April 8th 2024]. https://github.com/davidfrantz/force.
81. Nextflow; 2024. [Online; accessed April 8th 2024]. https://www.nextflow.io.
82. Apache Airflow; 2024. [Online; accessed April 8th 2024]. https://airflow.apache.org.
83. Lewińska KE, Buchner J, Bleyhl B, Hostert P, Yin H, Kuemmerle T, et al. Changes in the grasslands of the Caucasus based on cumulative endmember fractions from the full 1987–2019 landsat record. Science of Remote Sensing 2021;4:100035.
84. Lewińska KE, Hostert P, Buchner J, Bleyhl B, Radeloff VC. Short-term vegetation loss versus decadal degradation of grasslands in the Caucasus based on Cumulative Endmember Fractions. Remote Sensing of Environment 2020;248:111969.
85. White J, Fu Q, Hays S, Sandborn M, Olea C, Gilbert H, et al. A prompt pattern catalog to enhance prompt engineering with chatgpt. arXiv preprint arXiv:230211382 2023;.
86. Oinn T, Addis M, Ferris J, Marvin D, Senger M, Greenwood M, et al. Taverna: a tool for the composition and enactment of bioinformatics workflows. Bioinformatics 2004;20(17):3045–3054.
87. Van Dijk EL, Auger H, Jaszczyszyn Y, Thermes C. Ten years of next-generation sequencing technology. Trends in genetics 2014;30(9):418–426.
88. Hu T, Chitnis N, Monos D, Dinh A. Next-generation sequencing technologies: An overview. Human Immunology 2021;82(11):801–811.
89. Chen S, Zhou Y, Chen Y, Gu J. fastp: an ultra-fast all-in-one FASTQ preprocessor. Bioinformatics 2018;34(17):i884–i890.
90. Dobin A, Davis CA, Schlesinger F, Drenkow J, Zaleski C, Jha S, et al. STAR: ultrafast universal RNA-seq aligner. Bioinformatics 2013;29(1):15–21.
91. Langmead B, Trapnell C, Pop M, Salzberg SL. Ultrafast and memory-efficient alignment of short DNA sequences to the human genome. Genome biology 2009;10(3):1–10.
92. Langmead B, Salzberg SL. Fast gapped-read alignment with Bowtie 2. Nature methods 2012;9(4):357–359.
93. Kim D, Paggi JM, Park C, Bennett C, Salzberg SL. Graph-based genome alignment and genotyping with HISAT2 and HISAT-genotype. Nature biotechnology 2019;37(8):907–915.
94. Bolger AM, Lohse M, Usadel B. Trimmomatic: a flexible trimmer for Illumina sequence data. Bioinformatics 2014;30(15):2114–2120.
95. Martin M. Cutadapt removes adapter sequences from high-throughput sequencing reads. EMBnet journal 2011;17(1):10–

12.
96. Zeyen C, Malburg L, Bergmann R. Adaptation of scientific workflows by means of process-oriented case-based reasoning. In: Case-Based Reasoning Research and Development: 27th International Conference, ICCBR 2019, Otzenhausen, Germany, September 8–12, 2019, Proceedings 27 Springer; 2019. p. 388–403.
97. Apache Airflow; 2024. [Online; accessed April 8th 2024]. https://platform.openai.com/docs/models/gpt-3-5.
98. Github; 2024. [Online; accessed April 8th 2024]. https://github.com/search.
99. Trapnell C, Williams BA, Pertea G, Mortazavi A, Kwan G, Van Baren MJ, et al. Transcript assembly and quantification by RNA-Seq reveals unannotated transcripts and isoform switching during cell differentiation. Nature biotechnology 2010;28(5):511–515.
100. Danecek P, Bonfield JK, Liddle J, Marshall J, Ohan V, Pollard MO, et al. Twelve years of SAMtools and BCFtools. GigaScience 2021 02;10(2). https://doi.org/10.1093/gigascience/giab008, giab008.
101. Google Gemini; 2024. [Online; accessed April 8th 2024]. https://gemini.google.com/app.
102. Rozière B, Gehring J, Gloeckle F, Sootla S, Gat I, Tan XE, et al. Code Llama: Open Foundation Models for Code. arXiv preprint arXiv:230812950 2023;.
103. OpenAI Codex; 2024. [Online; accessed April 8th 2024]. https://openai.com/blog/openai-codex.
104. Kloft AM, Welsch R, Kosch T, Villa S. "AI enhances our performance, I have no doubt this one will do the same": The Placebo Effect Is Robust to Negative Descriptions of AI; 2023. .
105. Kosch T, Welsch R, Chuang L, Schmidt A. The Placebo Effect of Artificial Intelligence in Human-Computer Interaction. ACM Transaction Computer-Human Interaction 2023 jan;29(6).
106. Villa S, Kosch T, Grelka F, Schmidt A, Welsch R. The Placebo Effect of Human Augmentation: Anticipating Cognitive Augmentation Increases Risk-Taking Behavior. Computers in Human Behavior 2023;p. 107787. https://www.sciencedirect.com/science/article/pii/S0747563223001383.
107. Wells JD, Campbell DE, Valacich JS, Featherman M. The Effect of Perceived Novelty on the Adoption of Information Technology Innovations: A Risk/Reward Perspective. Decision Sciences 2010;41(4):813–843. https://onlinelibrary.wiley.com/doi/abs/10.1111/j.1540-5915.2010.00292.x.
108. Bosch E, Welsch R, Ayach T, Katins C, Kosch T. The Illusion of Performance: The Effect of Phantom Display Refresh Rates on User Expectations and Reaction Times. In: Extended Abstracts of the 2024 CHI Conference on Human Factors in Computing Systems CHI EA '24, New York, NY, USA: ACM; 2024. .
109. Wei J, Wang X, Schuurmans D, Bosma M, Xia F, Chi E, et al. Chain-of-thought prompting elicits reasoning in large language models. Advances in Neural Information Processing Systems 2022;35:24824–24837.
110. Besta M, Blach N, Kubicek A, Gerstenberger R, Gianinazzi L, Gajda J, et al. Graph of thoughts: Solving elaborate problems with large language models. arXiv preprint arXiv:230809687 2023;.
111. Chen X, Lin M, Schärli N, Zhou D. Teaching large language models to self-debug. arXiv preprint arXiv:230405128 2023;.
112. Fernando C, Banarse D, Michalewski H, Osindero S, Rocktäschel T. Promptbreeder: Self-referential self-improvement via prompt evolution. arXiv preprint arXiv:230916797 2023;.
113. Cai Z, Chang B, Han W. Human-in-the-Loop through Chain-of-Thought. arXiv preprint arXiv:230607932 2023;.
114. Zhang T, Tham I, Hou Z, Ren J, Zhou L, Xu H, et al. Human-in-the-Loop Schema Induction. arXiv preprint arXiv:230213048 2023;.
115. Crusoe MR, Abeln S, Iosup A, Amstutz P, Chilton J, Tijanić N, et al. Methods included: standardizing computational reuse and portability with the common workflow language. Communications of the ACM 2022;65(6):54–63.
116. Common Workflow Language; 2024. [Online; accessed April 8th 2024]. https://www.commonwl.org.

Manuscript Click here to access/download;Manuscript;manuscript.pdf

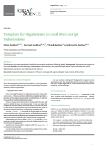

GigaScience, 2024, 1–??

doi: xx.xxxx/xxxx
Manuscript in Preparation
Paper

PAPER

A Qualitative Assessment of Using ChatGPT as Large Language Model for Scientific Workflow Development

Mario Sänger [0000-0002-2950-2587][1,*], Ninon De Mecquenem [0000-0003-3052-6129][1], Katarzyna Ewa Lewińska [0000-0001-6560-1235][2,3], Vasilis Bountris [0000-0002-8682-7302][1], Fabian Lehmann [0000-0003-0520-0792][1], Ulf Leser [0000-0003-2166-9582][1] and Thomas Kosch [0000-0001-6300-9035][1,*]

[1]Department of Computer Science, Humboldt-Universität zu Berlin, 10099 Berlin, Germany and [2]Department of Geography, Humboldt-Universität zu Berlin, 10099 Berlin, Germany and [3]Department of Forest and Wildlife Ecology, University of Wisconsin-Madison, WI 53706, USA

[*]Corresponding authors:{saengema,thomas.kosch}@informatik.hu-berlin.de

Abstract

Background: Scientific workflow systems are increasingly popular for expressing and executing complex data analysis pipelines over large datasets, as they offer reproducibility, dependability, and scalability of analyses by automatic parallelization on large compute clusters. However, implementing workflows is difficult due to the involvement of many black-box tools and the deep infrastructure stack necessary for their execution. Simultaneously, user-supporting tools are rare, and the number of available examples is much lower than in classical programming languages.
Results: To address these challenges, we investigate the efficiency of Large Language Models (LLMs), specifically ChatGPT, to support users when dealing with scientific workflows. We performed three user studies in two scientific domains to evaluate ChatGPT for comprehending, adapting, and extending workflows. Our results indicate that LLMs efficiently interpret workflows but achieve lower performance for exchanging components or purposeful workflow extensions. We characterize their limitations in these challenging scenarios and suggest future research directions.
Conclusions: Our results show a high accuracy for comprehending and explaining scientific workflows while achieving a reduced performance for modifying and extending workflow descriptions. These findings clearly illustrate the need for further research in this area.

Key words: Large Language Models; Scientific Workflows; User Support; ChatGPT

Introduction

Large-scale data analysis pipelines (also known as scientific workflows) are crucial in driving research advances for natural sciences [?]. They are pivotal in accelerating large and complex data analysis on distributed infrastructures and offer essential features, such as reproducibility and dependability [?]. In bioinformatics, for instance, scientific workflows are analyzing the terabyte-large data sets produced by modern DNA or RNA sequencing machines in a wide variety of experiments [?], thereby aiding in building a comprehensive understanding of biological processes and human diseases. Bioinformatics workflows typically include many individual computational steps, such as data pre-processing, extensive quality control, aggregation of raw sequencing data into consensus sequences, machine-learning-based tasks for classification and clustering, statistical assessments, and result visualization. Each

Compiled on: May 11, 2024.
Draft manuscript prepared by the author.

Key Points

- We explore Large Language Models (LLMs) to support users who develop scientific workflows
- We are the first to conduct user studies involving domain experts
- We conduct three studies to assess LLMs in scientific workflow comprehension, adaptation, and extension
- Our results indicate that LLMs efficiently interpret workflows
- Our results show room for improvement regarding component adaptation and workflow extension

step is carried out by a specific program, typically not written by the workflow developer but exchanged within a worldwide community of researchers [?]. Execution of a workflow on a distributed infrastructure, in principle, is taken care of by a workflow engine; however, the idiosyncrasies of the different infrastructures (e.g., file system, number and features of compute nodes, applied resource manager, and scheduler) often require workflow users to tune their scripts individually for every new system [?].

However, typical developers of workflows are researchers from heterogeneous scientific fields who possess expertise in their respective domains but often lack in-depth knowledge in software development or distributed computing. They often encounter difficulties understanding the complex implementations of exchanged codes and the deep infrastructure stack necessary for their distributed execution. This situation challenges efficient workflow implementation, slows down or hinders data exploration and scientific innovation processes [?]. Consequently, low human productivity is a significant bottleneck in the creation, adaption, and interpretation of scientific workflows [?].

Parallel to this, there is a well-established field in human-computer interaction focusing on assisting end-user programmers and software development, as highlighted by previous work [? ? ? ?], to reduce the perceived cognitive workload and improve the overall programmer performance [?]. Research in this field includes programming-by-demonstration [? ?], visual programming [? ?], and natural language instructions [?]. Recent work in this area particularly investigated prospects of general-purpose Large Language Models, such as ChatGPT [?], LLaMA [?] and Bloom[?], for supporting end-user-programming [? ? ?] and software development in general [? ?]. For instance, Bimbatti et al. [?] explore using ChatGPT to enhance natural language understanding within an end-user development environment, assisting non-expert users in developing programs for collaborative robots. Moreover, White et al. [?] introduce prompt design techniques for automating typical software engineering tasks, including ensuring code independence from third-party libraries and generating an API specification from a list of requirements. Surameery et al. [?] evaluate LLMs for supporting code debugging. However, results from such studies, which focus on standard programming languages, cannot easily be transferred to workflow systems. Workflow scripts mostly call external tools with agnostic names and have little recognizable control structures or protected keywords. Publicly available examples are scarce; for instance, the community repository of the popular workflow system Nextflow [?] currently offers only 55 released workflows [?]. Furthermore, workflows can only be understood when the distributed system underlying their execution is considered, creating dependencies much different than usual programs. Moreover, studies investigating how LLMs support users in data science - the field in which workflows are applied extensively - do not address the unique characteristics of scientific workflows either and are limited to theoretical considerations [? ?]. Practical studies, especially those involving real users, are badly missing.

In this work, we address these shortcomings by describing three user-studies in two different scientific fields (biomedicine and Earth observation) that evaluate the suitability of ChatGPT for comprehending, modifying, and extending scientific workflows. Specifically, we evaluate the correctness of ChatGPT regarding explainability, exchange of software components, and extension when providing real-world scientific workflow scripts. Our results show a high accuracy for comprehending and explaining scientific workflows but reduced performance for modifying and extending workflow scripts. The domain experts positively assessed the explainability in qualitative inquiries, emphasizing the time-saving capabilities of using Large Language Models while engineering existing workflows. Overall, our work indicates that general-purpose LLMs have the potential to improve human performance when analyzing complex scientific workflows.

Background

Previous research investigated how related domains, such as programming, can be augmented using interactive technologies [? ?]. In contrast to programming, where applications use a single programming language and are often executed on a single system, scientific workflows combine multiple software artifacts on distributed stacks for advanced data processing. We ground the reader by providing a literature review about scientific workflows and introducing large language models, including their utility to facilitate the creation of software artifacts.

Scientific Workflows

Scientific workflows are widely used by diverse research communities, such as biomedicine [?], astronomy [?], climatology [?], and Earth observation [?] to manage the dataflow and distributed execution of complex analyses, simulations, and experiments. A scientific workflow comprises a series of interconnected computational steps, often with diverse patterns of dependencies, that define how to process and analyze data to reach a particular research objective. Scientific workflows can be regarded as directed acyclic graphs in which the nodes represent computational tasks or operations and edges model dependencies or dataflow between these tasks. An edge from one node to another signifies that the output of the first task is used as input for the second [?]. For example, Figure ?? illustrates the computational steps, the tools, and the data flow of a bioinformatics workflow for performing differential gene expression analysis. During workflow execution, a single computation step often involves multiple processes, which are typically executed in a distributed fashion on different machines and batches of the input data, resulting in a much more complex execution graph. Consequently, scientific workflows help facilitate the reproducibility and traceability of data analyses by explicitly outlining the steps and parameters involved [?]. Furthermore, they allow for automation, scaling, and optimization of computational processes, which is especially critical in disciplines dealing with large datasets [?]. The increasing importance of scientific workflows for scientific progress has led to a growing interest in developing more user-friendly tools and methods through the research community. Scientific workflow management systems, like Apache Airflow [?], Galaxy [?], Nextflow [?], Pegasus [?], and Snakemake [?], are specifically developed to support users in designing

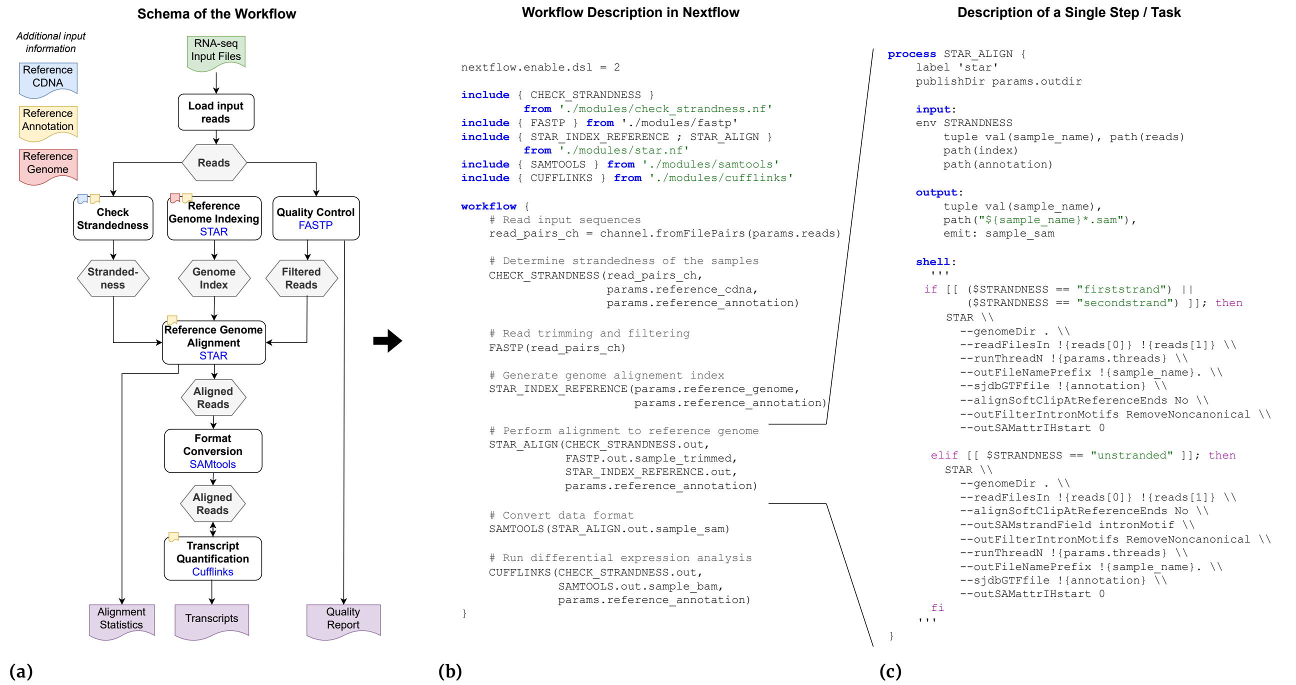

Figure 1. Example bioinformatics workflow for differential gene expression analysis created by a domain expert recruited in our study. The figure highlights the conceptual schema of the workflow (a), its implementation in Nextflow (b), and the implementation of one single step (c), i.e., reference genome alignment using the STAR tool. The workflow comprises six computational steps in total. For each step the used tool is given in blue below the task name.

and executing scientific workflows in various aspects. Key features of such management systems typically include workflow design and composition, (distributed) workflow execution and scheduling, provenance tracking, recovery and failure handling, and resource management [?]. Figure ?? highlights the implementation of the example workflow as well as a single computational step (see Figure ??), i.e., reference genome alignment using the STAR toolkit in Nextflow.

Scientific workflows are often reused and adopted for complex data analysis [?]. Most of the time, users of scientific workflows are unaware of the workflow's internal functionality and technical details. Instead, users of scientific workflows represent domain experts, such as mathematicians, physicians, or bioinformatics, who are experts in their respective domains but not necessarily in programming and interpreting scientific workflows. Existing scientific workflows were implemented and maintained by persons other than the domain user. This reduces the direct interaction with scientific workflows to a minimum, where domain experts only hand in the input data and evaluate the output data. Consequently, domain experts using a workflow often do not have the knowledge to modify, extend, or interpret the details of scientific workflows.

Large Language Models

Language Models, such as BERT [?], GPT-3 [?], Bloom [?] and PaLM-2 [?], build the foundation of many recent advancements in natural language processing and understanding. These models have billions of parameters and are generally pre-trained on vast sets of texts from the web and other repositories, enabling them to encode syntactic and semantic relationships in human language. As a consequence, generative language models, such as ChatGPT, LLaMA [?], and LAMDA [?], can produce machine-generated high-quality text that is indistinguishable from human writing. These generative capabilities have empowered these models to assist in diverse (creative) writing tasks and have been utilized to facilitate a wide range of interactive language-based applications within the HCI community [? ? ? ? ? ?]. For instance, WordCraft [?] investigate the utilization of Large Language Models to aid fiction writers in tasks ranging from transforming a text to resemble a "Dickensian" style to providing suggestions to combat writer's block. Their findings indicate that writers found such text-generating models beneficial even when the generated text is not perfect. Petridis et al. [?] introduce AngleKindling, an interactive tool that employs Large Language Models to support journalists exploring different angles for reporting on a press release. Their study with twelve professional journalists shows that participants found the system considerably more helpful and less mentally demanding than competitor brainstorming tools. Other applications include prototyping support [?], generation of titles and synopses from keywords [?], conversational interactions with mobile user interfaces [?], and conceptional blending [?].

Next to their text writing capabilities, language models are further known to retain commonsense knowledge within their training data, effectively transforming them into accessible knowledge stores that can be seamlessly queried using natural language prompts. For instance, experiments with BERT [?] highlight that the model performance is competitive with traditional information extraction and open-domain question answering. Furthermore, recent studies show the potential of using ChatGPT for knowledge base construction, inspired by the fact that these language models have been pre-trained on vast internet-scale corpora that encompass diverse knowledge domains [?]. However, it is worth noting that Large Language Models are known to frequently generate hallucinations, which are outputs that, while statistically plausible and seemingly believable, are factually incorrect [? ?].

Using LLMs to Support Programming

The ability to generate new text and to reconstruct existing information makes Large Language Models highly appropriate to support users in software development, as programming often requires not only the creation of novel code segments tailored to current requirements and tasks but also depends on the application of established algorithms, software libraries, and best practices. Ac-

cordingly, a large number of papers investigate Large Language Models specially trained for code generation [? ? ? ?] as well as different approaches leveraging these models to provide interactive programmer support [? ? ? ?]. For instance, Jiang et al. [?] discuss GenLine, a natural language code synthesis tool based on a generative Large Language Model and problem-specific prompts for creating or changing program code. The findings from a user study indicate that the approach can provide valuable support to developers. However, they also encounter several challenges, such as participants finding it difficult to form an accurate mental model of the kinds of requests that the model can reliably translate. Similarly, Vaithilingam et al. [?] conducted a user study with 24 participants evaluating their usage and experiences using the GitHub Copilot [?] code generation model while programming. The authors find that the synthesized code often provided a helpful starting point and saved online searching efforts. However, participants encountered issues with understanding, editing, and debugging code snippets from Copilot, resulting in not necessarily improved task completion times and success rates. These findings align with the results of similar studies [?]. However, a controlled experiment in [?] records a positive effect of code generators when used in introductory programming courses for minors.

The use of generative Large Language Models and code generators has been scarcely explored in scientific data analysis and not yet for scientific workflows. Liu et al. [?] examine the Codex code generator [?] in the context of data analysis in spreadsheets for non-expert end-user programmers. Moreover, several studies investigate the utilization for data visualization [? ?]. For example, the study by Maddigan et al. [?] evaluates the efficiency of ChatGPT, Codex, and GPT-3 in producing scripts to create visualizations based on natural language queries. The studies that have the most overlap with our work regarding the intention to support the design of data analysis pipelines are given by Ubani et al. [?] and Zahra et al. [?]. In the case of the former, ChatGPT is used to build a conversational, natural language-based interface between users and the scikit-learn machine learning framework [?] supporting users in several phases of a machine learning project ranging from initial task formulation to comprehensive result interpretation. For the latter, Laminar, a framework for serverless computing, is proposed, which offers possibilities for code searching, summarization, and completion. However, the framework is solely focused on Python implementations.

Methods

This section describes the study methodology. We begin by outlining about the general study approach. Then, we explain the research process for each experiment. Based on related work and the objectives of our research, we state the following research questions:

- **RQ1:** How performant is ChatGPT for comprehending and explaining scientific workflows?
- **RQ2:** How suitable is ChatGPT in suggesting and applying modifications for scientific workflows?
- **RQ3:** How efficient is ChatGPT in extending scientific workflows?

General Study Design

To answer our research questions, we investigate the capabilities of ChatGPT, a widely-used LLM, to comprehend existing workflow descriptions (cf. Study I), to exchange tools used within a workflow (cf. Study II), and to extend a partially given workflow (cf. Study III) using three distinct user studies. We select these use cases as understanding the data flow and the analysis performed is essential for successfully applying scientific workflows. Moreover, exchanging tools and extending a partially given workflow are common use cases in adapting and reusing existing workflows in the work context of domain scientists [?]. For each study, we specially design conversational prompts simulating the interaction between a user working with workflows and ChatGPT. For our studies, we leverage version GPT-3.5 of ChatGPT [?]. We decided to use GPT-3.5 since it is openly available to the public and allows other researchers to reproduce our investigations without additional incurring costs. Moreover, we provide the chat records in the Supplementary Material of this article via the GigaScience database (GigaDB) [?]. Additionally, we develop distinct questionnaires for evaluating the output of ChatGPT by the domain experts for each study. While conducting a study, we present a brief overview of the study's overall goal and the developed questionnaire to the experts. Subsequently, the experts complete the questionnaire independently without the experimenters' support. This procedure is intended as participants were not pressured by a time limit and could freely allocate their time for the study. Furthermore, we intend to avoid a Hawthorne effect, where participants can provide biased responses due to the presence of observers [?].

Participants

Throughout all experiments, we recruited one expert from bioinformatics and three experts working on Earth observation workflows. Using scientific workflows is common in these two areas. In bioinformatics, scientific workflows are an important tool for enabling the automation and documentation of complex data analysis processes, ensuring reproducibility and transparency in research [?]. In Earth observation, scientific workflows streamline the complex process of acquiring, processing, and analyzing vast amounts of satellite and sensor data, enhancing the efficiency and accuracy of environmental studies [?]. Hence, scientific workflows have become commonplace in these two areas. The professions include postdocs and PhD students working at universities. All participants hold a master's degree in their profession and several years of experience in their domain. All experts are between 25 and 40 years old (two female, two male).

Scientific Workflows

In our study, we consider a total of five different workflows. We summarize the used workflows and their details in Table ??. The workflows are taken from the work context of the recruited experts, given their high degree of familiarity and expertise with them. In bioinformatics, we use two workflows that deal with the analysis of genomic data. First, the *crisprseq* workflow, sourced from the *nf-core* repository [?], a hub for best-practice workflows, focuses on analyzing and evaluating gene editing experiments utilizing CRISPR-Cas9 mechanism for genome engineering. Second, the *RS-STAR* workflow, which was implemented by the recruited domain expert and performs differential gene expression analysis using RNA-seq data. The *FORCE2NXF-Rangeland* and *FORCE* are two implementations of an Earth observation workflow that is concerned with analyzing long-term vegetation dynamics in the Mediterranean using the FORCE toolkit [?], which provides processing routines for satellite image archives. The former is implemented using the Nextflow scientific workflow management system [? ?] and the latter by leveraging Apache Airflow [? ?]. The third Earth observation workflow called *Grasslands*. builds on previous work [? ?] aiming at understanding differences in long-term changes in-ground cover fractions specific to European grasslands depending on the definition of endmembers (i.e., unique spectral signatures of a specific material or ground cover) approximating these fractions.

Table 1. Overview of the used workflows. We examine workflows from two scientific domains, i.e., bioinformatics and Earth observation, and two workflow systems (Nextflow and Apache Airflow). For each workflow, we report the number of (high-level) steps and used tools and in which study the workflow is used.

Domain	Workflow	Description	#Steps	#Tools	Study
Bio-informatics	WF1: crisprseq [?] (*Nextflow*)	A bioinformatics data pipeline for analyzing and evaluating gene editing experiments utilizing CRISPR-Cas9 mechanism for genome engineering. Repository: [?] (created 07/2022)	6	9	I
	WF2: RS-Star (*Nextflow*)	The general aim of this workflow is to perform differential gene expression analysis using RNA-seq data. Repository: [?] (created 11/2021)	5	5	I,II,III
Earth Observation	WF3: FORCE2NXF-Rangeland [?] (*Nextflow*)	This workflow analyzes long-term vegetation dynamics in the Mediterranean using the FORCE-toolkit. Repository: [?] (created 11/2020)	9	8	I
	WF4: Grasslands (*Nextflow*)	This workflow aims at understanding differences in long-term changes (1984-2022) in ground cover fractions specific to European grasslands. Repository: [?] (created 08/2023)	6	3	I,III
	WF5: FORCE (*Apache Airflow*)	This workflow focuses on analyzing long-term vegetation dynamics in the Mediterranean using the FORCE-toolkit. Repository: [?] (created 02/2021)	8	8	I

Large Language Model Prompting

The choice and design of prompts entered into a Large Language Model has a decisive influence on the output quality [?] and, in our case, on the suitability of ChatGPT for workflow development and implementation. Our prompts are organized first to provide the context, often including the workflow script, followed by the specific question or instruction under investigation. Suppose the workflow is divided into sub-workflows, possibly distributed over several files. In that case, we first specify the main workflow and then all sub-workflows and task definitions in the order they occur in the main workflow. In our research's initial stages, we experimented with various alternative prompts for each user study, incrementally modifying and enhancing them in response to the outcomes we received. For example, for Study I (i.e., workflow comprehension), we discovered that ChatGPT tends to describe properties of the workflow language or technical aspects instead of workflow characteristics. Such phenomena could be resolved by adding explicit instructions, e.g., "*do not explain nextflow concepts*". We refer to Section ?? for a detailed discussion of prompt design challenges. We stopped this adjustment process after a few iterations as soon as no more of such artificial artifacts were generated. We have refrained from more extensive prompt engineering since workflow designers are domain experts from diverse fields who cannot be assumed to be specialists in developing and tuning prompts. However, we acknowledge that the choice of wording in our prompts influences the results [?]. We discuss the limitations of our work concerning the chosen study design and prompt strategy selection in more detail in Section ??.

Study I: Workflow Comprehension

In our first study, we investigate the capabilities of ChatGPT in capturing the actual purpose of a workflow. In other words, we are prompting ChatGPT to explain the purpose of a workflow. In this study, we assess ChatGPT's quality in comprehending and explaining a workflow's purpose in a user study involving workflow experts. Understanding the data flow and the analysis performed constitutes an important aspect of the daily work with scientific workflows. On the one hand, workflows are often precisely adapted to individual research questions, which makes it challenging even for other experts from the same domain to understand them. On the other hand, in many research institutions, an increasing number of legacy workflows whose original authors and contributors are no longer available for maintaining and refining the codes require taking over by new team members. Understanding a workflow is usually a prerequisite for adopting and applying a workflow correctly.

Thus, Study I pursues three goals: how well does ChatGPT perform on (a) identifying the domain and the overall objective of the analysis, (b) reporting the individual computation steps, used tools, their needed input data, produced output data, and (c) explaining research questions for which these analyses are helpful given the workflow description. The first two parts of the study have a reconstructive character, whereas the third is more explorative, requiring ChatGPT to reason beyond the given workflow description. We build a set of five different prompts to evaluate ChatGPT's capabilities concerning the three dimensions. When providing the workflow definition in the prompt, delete all comments within the definition to prevent information leakage. Table ?? depicts the developed prompts. For each workflow, all prompts are executed in one conversation, enabling ChatGPT to use the in- and output of previous prompts as context information. We ask the domain scientists to evaluate answers given by ChatGPT using a feedback questionnaire. The complete questionnaire contains nine items in total and can be found in full-length in the article's Supplementary Material (A). The questionnaire focuses on the correctness of the prompts regarding the aim of the workflow, the explanation, and the forecast of addressed research questions. For four of the nine items, users rate the generated explanations on a 5-point Likert scale (i.e., 1: Strongly Disagree; 5 Strongly Agree.). In addition, three items comprise quantitative evaluations of how many computational steps are correctly detected, how many utilized software tools and programs are accurately identified, and how many valid follow-up research questions. The remaining question items concern the quality of the explanations of the workflow sequence, the description of the tools used, and the results produced. We add a comment field for each item to report issues and errors in the generated explanations if the domain expert does not apply the content.

Results

The results of the expert surveys are presented in the following according to the three subcategories of the questionnaire, i.e., research area and the overall aim of the workflow, explanation of workflow details, and subsequent research questions.

Table 2. Overview of the used prompts to investigate ChatGPT's capabilities in capturing the content of a workflow description (Study I). *[workflow-language]* and *[workflow-text]* represent placeholders for the workflow management system, i.e., Nextflow or Apache Airflow, and the workflow description text. All prompts are executed within one conversation.

ID	Category	Prompt
P1_1	Overall aim	The following text contains a scientific workflow written in *[workflow-language]*:<br>*[workflow-text]*<br>Explain from which research area this workflow originates and describe the general aim of this workflow. Don't explain *[workflow-language]* concepts.
P1_2	Workflow explanation	Explain all individual tasks that are implemented in this workflow. For each task explain which software programs or tools are used in this workflow to perform the task. Don't explain *[workflow-language]* concepts.
P1_3	Workflow explanation	Explain the type of input data and the format of the input data needed for this workflow. Don't explain the workflow itself.
P1_4	Workflow explanation	Explain the overall result of this workflow. For each individual task of the workflow report the type of data that is produced by this task.
P1_5	Research questions	Explain up to 3 research questions for which this workflow is helpful.

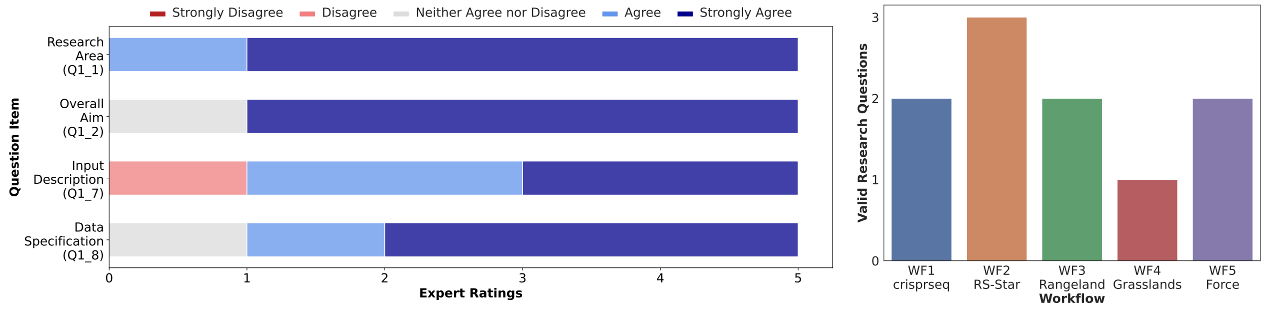

(a) (b)

Figure 2. Results from Study I: (a) rating distribution of the domain experts for ChatGPT's capability in identifying the research area and overall aim as well as the input and data description of a workflow. The question item identifier (see Supplementary Material (A)) is given in parenthesis for each row. (b) Number of valid research questions generated by ChatGPT for the different workflows as assessed by the domain experts (question item *Q1_9*). We prompted ChatGPT to output up to three research questions per workflow.

Overall Aim of the Workflow

The first two rows of Figure ?? highlight the rating distribution of the domain experts concerning ChatGPT's explanations for the research area and the overall aim of the workflows. The experts agreed with the explanations generated, indicating a basic understanding of ChatGPT regarding the different workflows ($\mu = 4.7$, $\sigma = 0.68$). The lowest agreement, with a score of 4 concerning the research area and 3 for the overall aim, was recorded in the evaluation of the *WF4-Grasslands* workflow. In this case, the expert could not agree with the explanations mainly due to the wrong interpretation of an abbreviation within the workflow description, i.e., *FNF* was misinterpreted as *"fraction of non-forest"*, instead of *fold and fill*. This misunderstanding resulted in the workflow being explained as examining forest regions rather than grasslands.

Workflow Explanation

In general, the participants regard the quality of the explanations given by ChatGPT as high (see Figures ?? and ??). All computational steps are accurately identified in three of the five workflow descriptions. Moreover, for four of these five workflows, every tool employed is correctly detected, but in *WF5-Force*, two out of eight were missing. The detailed descriptions of the tasks and tools provided by ChatGPT were also judged to be coherent by the experts. Overall, the worst performance is achieved with the output for workflow *WF4-Grasslands* for which only four out of six tasks are correctly extracted and only most of the tools are correctly described. These errors are mainly due to follow-up errors that result from the incorrect recognition of the workflow purpose.

The results of the questions items (see Q1_7 and Q1_8 in Supplementary Material (A)), which assess the produced information about the format and type of input and output data of ChatGPT, can be seen in the two lower rows in Figure ??. Similar to the previous findings, the information generated for four of the five workflows are evaluated positively ($\mu = 4.2$, $\sigma = 1.0$). Again, only the produced information for the workflow *WF4* was assessed as neutral or negative, i.e., input description score of 2 (disagree) and data specification of 3 (neither agree nor disagree).

Research Questions

The last query was concerned with explaining up to three subsequent research questions to a given workflow. Figure ?? shows the result of this question item. ChatGPT achieves only moderate performance, generating just for one workflow, i.e., *WF2-RS-Star*, three valid research questions. In total, out of the 15 generated research questions 10 were correct. These figures suggest that ChatGPT offers only a reduced performance for more explorative tasks.

Study II: Workflow Modification

The second study investigates how much ChatGPT can aid domain experts in modifying and tailoring a workflow in our second study. Researchers usually do not start from scratch when developing workflows but typically adapt or reuse parts of existing workflows from the community [?]. This strategy applies in particular to biomedicine, in which workflows are more widespread and have

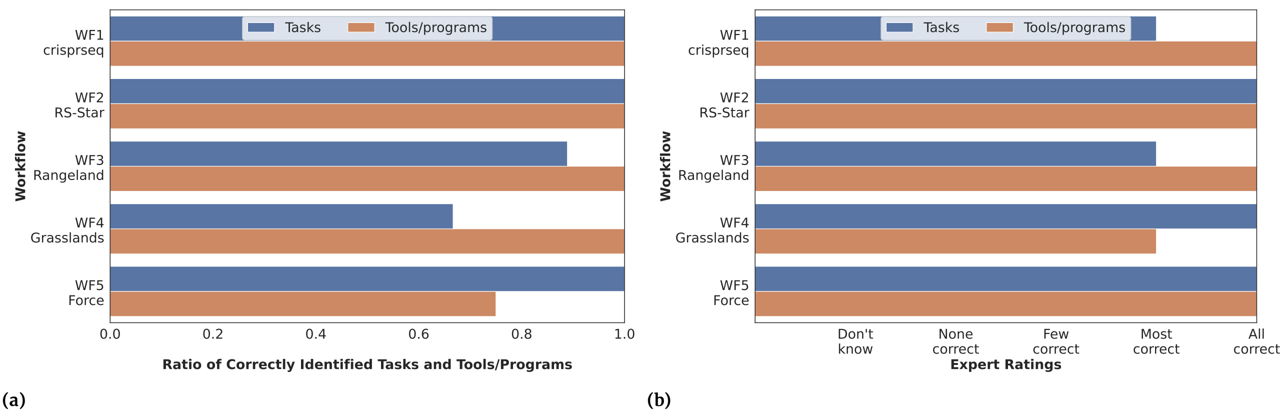

Figure 3. Result statistics highlighting the number of correctly identified tasks and tools of a workflow (a) and their explanation (b). We report separate results per investigated workflow. The results are based on the ratings of the domain experts. Results in (a) correspond to the items *Q1_3* and *Q1_5* and in (b) to *Q1_4* and *Q1_6* of the questionnaire given in the article's Supplementary Material (A).

a longer tradition compared to other domains [?]. For example, genomic workflows will often be applied to a broad spectrum of data originating from different sources, each with its distinctive features and characteristics, making it necessary to adjust the workflow definition for more efficient data processing. Moreover, technological advancements, such as in genome sequencing technology [?], lead to new tools specially developed to leverage the capabilities of the new technologies. The continuous integration of new and alternative scientific tools into existing workflows is essential to conduct state-of-the-art research [?].

In our study, we are particularly investigating the exchange of used tools in the bioinformatics workflow *WF2-RS-Star* whose computational scheme is given in Figure ??. We select two parts of the workflow to be modified:

i. read trimming and filtering (also called read quality control), originally performed by FASTP [?] and
ii. reference genome indexing and alignment, carried out by STAR [?].

For assessing the workflow modification capabilities of ChatGPT, we build four prompts (see Table ??). The first prompt requests a list of alternative tools for a given workflow step from ChatGPT. The second and third prompts request the recommendation of two alternative tools, including an explanation of the suggestion, a comparison of the selected tools with the tool originally used in the workflow script, and their strengths and weaknesses. With the last prompt, the actual rewriting of the workflow to include the selected tool is requested. We test the inclusion of two alternative tools per computational task, i.e., the prompts *P2_3* and *P2_4* (see Table ??) are carried out once for each tool from the recommendation. Analogous to Study I, we use a questionnaire for evaluating ChatGPT's output by the biomedical domain expert containing 13 items in total. For most items (9 out of 13), the generated texts and explanations concerning methodical differences or pros and cons of the tools should be rated on a 5-point Likert scale. The remaining questions require numerical ratings (2 items), yes/no answers (1), and free text fields (1). Again, we add a comment field for each item to report issues and errors in the generated explanations if the domain expert does not fully apply the content. The complete questionnaire is given in the article's Supplementary Material (B). When conducting the study, we also provide the generated workflow scripts to the domain experts and asked them to execute them on their systems. Furthermore, we request the experts to inspect and correct any non-functional scripts. For the latter, we set a time limit of 20 minutes per tool substitution.

Results

The results are presented in the following according to the two subcategories of the prompts, including the exploration of alternative tools and workflow modification. We summarize the results of the two use cases (i.e., read quality control, reference genome alignment) and each of the two alternative tools when reporting the results.

Tool Exploration

When exploring possible alternative tools, ChatGPT showed a good performance, providing a fully valid list of ten alternative tools using prompt *P2_1* (see Table ??) for both scenarios. The generated output list for both tasks can be seen in Figure ??. However, the domain expert criticized the alternative tools for reference genome alignment specified Bowtie [?] and not its successor Bowtie2 [?]. From these, ChatGPT recommended HISAT2 [?] and Bowtie as alternatives for genome alignment and Trimmomatic [?] and Cutadapt [?] for read quality control. According to the domain expert, all tools are principally valid substitutions, but Bowtie is a rather inappropriate suggestion since it is not specialized for RNAseq data. The results of explaining the tools' methodical differences and strengths and weaknesses can be seen in Figure ??. Essentially, the generated textual explanations were assessed positively, except when describing the methodological differences between the tools. In this case, ChatGPT could offer a convincing explanation in only 50% of the cases. In the other cases, the texts were too general, and differences were not named clearly. In summary, similar to the results from Study I, ChatGPT solves these rather reconstructive tasks well, reaching an average score of 4.1 (min=3, max=5, σ=0.81) when taking the results from items Q2_3 to Q2_7 (see questionnaire in Supplementary Material (B)) into account. These results indicate that ChatGPT could effectively used to explore tools in a given field.

Workflow Modification

We requested ChatGPT to re-write the workflow script for each of the two recommended alternative tools in both use cases, resulting in four modified workflows in total. Table ?? summarizes the results achieved. First of all, none of the generated workflow scripts was entirely correct. Only in one of the four cases, the generated script was (at least) syntactically valid, i.e., if replacing FASTP with Trimmomatic for read quality control, and the domain expert could execute it without further adaptations. However, the script was not a semantically correct modification of the original workflow script since one particular quality control routine (i.e., PolyG trimming) was not reflected in the adopted script. This issue also occurred for Cutadapt, the other alternative tool for quality control. For both

Alternative Tools for Reference Genome Alignment

ChatGPT Output:

Here is a list of 10 alternative tools that can be used for reference genome alignment:

1. ***Bowtie***: A fast and memory-efficient aligner for short DNA sequences.
2. ***BWA*** (Burrows-Wheeler Aligner): A popular aligner for mapping DNA sequences against a large reference genome.
3. ***HISAT2***: A fast and sensitive aligner for spliced alignment of RNA-seq reads.
4. ***TopHat***: A widely used aligner for RNA-seq reads that incorporates splice junctions.
5. ***Subread***: A versatile aligner capable of aligning both DNA and RNA sequences to a reference genome.
6. ***Salmon***: A lightweight aligner specifically designed for accurate quantification of transcript abundance from RNA-seq data.
7. ***STAR*** (Spliced Transcripts Alignment to a Reference): The tool used in the provided workflow, known for its speed and ability to handle splice junctions.
8. ***GSNAP***: A general-purpose aligner that allows for highly sensitive and accurate alignment of short reads against a reference genome.
9. ***Novoalign***: A versatile aligner that supports both DNA and RNA sequence alignment, offering high sensitivity and accuracy.
10. ***Segemehl***: A read mapper optimized for mapping short reads against large genomes with a focus on finding splice junctions accurately.

These are just a few examples of the many alignment tools available in the field of bioinformatics. The choice of tool depends on specific requirements, such as the nature of the data (DNA or RNA), read length, presence of splice junctions, and desired performance characteristics.

Alternative Tools for Read Filtering and Trimming

ChatGPT Output:

Certainly! Here's a list of 10 alternative tools that can be used for read filtering and trimming in biomedical workflows:

1. ***Trimmomatic***: A popular tool for read trimming, adapter removal, and quality filtering.
2. ***Cutadapt***: Used for adapter trimming in high-throughput sequencing data.
3. ***BBDuk***: Part of the BBMap package, it offers read trimming, filtering, and quality control.
4. ***SeqKit***: A fast and lightweight tool for sequence processing, including read trimming.
5. ***Trimgalore***: Specifically designed for trimming and quality filtering of Illumina sequencing data.
6. ***Sickle***: A tool that focuses on quality-based trimming of reads from high-throughput sequencing data.
7. ***AlienTrimmer***: A read trimming tool that uses a statistical approach to identify and remove adapters.
8. ***Prinseq***: Provides various options for read filtering, trimming, and quality control.
9. ***SolexaQA***: Offers quality assessment, read trimming, and error correction for Illumina sequencing data.
10. ***AdapterRemoval***: Specialized for adapter trimming and quality filtering in paired-end sequencing data.

These tools provide different features and algorithms for read filtering and trimming, allowing researchers to choose the one that best suits their specific needs and preferences.

Figure 4. Representation of the output of ChatGPT when requested to provide a list of alternative tools for reference genome indexing and alignment (left) and read quality control (right) using prompt P2_1. All tools are assessed to be valid by the biomedical domain expert.

tools, ChatGPT reported in the explanation text that the tools do not support this feature; however, in reality, they do. This failure could be interpreted as a kind of LLM hallucination [?]. The second task, reference genome indexing and alignment, revealed different issues than the first. Here, the main problem was the correct linking of the two sub-parts of the task, first the index generation and then the computation of the actual alignment. For the former, each tool specifies and uses its distinct data format and defines how to store the index (e.g., saving it in one or multiple files). However, the storing strategy also affects how the output of the indexing task has to be passed on to the input of the alignment computation. In the scripts generated by ChatGPT, the actual step descriptions to invoke indexing and alignment by the tools were (generally) valid. However, the linking of these two needed to be corrected. For example, Bowtie saves its index in multiple files sharing a common file name prefix, which has to be specified as a parameter during alignment. However, in the modified script, the list of all files of a specially created directory was passed to the alignment process. For Bowtie, this problem could be easily fixed by the domain expert, but for HISAT2, it was not that trivial and hence could not be solved in the given time budget of 20 minutes.

To sum up, the study's results indicate that modifying workflow scripts poses considerable challenges for ChatGPT as it requires a detailed understanding of the tool's idiosyncrasy, the exact computations they perform, and the data formats they use.

Study III: Workflow Extension

In the third study, we investigate the capabilities of ChatGPT in extending a scientific workflow given a partial script. As discussed in the motivation for Study II (see Section ??), users often reuse parts of existing workflows from the research community and adapt them to the research question at hand by enhancing the pipeline with additional analyses and computational steps [?]. Moreover, data analysis projects are often exploratory processes, and computation pipelines are incrementally adapted and extended based on executions and findings from previous versions of the workflow, e.g., to include additional data correctness checks, add more differentiated result evaluations and provide advanced result visualizations [?]. In our study, we simulate this incremental exploration process by taking an existing workflow and removing *n* steps at the end of it. We then request ChatGPT to a) enumerate the necessary steps to accomplish the original research goal and b) regenerate the next step using the tool of the original pipeline or by giving a verbal description of the task. For this study, we select one workflow from each research domain for investigation: *WF2-RS-Star* for biomedicine and *WF4-Grasslands* for earth observation. The two workflows were chosen because they offer different implementation characteristics, i.e., *WF2-RS-Star* leverages almost exclusively external tools, whereas *Grasslands* relies more strongly on specially implemented R and Python scripts. Moreover, Study I (see Section ??) already showed notable result differences of ChatGPT for both workflows. By choosing these two specific workflows, we aim to encompass a possibly broad spectrum of performance variations. We test ChatGPT's workflow extension capabilities in three scenarios: For WF2-RS-Star, we remove the last step *transcript quantification* as well as the last two steps *transcript quantification* and *format conversion*, forming two extension scenarios. In the case of *WF4-Grasslands*, we remove all steps at the tail of the workflow, including *autoregressive trend analysis* (see schema in Supplementary Material (D)).

Table ?? illustrates the prompts developed for this purpose. This study uses slightly different prompts (see P3_2a and P3_2b) reflecting the different workflow types, i.e., tool- vs. script-based. For the latter, we include additional instructions to a) specify the programming language of the script and b) ask the domain expert for a verbal description of the computational steps to be implemented. See Supplementary Material (E) for the verbal description provided by the earth observation expert. The questionnaire for evaluating the generated outputs consisting of seven items can be found in the article's Supplementary Material (C).

Results

The results are presented in the following according to the two subcategories of the prompts, i.e., workflow exploration and extension.

Table 3. Overview of the used prompts to investigate ChatGPT's capabilities in swapping used tools in bioinformatic workflows (Study II). Information in square brackets specifies placeholders for concrete information regarding the workflow or the tool to be replaced.

ID	Category	Prompt
P2_1	Tool exploration	The following text contains a *[domain]* workflow written in *[workflow-language]*: *[main-workflow]* The following snippets contain the source code for the step of the workflow which uses *[tool]* to perform *[step]*: *[step-source-code]*. Please provide a list of 10 alternative tools to perform *[tool]*.
P2_2	Tool exploration	The following text contains a *[domain]* workflow written in *[workflow-language]*: *[main-workflow]* The following snippets contain the source code for the step of the workflow which uses *[tool]* to perform *[step]*: *[step-source-code]*. Alternative tools for *[step]* are: *[list-of-tools]* Which of the tools would you recommend as most suitable alternative for *[step]* in the given workflow. Please name the two alternatives and give an explanation why these tools are especially advisable for the given workflow.
P2_3	Tool exploration	*[original-tool]* and *[alternative-tool]* are two tools for *[step]* in *[domain]* workflows. First, explain the differences between the tools and the underlying approaches. Second, name strenghts and weaknesses of both tools.
P2_4	Workflow modification	The following text contains a *[domain]* workflow written in *[workflow-language]*: *[main-workflow]* The following snippets contain the source code for the step of the workflow which uses the *[tool]* to perform *[step]*: *[step-source-code]* Please re-write the code of the workflow and the proccess to use *[alternative-tool]* instead of *[original-tool]*. The number of parameters of the individual process descriptions may have to be adjusted. Please explain features / options of *[original-tool]* which are not supported in *[new-tool]*.

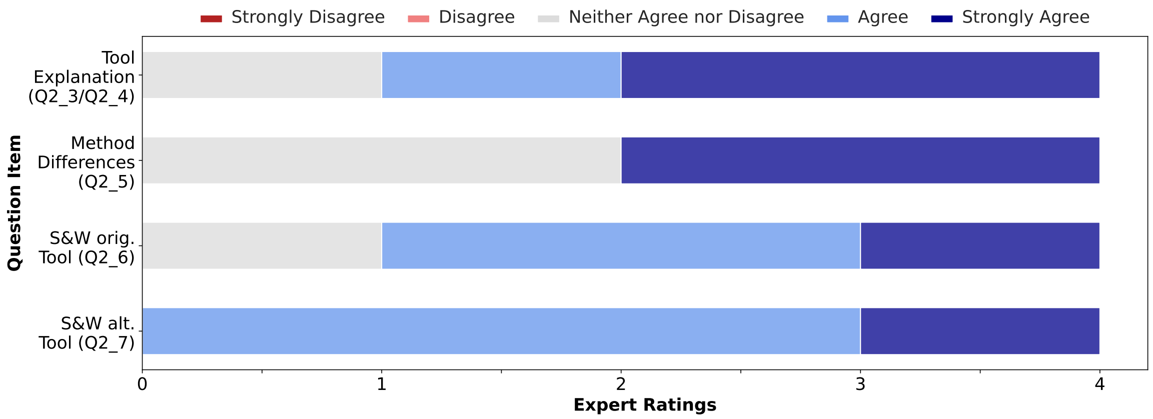

Figure 5. Overview of the rating distribution of the biomedical domain expert for ChatGPT's capability for explaining alternative tools, methodical differences, and strengths and weaknesses (S&W) of the tools. The question item identifier (see Supplementary Material (B)) is given in parenthesis for each row.

Workflow exploration

For describing further computational steps necessary to accomplish a specific research goal given a partial workflow, ChatGPT showed mixed results. The LLM provides a correct list of suitable steps in two of the three scenarios. Also, the tools and methods for implementing the steps suggested by ChatGPT were valid. However, both domain experts criticize that the specifications for the necessary steps and the proposed tools tend to be rather generic and generalized. For instance, for extending the *WF4-Grasslands* workflow the earth observation expert commented:

> *Overall, the proposed workflow is very generic and does not provide a clear roadmap for the analyses. It also proposes to use very simplistic and often imperfect approaches.*

Overall, the results confirm the findings from the two previous studies that ChatGPT shows weaknesses in more exploratory tasks.

Workflow extension

Using the prompts P3_2a and P3_2b (see Table ??), we request ChatGPT to re-construct the last removed computational step in each extension scenario. Table ?? summarizes the results achieved. Like the results from Study II, ChatGPT shows considerable weaknesses in the automatic extension of workflows. None of the generated workflow scripts was executable without the intervention of the domain expert. A clear difference is revealed when comparing the two domains, biomedicine and earth observation. In the former case, the generated workflow scripts are (at least) of such a quality that the domain expert could successfully correct them within 20 minutes. In the generated scripts, mainly syntactical errors occurred (e.g., incorrect usage of variable identifiers, incomplete input definitions, or missing specification of parameters), which could be easily corrected. However, the calls to the respective programs to perform the two tasks were correct.

In contrast, the generated extension for *WF4-Grasslands* was of considerably lower quality. In this case, several syntactic and semantic errors occurred, e.g., the script uses a non-existing library function, no parallelization code is included, and not all requested computations are performed. In this state, the domain expert could not resolve the large number of problems within 20 minutes. However, when interpreting these results, one must remember that the task in this scenario is also significantly more difficult. Instead of a short task description and specification of a tool to be used, ChatGPT has to design and generate the source code for a complex

data analysis procedure containing multiple sub-steps.

Discussion

We conducted three studies to investigate the capabilities of using ChatGPT for comprehending, modifying, and extending scientific workflows. We discuss our methodology and the results in the following.

Comprehending Scientific Workflows

Study I was designed to answer **RQ1** by evaluating ChatGPT's performance in comprehending existing workflows. The domain experts assessed that ChatGPT is good at this task while showing slight differences between the investigated research domains. In particular, the explanations for workflow *WF4-Grasslands* revealed considerable performance drops. Unlike the other workflows investigated, this one uses multiple proprietary R and Python scripts instead of leveraging external tools for assembling data processing pipelines. The lack of standardized tools makes workflow comprehension more challenging since ChatGPT has to interpret complex processing logic and has fewer possibilities to leverage static information, like the description of the general purpose of an established bioinformatics tool, seen through its training while generating the response. In addition, code quality and its readability may strongly influence the results for workflows containing proprietary scripts. For instance, one major problem while explaining *WF4-Grass-lands* in Study I was the misinterpretation of the abbreviation *"fnf"* as *"fraction of non-forest"*, instead of *"fold and fill"*. Such customized and ambiguous terms challenge Large Language Models and reduce their applicability.

In an ablation experiment, we replaced all abbreviations in WF4 with their complete form and re-generated the explanations of prompt P1_1, requesting the overall purpose of the workflow. Having access to the full forms in the script, ChatGPT's output was much better, even if the exact goal of the workflow was still only met on a rather abstract level:

> *[...] aimed at extracting detailed information about land cover dynamics, vegetation phenology, and environmental changes in a specific region using satellite data.*

We also did a reverse experiment using *WF2-RS-Star* by replacing all full-form task names with abbreviations in the workflow script (but keeping the name of the tools called fixed). In this case, the results did not change considerably, highlighting the stronger robustness of ChatGPT against such changes.

Modifying Scientific Workflows

We answer **RQ2** in Study II by evaluating the modification performance of ChatGPT. To this end, we requested the LLM to substitute the leveraged tools for two computational tasks, read quality control and reference genome alignment, in the biomedical workflow *WF2-RS-Star*. The study results suggest that ChatGPT can effectively explore and explain alternative tools in the field, possibly shortening the time the experts spend searching for suitable replacements on the web. In contrast, the results also indicate that ChatGPT rather poorly supports the generation of workflow scripts for using these alternative tools. In only one scenario, i.e., substituting *FASTP* with *Trimmomatic*, the produced script could be run without syntactical errors, and in one other scenario, i.e., replacing STAR with Bowtie, the script could be fixed within 20 minutes to be syntactically and semantically valid. In the used version of ChatGPT and the selected setup, an increase in efficiency cannot be recorded or anticipated, highlighting the need for further research efforts. However, when interpreting the results, it is essential to remember that ChatGPT is a general-purpose LLM rather focusing on human language. A potential option for improvement could be testing generative models more strongly adapted to programming code, such as GitHub Copilot or Code Llama [?]. Moreover, alternative prompting strategies, which adapt the workflow iteratively, could help avoid errors, e.g., when linking the source code of existing tasks with the new task descriptions. We refer to the discussion of other prompting solutions in Section ??. We emphasize again that workflows and the development of tools in bioinformatics, particularly in the field of genomic analyses, are based on a more extended history and have established more robust and more widely-used software than other scientific fields. Accordingly, it is reasonable to infer that the outcomes, such as for suggesting alternative tools, reflect an upper boundary of quality, suggesting that encountering difficulties is more likely in less explored application areas due to insufficient data availability.

Extending Scientific Workflows

Finally, we investigate **RQ3** by conducting Study III. To this end, we requested ChatGPT to extend an existing (partially given) workflow to achieve specific goals. The study results confirm the findings from the two previous studies and emphasize ChatGPT's difficulties in solving more complex and exploratory problems. In this case, explaining the necessary steps to answer the given research questions and the generation of the workflow script for the next step offered (partly) severe issues. Similar to the results of Study I, the picture is mixed regarding the different research domains, earth observation and bioinformatics. For the latter, the generated scripts form a relatively good basis for the implementation, having only (minor) syntactical issues that the expert could quickly fix. In contrast, in the case of earth observation, the script quality was considerably worse, hindering a fast correction by the expert. These results imply that efficient user support is possible for pipelines mainly leveraging external tools. However, further research is necessary to investigate user-support strategies for workflows applying specially implemented analysis scripts. Specifically, human-in-the-loop approaches that involve human experts more closely in the code generation process could also be helpful here (see Section ??).

Scientific Workflows in Large Language Model Training Data

Large Language Models are trained on large amounts of textual data from the web, including programming code and workflow scripts [?]. Therefore, it is crucial to consider whether and to what extent an Large Language Model was already able to access the workflow scripts of our study during its pretraining. According to public information [?], ChatGPT was trained on data gathered until September 2021, meaning that initial versions of two of the five tested workflows (i.e., *WF3-FORCE2NXF-Rangeland* and *WF5-Force*) could have been part of the LLM's training routine. However, the specific training dataset used for ChatGPT is not accessible to the public, preventing a conclusive assessment. To attain a more precise estimate of the potential number of workflow scripts within the training data in general, we initiated searches for scientific workflow repositories on GitHub. We leverage the repository search engine of the website [?] and use the names of four widely-used workflow management systems, i.e., Apache Airflow, Nextflow, Snakemake, and Taverna as a query term. We filter all repositories with creation data less than 2021-09-01 from the query results. Of course, the results must be interpreted carefully since not every repository containing the name has to deal with scientific workflows, even if the names are very peculiar. Detailed statistics from our search results can be found in this article's Supplementary Material (F). As of 09/2021, there were between 352 and 1,900 repositories containing one of the workflow system names in their description. Moreover, the

Table 4. Overview of the used prompts to investigate ChatGPT's capabilities in extending a given partial workflow (Study III). We distinguish two types of prompts: workflow exploration and workflow extension. For the latter, we developed two variants specially designed for tool- (P3_2a) and script-based workflows (P3_2b).

ID	Category	Prompt
P3_1	Workflow Exploration	The following text contains a *[domain]* workflow written in Nextflow: *[workflow-description]* The workflow should be used to *[overall-goal]*. Which steps are missing in order to perform *[overall-goal]*? Please specify only the absolutely necessary steps. For each step name up to three *[domain]* tools that can be used to perform the task.
P3_2a	Workflow Extension	The following text contains a *[domain]* workflow written in Nextflow: *[workflow-description]* Please extend to the given workflow to include one further step which *[step-description]* using *[tool]*. Please specify the new process description in a file at *[file-name]*. Please use version 2 of the Nextflow workflow language. The new process should take the output of *[predecessor-step]* as input.
P3_2b	Workflow Extension	The following text contains a *[domain]* workflow written in Nextflow: *[workflow-description]* Please extend to the given workflow to include one further task which performs *[step]* using an *[programming-language]* script. For this, please generate an *[programming-language]* script, stored in *[script-file-name]*, which performs the following computations: *[verbal-task-description]* Next to the *[programming-language]* script generate the Nextflow process description in a file named *[process-file-name]* and the updated workflow. Please use version 2 of the Nextflow workflow language. The new process should take the output of *[predecessor-step]* as input.

results highlight the increasing popularity of workflows since, for all systems except for Taverna, the number of repositories has almost doubled over the last two years. We also checked the number of Nextflow pipelines available in nf-core. As of September 2021, 35 pipe-lines were published, and 19 were under development [?]. Today, nf-core hosts 55 published pipelines and 33 in development. In summary, we can hypothesize from these results that ChatGPT can likely rely only on a relatively small base of workflow scripts during its training compared to classical programming code (e.g., GitHub currently hosts over 3.9 million Java and over 2.2 million Python repositories, which we determined by using GitHub repository search and the search queries "*language:Java*" and "*language:Python*", making user support for workflow design and implementation particularly challenging.

Prompt Design Challenges

While creating prompts for the studies, we identified several challenges and issues that arose while interacting with ChatGPT.

Representation of Workflows

For the representation of the workflow scripts, there is no straightforward option on how to include them in a prompt. The workflow descriptions are often spread over several files containing subworkflows and task descriptions. In our approach, we first specify the main workflow and then all sub-workflows and task descriptions in order of occurrence. However, there might be other, more efficient prompt solutions (with respect to the generative language model). Furthermore, the workflow scripts might exceed the maximum allowed input length of the language model, e.g., ChatGPT variants allow only for 4K to 16K words / tokens [?] in the input sequence. In particular, workflows heavily relying on specially implemented scripts having hundreds of code lines will face this issue.

Loss of Focus

Some of the prompts are very long due to the specification of the entire workflow script, which challenges ChatGPT to maintain focus. Adding additional instructions to the prompt helped to avoid or reduce this phenomenon, e.g. for the explanation use case (Study I) we added to the prompt *"Don't explain nextflow concepts"* (see P1_1 and P1_2 in Table ??) and *"Don't explain the workflow itself"* (P1_3) to prevent ChatGPT to generate outputs describing features of the workflow management system or the complete workflow when requesting input data specification.

Technological Details

In some cases, adaptation to technological details of the specified workflows were necessary. For example, the Nextflow system offers two language versions for describing processing pipelines. The Nextflow workflows in our study all used the new version of the language. However, when extending workflows in Study III, we had to specify the desired version (see P3_2 in Table ??) to get the correct output. This observation is surprising since the partially given workflow is already in the respective version. Interestingly, this was only necessary for the workflow extension but not for their modification (P2_4 in Table ??) in which the phenomena did not occur.

In summary, the efficient and effective formation of prompts offers a wide range of possible solutions. In our study, we identified initial clues and difficulties, but further research is needed to detect further potential for improving the interaction between domain experts and ChatGPT and generative Large Language Models in general.

Limitations and Future Work

In the following, we highlight the limitations of this work that merit further research.

Study Design

In each of our three studies, we created and provided the prompts for testing ChatGPT's capabilities concerning the different use cases and the domain experts only evaluated the outputs of ChatGPT, leading to a rather indirect interaction between the domain scientist and the Large Language Model. An alternative design for the study would be to have the experts interact directly with ChatGPT by developing and refining the prompts independently. In addition to assessing the capabilities of ChatGPT, this would have the advantage of gaining initial insights into interaction forms and patterns of

Table 5. Overview of the results of the workflow modification use case in which the tools performing a specific task are replaced by alternative ones. For each task, we provide the original tool (in parenthesis) and the investigated alternative ones suggested by ChatGPT. For each combination, we highlight (✓=yes, ×=no) whether the generated workflow script could be executed (Exec.), whether it is semantically valid (Val.), and whether it could be fixed within 20 minutes (Fix). For the latter, (✓) indicates cases where the script could be fixed to be executable but not entirely semantically correct. Moreover, we provide excerpts from the domain expert's comments.

Task	Alt. Tool	Exec.	Val.	Fix	Problems / Comments
Read quality control *(FASTP [?])*	Trimmomatic [?]	✓	×	(✓)	• Missing implementation to perform special quality control feature (i.e. PolyG trimming)<br>• Invalid description of workflow differences:<br>- ChatGPT stated that polyG trimming isn't available in Trimmomatic, but it actually is, however extra implementation for data adaptation is needed<br>- Differences of the generated output files not described accurately
	Cutadapt [?]	×	×	(✓)	• Wrong program call: syntactically incorrect specification of two parameters<br>• Missing provision of valid adapter sequences<br>• Invalid description of differences of the workflow script:<br>- ChatGPT stated that polyG trimming isn't available in Cutadapt, but it actually is<br>- Differences of the generated output files not described accurately
Genome indexing & alignment *(STAR [?])*	HISAT2 [?]	×	×	×	• Invalid definition and linkage of in- and output between genome index generation and alignment<br>• Syntactically incorrect call of the alignment process<br>• Does not take parameter *strandedness* of the input data into account<br>• Output files aren't generated correctly
	Bowtie [?]	×	×	✓	• Invalid definition and linkage of in- and output between genome index generation and alignment<br>• Wrong output definition of genome indexing task

the different experts with ChatGPT. Moreover, this would allow for improved customization of the prompts to the particular research domain and the idiosyncratic properties and characteristics of each workflow. Extended optimization of the prompting strategy by the domain scientist could lead to better results but reduce potential time savings in solving the actual task. Our study design was motivated by the fact that the experts had strongly limited time budgets for the study. For example, even for evaluating ChatGPT's outputs in Study I, the experts already needed up to three hours to accurately check the generated explanations. A study design that envisages direct interaction involves high efforts in terms of introduction and explanation to ChatGPT and prompting strategies for the domain scientists, thus limiting the scope of research questions that can be investigated. In addition, the selected study design has the advantage of using the same prompts for the different domains, which contributes to better comparability of the results and eliminates the influence of differences for individual prompt differences.

In our study, we focused solely on ChatGPT as generative language model. However, there are many other general-purpose models available (e.g., PaLM-2 [?], Gemini [?] or Llama-2 [?]) as well as models more specially designed for programming tasks (e.g., GitHub Copilot, Code Llama [?] or OpenAI Codex [?] publicly available and worth investigating. Furthermore, recent research showed that placebo effects can undermine the validity of study results when user expectations are altered through the presence of an AI [? ? ?] (i.e., LLM) or a novel superior technology [? ?] that improves user capabilities. Our studies only highlight the results of ChatGPT in the version used (GPT-3.5) but do not claim generalizability for other Large Language Models. In future work and in the case of using Large Language Models, placebo conditions must be included to avoid findings that are not a result of increased user expectations towards the capabilities of ChatGPT.

Proprietary and Closed-Source LLMs

While ChatGPT and other proprietary LLMs offer remarkable natural language understanding and generation capabilities, they have inherent limitations that can hinder their utility in scientific workflow development. One significant limitation is their lack of transparency in their underlying algorithms and training data, restricting results' direct reproducibility and interpretability. Users typically interact with these models through an application programming interface (API) or web application provided by the company or organization that developed it rather than having direct access to the underlying code or data, which often makes it impossible to trace any changes to the model and, thus, to the results achieved. Moreover, the closed nature of these models restricts researchers' ability to customize or fine-tune them for specific tasks, limiting their adaptability to diverse research domains. An alternative is using freely available, open-source LLMs, like BLOOM [?] or LLaMA [?], where users have more control over model changes. Nonetheless, the data basis and training procedure are often not fully transparent, even with these models. Moreover, it is essential to note that running such models demands substantial computational resources and the corresponding technical expertise.

Prompting Strategy

Next to other models, the prompts used in our studies also constitute a limiting factor. We cannot exclude the possibility that other prompts, using a different structure or wordings, may achieve better results for the investigated use cases. Moreover, in our approach, we used only the workflow description's source code, without comments, to form the prompt. There are several strategies for enriching the prompt with additional context information, which can lead to improved results. For instance, including available documentation artifacts of the workflow, e.g., descriptive texts in the source code repository or the publication introducing (parts of) the workflow, may ease the processing of the text. Similarly, utilizing descriptions or manuals for the tools employed could also contribute to better results.

In addition, the prompts for individual aspects of the workflows could be revised to obtain more differentiated results. For instance, the prompts for exploring alternative tools (see P2_1 and P2_2 in Table ??) could be extended to request further semi-structured information, such as the programming language used, software license, release date, and development history, guiding the tool selection process. Information on the development history, for example, could help domain experts differentiate between well-established, trusted tools and new developments. This would additionally enhance result interpretation since there might be limited information available for recent advancements, potentially leading

Table 6. Overview of the results of the workflow extension use case in which we provide ChatGPT a partial workflow and request the LLM to extend it by one further computational step. For each investigated use case, we highlight (✓=yes, ×=no) whether the generated workflow script could be executed (Exec.), whether it is semantically valid (Val.), and whether it could be fixed within 20 minutes (Fix). For the latter, (✓) indicates cases where the script could be fixed to be executable but not entirely semantically correct. Moreover, we provide excerpts from the domain expert's comments.

Workflow	Task/Tool	Exec.	Val.	Fix	Problems / Comments
WF2 RS-Star	Transcript quantification/ *Cufflinks* [?]	×	×	✓	• Syntax errors: process definition for CUFFLINKS declares one input channel but two were specified<br>• Input tuple does not match input set cardinality declared by process definition<br>• Wrong variable name: sorted_bam (wrong) instead of sample_bam
	Format conversion/ *SAMtools* [?]	×	×	✓	• Syntactical errors: Usage of wrong variable name (sample_sam)<br>• Incorrect syntax for connecting the new task to the previous one SAMTOOLS(STAR_ALIGN.sample_sam) (wrong) vs. SAMTOOLS(STAR_ALIGN.out.sample_sam) (correct)
WF4 Grasslands	AR analysis/ *R script*	×	×	×	• Input to the R script is a path to a directory, not a TIFF file<br>• Incorrect use of remotePARTS library: There is no function called *autoTrend* in this package<br>• Calculation needs to be parallelized (as specified in the request)<br>• Computation should be implemented for four types of inputs, namely: GV, NPV, SOIL, and SHADE.<br>• Desired outputs from the AR model needs to be retrieved and written out (missing)<br>• Script declares a Conda environment (Python), not R environment.

to their (yet) insufficient representation in the LLM.

Furthermore, our approach is based on a linear prompt execution without feedback or revision opportunities. Iterative prompting strategies, such as chain-of-thought [?] or graph-of-thoughts prompting [?], self-debugging [?] or self-adapting approaches [?], can potentially enhance results. These strategies offer dynamic interaction approaches with LLMs, enabling continuous improvement and adaptation of responses through successive refinements. This facilitates, for example, the correction of errors encountered during workflow modification and extension, such as syntax errors in tool invocation, data format mismatches or invalid task linking, by utilizing the LLM output as input for the subsequent iteration. In addition to other technical possibilities of the LLM conversation, prompt customization and adaptation by human experts offer strong potential for improvement [? ?]. Using such human-in-the-loop approaches allows for explicitly addressing the observed problems, especially in workflow modification and expansion, in a semi-automatic process.

Stochastic Generation

In addition to alternative prompting strategies, it should be emphasized that the generated texts are subject to stochastic sampling processes, which can lead to deviations even when reusing the exact same prompts multiple times. In most LLMs, the influence of this phenomenon can be managed using the *temperature* hyperparameter, which regulates how the model samples the next word by adjusting the probability distribution. Higher temperature values soften the distribution, leading to more diverse and creative outputs, allowing the model to explore a broader range of possibilities. On the other hand, a lower temperature tends to produce more conservative and predictable outputs, as the model is more likely to choose tokens with higher probabilities. Thus the parameter serves as a knob to adjust the balance between exploration (generating diverse outputs) and exploitation (producing more likely outputs). An extensive investigation of the parameter's influence represents an interesting future research question.

Limited Number of Domain Experts

In the context of our studies, only four domain experts evaluated the outputs of ChatGPT. In some cases, generated explanations were assessed by one person only (e.g., Study II). This low number of experts limits the validity and generalizability of the results and offers the risk of subjective bias. However, recruitment for such studies is difficult because the number of potential participants is small and they often have strongly limited time budgets, making study design challenging. Please note that for experts in the field, even "just" familiarizing themselves with an unfamiliar workflow is a challenging and time-consuming endeavor.

Investigated domains and selected workflows

Our study explores real-world workflows from the two domains, bioinformatics and earth observation. Of course, these only represent part of the full range of workflows in the natural sciences. It constitutes an exciting follow-up research question: how suitable ChatGPT and other generative Large Language Models are in other research contexts, such as climate research [?] and astronomy [?], and whether it is possible to identify categories or groups of domains which are particularly well (or poorly) supported. Furthermore, we only examined two workflow systems, Nextflow and Apache Airflow, leaving other alternatives, such as Snakemake, Taverna, and Pegasus, for future work. Investigating other workflow systems is especially interesting because they specify different languages with different complexity levels and supported features for designing analysis pipelines. For example, the language of Apache Airflow, which prioritizes flexibility and extensibility through its integration with the general-purpose programming language Python, expands the potential output scope compared to more deterministic languages such as the Common Workflow Language (CWL)[? ?]. Consequently, this increased variability likely poses considerable challenges for LLMs. For this, a thorough analysis of how the idiosyncrasy of the leveraged workflow language influences the outcomes produced by LLMs would be beneficial in offering guidance to practitioners.

Explored Use Cases

This work focused on comprehending, modifying, and extending workflows with ChatGPT. These use cases represent only a partial scope of user support opportunities and are worth considering and evaluating other use cases. For instance, migrating workflows implemented in legacy workflow management systems to more recent ones, e.g., transforming Taverna [?] scripts to Snakemake or Nextflow, or adapting them to different infrastructure stacks poses an interesting research question. Moreover, user support in workflow debugging, error identification, or optimization, as done

in classical programming [?], would be a valuable contribution to research scientists.

Conclusion

The significance of large-scale data analysis workflows in advancing research in the natural sciences is growing steadily. Developers of such workflows, primarily researchers from diverse scientific fields, are challenged with the increasing complexity and scale of their analyses, which involve (next to their domain knowledge) working with different frameworks, tools, programming languages, and infrastructure stacks. Although a few tools for creating and maintaining workflows are available, improving user efficiency remains an open research area. In this work, we contribute to this situation by evaluating the suitability of ChatGPT for comprehending, modifying, and extending scientific workflows. In three user studies with four researchers from different scientific domains, we evaluated the correctness of ChatGPT regarding explainability, exchange of software components, and extension when providing real-world scientific workflow descriptions. Our results show a high accuracy for comprehending and explaining scientific workflows while achieving a reduced performance for modifying and extending workflow descriptions. These findings clearly illustrate the need for further research in this area.

Additional Files

Supplementary Material: Complete Questionnaires for user studies I-III, schema of workflow *WF4-Grasslands*, verbal task description for user study III and statistics of the GitHub Search results.

Data Availability

The source code of all investigated workflows are available via [? ? ? ? ?]. We provide the complete LLM chat outputs as supplementary material to this manuscript via the GigaScience database (GigaDB) [?].

Declarations

List of Abbreviations

LLM: Large Language Model; EUP: End-User Programming

Competing Interests

The authors declare that they have no known competing financial interests or personal relationships that could have appeared to influence the work reported in this paper.

Funding

This work is supported by *German Research Foundation (DFG)*, CRC 1404: "FONDA: Foundations of Workflows for Large-Scale Scientific Data Analysis" (Project-ID 414984028).

Author’s Contributions

Mario Sänger: Conceptualization, Methodology, Investigation, Visualization, Writing - Original Draft **Ninon De Mecquenem**: Resources, Data Curation, Writing - Review & Editing **Katarzyna Ewa Lewińska**: Resources, Data Curation, Writing - Review & Editing **Vasilis Bountris**: Resources, Data Curation, Writing - Review & Editing **Fabian Lehmann**: Resources, Data Curation, Writing - Review & Editing **Ulf Leser**: Conceptualization, Writing - Review & Editing, Project administration, Funding acquisition **Thomas Kosch**: Conceptualization, Methodology, Writing - Original Draft , Writing - Review & Editing, Validation, Project administration

Click here to access/download;Figure;wf4_grasslands_fig.pdf

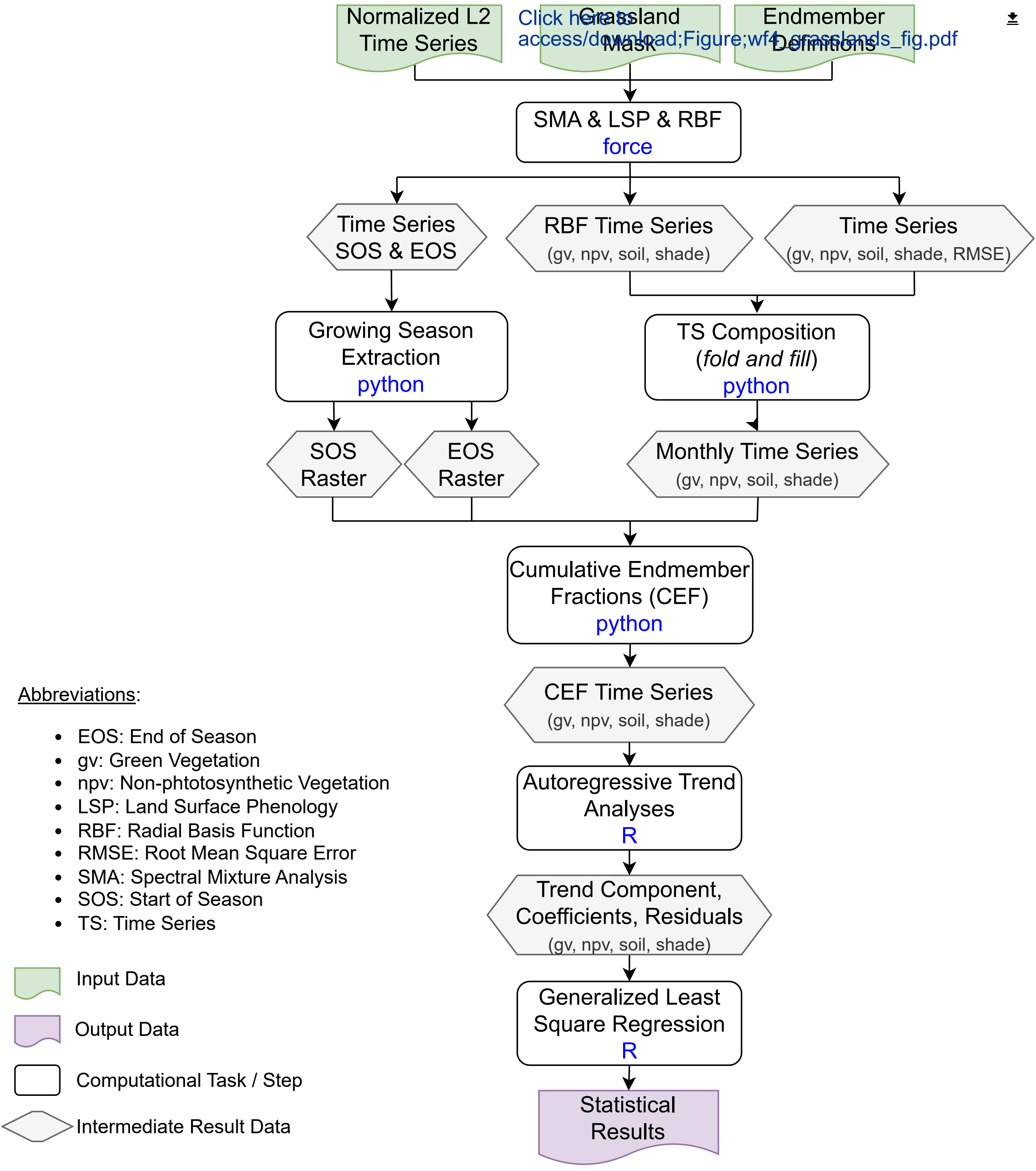

Click here to access/download
Supplementary Material
supplementary_material.pdf
