# Supplementary material for: A qualitative assessment of using ChatGPT as large language model for scientific workflow development
Source: Gigascience. 2024 Jun 19;13:giae030. doi: 10.1093/gigascience/giae030 (PMC11186067; doi:10.1093/gigascience/giae030)
Supplement: Response_to_Reviewer_Comments_Original_Submission [file response_to_reviewer_comments_original_submission.pdf]

Author's Response To Reviewer Comments

Our responses to the comments raised can be found in the reply letter (contained in revision_letter.pdf).
