# Supplementary material for: A qualitative assessment of using ChatGPT as large language model for scientific workflow development
Source: Gigascience. 2024 Jun 19;13:giae030. doi: 10.1093/gigascience/giae030 (PMC11186067; doi:10.1093/gigascience/giae030)
Supplement: Response_to_Reviewer_Comments_Revision_1 [file response_to_reviewer_comments_revision_1.pdf]

Author's Response To Reviewer Comments

Dear editor,

thank you again for giving us the opportunity to publish our research in your journal. We revised the manuscript according to your comments.

If you need further information or support, do not hesitate to contact us.

Best,
Mario & Thomas
