# Supplementary material for: A qualitative assessment of using ChatGPT as large language model for scientific workflow development
Source: Gigascience. 2024 Jun 19;13:giae030. doi: 10.1093/gigascience/giae030 (PMC11186067; doi:10.1093/gigascience/giae030)
Supplement: Reviewer_1_Report_Original_Submission — Tazro Ohta -- 2/8/2024 [file reviewer_1_report_original_submission.pdf]

Reviewer Report

Title: A Qualitative Assessment of Using ChatGPT as Large Language Model for Scientific Workflow Development

Version: Original Submission **Date:** 2/8/2024

Reviewer name: Tazro Ohta

Reviewer Comments to Author:

In this manuscript, the authors evaluate the potential and limitations of using Large Language Models (LLMs) to address current issues in the construction and operation of data analysis pipelines in the field of data science. The LLM used in this study is ChatGPT 3.5, which is one of the most popular LLMs currently available. It is reasonable and likely to attract readers' interest to publish evaluations using this model, considering its popularity. However, it is necessary to note the limitations of using ChatGPT as it is a web application provided by OpenAI, and much of its source code and technical details are not publicly available, which may restrict its use as a scientific tool. While the authors acknowledge the existence of open LLMs that can be considered equivalent to GPT3.5, they did not evaluate them in this manuscript. The authors conducted evaluations using workflows from two different domains, life sciences and earth sciences, as examples. They engaged domain experts to review and evaluate tasks of interpreting, modifying, and extending these workflows, and used survey results for evaluation. Regarding ChatGPT's performance, the authors found that while it demonstrated certain capabilities in interpreting and modifying workflows, there are limitations to its availability for extending workflows. The performance of ChatGPT appears to vary depending on the domain or the workflow system used, suggesting the possibility of performance changes based on the combination of ChatGPT with specific domains or workflow systems.

The manuscript is well-written and logically structured. The extensive logs of conversations with ChatGPT included in the supplementary material are useful for researchers interested in conducting similar studies. Although not all the chat logs I have been thoroughly reviewed, it seems that at least the responses for "WF1 crisprseq" and "WF2: RS-Star" are valid. Additionally, I confirmed that the same prompts given to ChatGPT 3.5 produced nearly identical responses. Considering the anticipated advancements in LLM-related technologies, the paper is important in terms of assessing the current accuracy of LLMs and raising future challenges.

The following are comments and requests regarding the manuscript from the perspective of a researcher operating and developing workflow systems in the life sciences field:

1. Concerns are raised regarding the suitability of the title for readers, particularly whether it might be misunderstood as targeting beginners with limited workflow or programming experience, despite the authors are aiming to reduce the workflow development cost for domain experts. I suggest to improve the title so the readers can expect the target users and the potential use cases that LLMs can rescue. (My kids would love the current one though)

2. To support the consideration that misunderstanding of expanded abbreviations impedes workflow comprehension, I suggest to attempt inserting expanded forms of abbreviations used in prompts to see

if it improves output quality.
3. It is questioned whether it is feasible for workflow descriptions to directly answer research questions, at least in genome science. When I tried to replicate the results by using the same prompt, ChatGPT generated very different answers for the questions P1_5, while the answers can be considered as valid from my experience of the genomic data analysis. If workflows are to be tasked with answering such questions, accompanying documentation such as README files or publications should be provided in a real use case. Consider mentioning the limitation of using only workflow description, or the authors may be able to try adding the README in the context.
4. It should be noted that ChatGPT's responses vary and may change with each attempt, which is attributed to the creativity (temperature) setting of LLMs. I suggest that limitations of this variability should be described, including potential internal variations in GPT3.5.
5. Regarding Study II's workflow modification, I suggest that the authors to mention that the bulk RNA-seq pipelines are relatively matured in genomics and with the high availability of similar software. I suspect that ChatGPT can generate excessive software listings, possibly with wrong suggestions, for minor applications.
6. To support workflow developers, it's essential to consider not only the intended purposes behind the listed software but also the timing of their development. The software listed in Figure 4, having been developed at varying times, naturally implies that later releases may offer enhancements or cater to different needs. Therefore, prompts should prompt responses regarding the development history or simply the release date. This consideration is particularly relevant due to the limitations of our knowledge about more recent developments, stemming from the constraints of available training data.
7. Comment on the Limitation of Study Design: In the field of genomics, there are numerous benchmark papers comparing the performance of multiple software tools developed for the same purpose. It would be an intriguing challenge to see if ChatGPT can reach similar conclusions as these benchmark papers regarding workflow interpretation and alternative suggestions. By selecting workflows that have already been compared and evaluated by humans, it may be possible to conduct analyses that focus more on the capabilities of ChatGPT.
8. The focus of this evaluation was on Nextflow and Apache Airflow, but I'd like to explore the potential challenges that ChatGPT may encounter due to the distinctive language syntax of these platforms. Specifically, Nextflow utilizes a specialized DSL (Domain Specific Language), while Apache Airflow follows a Python-based syntax. Given that GPT learns from programming languages on the internet, one might expect better performance with the Python-based Airflow. However, experiments involving workflow extensions were conducted solely on Nextflow workflows. Would it be possible to include experimental results involving Airflow extensions as well?
9. Similarly, the flexibility of languages in workflow systems may potentially disadvantage language models. Workflow systems like the Common Workflow Language (CWL), which allow for more deterministic descriptions, might be easier for GPT to generate functional workflows. In fact, my colleagues and I regularly use GPT3.5 to write CWL workflows, albeit not excessively complex ones. The relationships between the nature of the given language syntax and the performance of the language models in these tasks may be worth mentioning in Discussion.
Minor comments:
1. Interpretation of distributions shown in Figure 2a and Figure 5 is deemed difficult. I suggest the use of

simpler bar plots for representing response frequencies.
2. I understand Figure 2b represents the number of valid responses. It is misleading having the 0.5 increment on the Y-axis.
3. The manuscript has some missing references, probably bugs from PDF compilation. Please check the followings:
- p5 In the "LLM Prompting" both two sentences lack Section references as "We refer to Section for a detailed discussion of prompt design challenges." and "study design and prompt strategy selection in more detail in Section ."
- p6 Figure 2a legend lacks a reference in "(see)"
- p7 "The complete questionnaire is given in ."

Methods

Are the methods appropriate to the aims of the study, are they well described, and are necessary controls included? Choose an item.

Conclusions

Are the conclusions adequately supported by the data shown? Choose an item.

Reporting Standards

Does the manuscript adhere to the journal's guidelines on minimum standards of reporting? Choose an item.

Choose an item.

Statistics

Are you able to assess all statistics in the manuscript, including the appropriateness of statistical tests used? Choose an item.

Quality of Written English

Please indicate the quality of language in the manuscript: Choose an item.

Declaration of Competing Interests

Please complete a declaration of competing interests, considering the following questions:

- Have you in the past five years received reimbursements, fees, funding, or salary from an organisation that may in any way gain or lose financially from the publication of this manuscript, either now or in the future?
- Do you hold any stocks or shares in an organisation that may in any way gain or lose financially from the publication of this manuscript, either now or in the future?

- Do you hold or are you currently applying for any patents relating to the content of the manuscript?
- Have you received reimbursements, fees, funding, or salary from an organization that holds or has applied for patents relating to the content of the manuscript?
- Do you have any other financial competing interests?
- Do you have any non-financial competing interests in relation to this paper?

If you can answer no to all of the above, write 'I declare that I have no competing interests' below. If your reply is yes to any, please give details below.

I declare that I have no competing interests

I agree to the open peer review policy of the journal. I understand that my name will be included on my report to the authors and, if the manuscript is accepted for publication, my named report including any attachments I upload will be posted on the website along with the authors' responses. I agree for my report to be made available under an Open Access Creative Commons CC-BY license (http://creativecommons.org/licenses/by/4.0/). I understand that any comments which I do not wish to be included in my named report can be included as confidential comments to the editors, which will not be published.

Choose an item.

To further support our reviewers, we have joined with Publons, where you can gain additional credit to further highlight your hard work (see: https://publons.com/journal/530/gigascience). On publication of this paper, your review will be automatically added to Publons, you can then choose whether or not to claim your Publons credit. I understand this statement.

Yes Choose an item.
