# Supplementary material for: A qualitative assessment of using ChatGPT as large language model for scientific workflow development
Source: Gigascience. 2024 Jun 19;13:giae030. doi: 10.1093/gigascience/giae030 (PMC11186067; doi:10.1093/gigascience/giae030)
Supplement: Reviewer_2_Report_Original_Submission — Konstantinos Krampis, PhD -- 2/12/2024 [file reviewer_2_report_original_submission.pdf]

Reviewer Report

Title: A Qualitative Assessment of Using ChatGPT as Large Language Model for Scientific Workflow Development

Version: Original Submission **Date:** 2/12/2024

Reviewer name: Konstantinos Krampis, PhD

Reviewer Comments to Author:

-- Strengths:
The research design is robust, employing studies to evaluate ChatGPT's performance across various aspects of scientific workflow comprehension and modification. The study incorporates a detailed evaluation approach, including expert feedback questionnaires and quantitative assessments, to gauge ChatGPT's accuracy in understanding domain-specific information within workflows. By focusing on real-world workflows from the bioinformatics domain, the study provides practical insights into the applicability of LLMs
-- Suggestions for revision:
Given the identified challenges in modifying and extending workflow descriptions using ChatGPT, I suggest delving deeper into the specific limitations encountered in complex scenarios. By conducting a more detailed analysis of the factors hindering effective workflow analysis by ChatGPT, you can pinpoint areas for improvement and potential solutions to enhance LLM capabilities in this critical aspect of scientific workflow manipulation.
While your study focuses on bioinformatics workflows, I recommend considering the inclusion of workflows from additional scientific domains to broaden the scope of your research. Comparing LLM performance across diverse research contexts can provide valuable insights into the generalizability of your findings and the adaptability of ChatGPT to different scientific disciplines.
Engaging further with workflow developers to co-create and refine prompts for ChatGPT evaluation can enhance the relevance and accuracy of your study. Collaborating with experts in workflow development can provide valuable insights into the specific challenges and requirements of scientific workflows, leading to more tailored and effective assessments of LLM performance.
-- Some suggestions to expand the discussion of the paper:
Challenges in Workflow Modification: While ChatGPT demonstrates proficiency in comprehending workflows, the study reveals limitations in modifying and extending workflow descriptions, particularly in complex scenarios. This aspect underscores the current constraints of LLMs in more advanced tasks within scientific workflow manipulation. Discussing the LLM capabilities for workflow generation, analysis and modification across diverse scientific domains as extrapolated by the results of this study would be of benefit to the readers.

Methods

Are the methods appropriate to the aims of the study, are they well described, and are necessary controls included? Choose an item.

Conclusions

Are the conclusions adequately supported by the data shown? Choose an item.

Reporting Standards

Does the manuscript adhere to the journal's guidelines on minimum standards of reporting? Choose an item.

Choose an item.

Statistics

Are you able to assess all statistics in the manuscript, including the appropriateness of statistical tests used? Choose an item.

Quality of Written English

Please indicate the quality of language in the manuscript: Choose an item.

Declaration of Competing Interests

Please complete a declaration of competing interests, considering the following questions:

- Have you in the past five years received reimbursements, fees, funding, or salary from an organisation that may in any way gain or lose financially from the publication of this manuscript, either now or in the future?
- Do you hold any stocks or shares in an organisation that may in any way gain or lose financially from the publication of this manuscript, either now or in the future?
- Do you hold or are you currently applying for any patents relating to the content of the manuscript?
- Have you received reimbursements, fees, funding, or salary from an organization that holds or has applied for patents relating to the content of the manuscript?
- Do you have any other financial competing interests?
- Do you have any non-financial competing interests in relation to this paper?

If you can answer no to all of the above, write 'I declare that I have no competing interests' below. If your reply is yes to any, please give details below.

I declare that I have no competing interests

I agree to the open peer review policy of the journal. I understand that my name will be included on my report to the authors and, if the manuscript is accepted for publication, my named report including any attachments I upload will be posted on the website along with the authors' responses. I agree for my

report to be made available under an Open Access Creative Commons CC-BY license (http://creativecommons.org/licenses/by/4.0/). I understand that any comments which I do not wish to be included in my named report can be included as confidential comments to the editors, which will not be published.

Choose an item.

To further support our reviewers, we have joined with Publons, where you can gain additional credit to further highlight your hard work (see: https://publons.com/journal/530/gigascience). On publication of this paper, your review will be automatically added to Publons, you can then choose whether or not to claim your Publons credit. I understand this statement.

Yes Choose an item.
